# Biospytial: spatial graph-based computing for ecological Big Data

**DOI:** 10.1093/gigascience/giaa039

**Published:** 2020-05-11

**Authors:** Juan M Escamilla Molgora, Luigi Sedda, Peter M Atkinson

**Affiliations:** 1 Lancaster Environment Centre, Lancaster University, Library Avenue, Lancaster, LA1 4YQ, UK; 2 Centre for Health Informatics, Computing and Statistics (CHICAS), Lancaster Medical School, Faculty of Health and Medicine, Furness Building, Lancaster University, Lancaster, LA1 4YQ, UK; 3 Lancaster Medical School, Faculty of Health and Medicine, Lancaster University, Furness Building, Lancaster, LA1 4YQ, UK; 4 Faculty of Science and Technology, Lancaster University, Old Engineering Building, Lancaster, LA1 4YQ, UK

**Keywords:** spatial data infrastructure, biodiversity informatics, ecological knowledge engine, big ecological data, open science

## Abstract

**Background:**

The exponential accumulation of environmental and ecological data together with the adoption of open data initiatives bring opportunities and challenges for integrating and synthesising relevant knowledge that need to be addressed, given the ongoing environmental crises.

**Findings:**

Here we present Biospytial, a modular open source knowledge engine designed to import, organise, analyse and visualise big spatial ecological datasets using the power of graph theory. The engine uses a hybrid graph-relational approach to store and access information. A graph data structure uses linkage relationships to build semantic structures represented as complex data structures stored in a graph database, while tabular and geospatial data are stored in an efficient spatial relational database system. We provide an application using information on species occurrences, their taxonomic classification and climatic datasets. We built a knowledge graph of the Tree of Life embedded in an environmental and geographical grid to perform an analysis on threatened species co-occurring with jaguars (Panthera onca).

**Conclusions:**

The Biospytial approach reduces the complexity of joining datasets using multiple tabular relations, while its scalable design eases the problem of merging datasets from different sources. Its modular design makes it possible to distribute several instances simultaneously, allowing fast and efficient handling of big ecological datasets. The provided example demonstrates the engine’s capabilities in performing basic graph manipulation, analysis and visualizations of taxonomic groups co-occurring in space. The example shows potential avenues for performing novel ecological analyses, biodiversity syntheses and species distribution models aided by a network of taxonomic and spatial relationships.

## Introduction

The IT revolution has created the opportunity to compute, store, and transfer massive amounts of information. It is estimated that the volume of all digital information will surpass 175 zettabytes (ZB) (1 ZB = 10^21^ bytes) by 2020 [1]. In addition, the growth in data follows an exponential curve that doubles in volume every 2 years ([2–4]). Moreover, this expansion in data production has occurred in all human activities, including the environmental sciences. Novel approaches for measuring natural processes are being applied, adding more reliable and diverse data, and environmental measurements cover a wide range of spatial and temporal scales ranging, for example, from long-term ecological experimental plots [5, 6] to near-real time imagery from Earth observation satellite systems such as NASA’s Joint Polar Satellite System [7] and ESA’s Copernicus programme [8]. This IT era is opening new opportunities for greater understanding of nature. For example, pervasive Internet connectivity has made possible the transfer of data across large distances in a short time, and the multifunctional capabilities of mobile and smart devices have enabled the management and deployment of collaborative surveys at low marginal costs. Geospatial sciences have benefited in particular. Methodologies for collecting, annotating, and curating these new sources of spatial data have been proposed by [9–11] under the term “citizen science,” where data are collectively assembled by a community of enthusiasts and volunteers. Some iconic examples of these (crowd-based) platforms are OpenStreetMap [12] for geographic maps and the Global Biodiversity Information Facility (GBIF), an international consortium of research and governmental institutions that gathers and publishes information of all types of biodiversity occurrences [13].

The exponential growth of data imposes new challenges for storage, access, integration, and analysis. Recent years have brought new theoretical methods and technologies that are being developed to tackle these problems. “Big Data” is now an umbrella term for methods dealing with huge, complex, and heterogeneous datasets that cannot be handled with traditional methods. See [14, 15] for a review of the field and [16] for theoretical and practical challenges involving big geospatial data.

A fundamental goal in ecology is the understanding of the relationships between living beings and the environment. A requirement to achieve this goal is the integration of independent studies and measurements to validate hypotheses on potential causal relations. To test the existence of these causalities, a substantial number of inputs in terms of theory, methods, and data is needed. Moreover, reliable, reproducible, and easy-to-access methods are especially important given the urgency in addressing ongoing environmental crises (e.g., rapid ecosystem degradation, global climate change, accelerated extinctions, and biodiversity loss) [17, 18]. Ecology is thus adapting rapidly to these critical challenges and is starting to adopt and develop novel theoretical and computational methods to solve a central problem: how to synthesize and integrate ecological theory with big ecological data. Answering this question requires an interdisciplinary approach that touches many fields, including theoretical ecology, mathematical modelling, statistics, computer science, and information sciences. For example, Loreau [19] proposed a conceptual framework for integrating ecological theory by centering evolution as the link to unify ecology; and Pavoine and Bonsall [20] proposed a semantic and mathematical formalization for unifying traits, species, and phylogenetic diversity. The 2 approaches exemplify how evolutionary (ancestry) relationships between biological objects constitute a solid base to unify distant branches of ecology. From a statistical perspective, meta-analysis has been effective in synthesizing research evidence across independent studies, including unveiling general relations through a statistically sound framework [21].

Geospatial data constitute a crucial component for data fusion and harmonization; see [22] for a review of methods for heterogeneous spatial Big Data fusion, and [23] in order to remove bias by using spatial data stratification methods. A clear example of geospatial data fusion is the building of essential biodiversity variables (EBVs) to identify biodiversity and ecosystem change [24]. EBVs constitute a minimal set of critical variables aimed to standardize and harmonize global biodiversity variables. Originally proposed by the Group on Earth Observations Biodiversity Observation Network (GEO BON) to assess biodiversity change globally [25], EBVs are now being used to predict global species distributions and potential scenarios for policy options [26]. EBVs integrate data in a standardized framework that describes spatial, temporal, and biological organization [27]. Recently, methodologies for building EBVs have been drawing the attention of interdisciplinary research for reliability and data quality [28]. System designs and infrastructures for integrating heterogeneous big ecological data are emerging. Examples of these are the citizen-based bird observation network (eBird [29]), the TRY database for plant traits [30], the PREDICTS project (Projecting Responses of Ecological Diversity in Changing Terrestrial Systems) [31], and the Botanical Information and Ecology Network [32]. Despite the data heterogeneity and biased information against real absences (a consequence of opportunistic sampling), these types of infrastructures are able to collect sufficient quantities of data to perform statistical inference ([33, 34]). The use of high-performance computational technologies with novel statistical methods for representing and modelling big ecological data can provide deeper understanding of biodiversity evolution and its dynamics in a changing world [25, 27, 35]. Moreover, its implications can be extended to other branches of ecology and earth sciences. For example, a process-based approach [36] showed how community assemblages can be integrated into dynamic vegetation models to increase the precision of climatic and earth system models.

From a technical perspective, environmental and ecological data often come in matrix form such that they can be stored and analysed efficiently with a relational database management system (RDBMS) or other tabular data structure. RDBMSs are reliable and sophisticated tools. An important feature is the possibility to extend their functionality with programming languages such as C, Java, Python, or R-Cran. This allows the combined use of an efficient data management system with a broad range of statistical libraries and programming methodologies. An example of this is the integration of spatial analysis tools into the RDBMS through the Postgis project [37], a set of compiled functions written in the Postgresql Procedural Language (PostgresPL) that interfaces with high-level geospatial libraries (e.g., [38–40]). Postgis adds GIS capabilities to the database engine, giving superior performance for querying information with geometric and topological features in space.

Integrating large datasets using only relational methods is computationally intensive. For example, matching data by a common feature involves the definition of join clauses plus computing the joined lookup between the pair of tables. The resulting product is often stored in volatile memory, a limiting factor when integrating large datasets. In a typical database design, table indices cost *O*(log(*n*)) in time, where *O*( · ) is the classic “Big*O*,” a measure of computational complexity, and *n* is the size of the input dataset. A query involving multiple joins (from multiple data tables) can involve reverse and recursive lookups, which can increase the load from *O*(*n*) to *O*(*n*^*k*^), where *k* is the number of data tables to join. Although this issue can be addressed with database design techniques such as normalization [41] or caching [42], the solution likely obfuscates the comprehension of the relational schema by adding unintuitive tables and other auxiliary information. It also requires a learning curve and expertise for implementation as well as increasing complexity when more datasets are added.

Data structures based on direct acyclic graphs (DAGs) are advantageous in relation to the above approaches. Traversing a relationship in a graph database has constant cost (*O*(1)) [43] if the relations are defined explicitly for every node. Whenever a new dataset is added, a new link can be created to relate it with an existing record. Graph databases, however, are not as efficient at processing geospatial queries or handling simultaneous queries [44]. In this sense, hybrid data management systems, capable of handling both paradigms (relational tables and DAGs), were proposed to overcome the limitations of both systems. However, to the best of our knowledge, these proposals have not been yet implemented [45], their code is closed [46], or their scope is not suited for environmental and spatial datasets, as is the case of the Reactome Database [47].

In this article we propose an implementation of an open source knowledge engine (i.e., a hybrid database system) that stores, accesses, and processes geospatial and temporal information, to integrate, analyse, and visualize heterogeneous environmental, EBV, and big ecological data. The engine, named “Biospytial” (composed of the words “biodiversity,” “Python,” and “spatial” and pronounced “Biospatial”), incorporates semantic relations that integrate data in a web of semantic knowledge able to represent complex graph (network) data structures.

Biospytial can be considered a component of traditional spatial data infrastructure (SDI) because we simplify access and analysis of big datasets while satisfying the need of producing information for scientists and policy makers, among others [48]. This is possible owing to the engine’s capability of identifying intrinsic and extrinsic relationships within environmental and socioeconomic processes. Therefore, the developed engine is aimed to serve SDI-based decision-making frameworks, such as, e.g., the European project INSPIRE [49].

The engine serves as a multi-purpose platform for modelling complex and heterogeneous data relationships using the power of graph theory. The current implementation uses the occurrences data from the GBIF and their updated systematic classification [50] to build the acyclic graph of the Tree of Life (ToL). To exemplify the geospatial capabilities, some EBVs such as mean monthly temperature, elevation, and mean monthly precipitation are also included in the engine. The article is structured as follows: the specification and general description of the engine is described in the next section followed by the methodology and software implementation for accessing biodiversity records arranged in a taxonomic tree. The knowledge graph of the ToL is explained with examples for traversing and extracting spatial and taxonomic sub-networks. A tutorial explores the capabilities of the engine with a practical demonstration. This section shows the syntax and discusses ways to interpret and traverse the knowledge graph, ending with general conclusions and future research directions.

## An Open Source Graph-Based Engine for Geospatial Analysis

The engine is able to import, organize, analyse, and visualize big ecological datasets using the power of graph theory. It performs geospatial and temporal computations to synthesize information in different forms. The data can be queried and aggregated according to customized specifications defined by structural patterns called “graph traversals” [51]. The software has been developed with object-relational and object-graph mappings (ORM and OGM, respectively) that use the object-oriented paradigm to abstract interrelated data into class instances [43,52]. In this sense, every record is represented as an instance of a certain class with its attributes mapped one-to-one to entries in a particular table (if it is stored in a relational database) or in a key:value hash table (if it is stored in a graph-based database). This approach allows the building of complex and persistent data structures that can represent different aspects of the knowledge base. It also allows the assembly of automatic methods for exploring, filtering, aggregating, and storing information.

### System architecture

**Figure 1:**
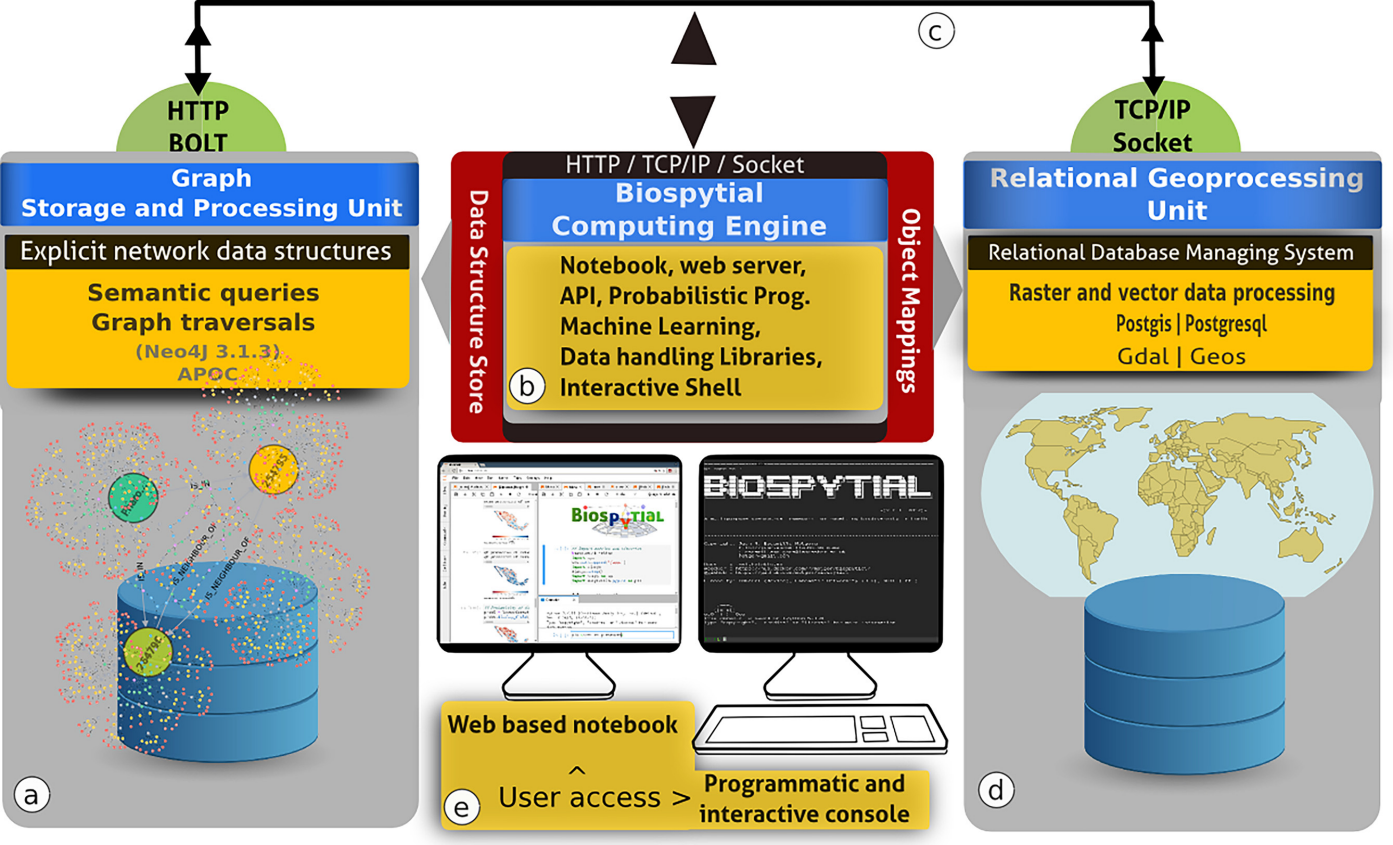
The Biospytial system with the 3 interconnected modules. (a) The GSPU, where semantic queries and graph traversals take place. (b) The BCE, where object mappings, web services, and the modelling framework take place. It includes several libraries for performing exploratory analysis as well as Bayesian statistical inference and prediction using the probabilistic programming language PYMC3. (c) All the components can be allocated in the cloud and are connected using virtual and physical networks. (d) The RGU, where the geoprocessing and spatial indexing occurs, storing efficiently any raster and vector data sources. (e) Interactive access is possible in 2 ways: using an online web notebook (Jupyter) or an interactive console (iPython).

The engine is composed of 3 interconnected modules: (i) a Relational Geoprocessing Unit (RGU), (ii) the Biospytial Computing Engine (BCE), and (iii) a Graph Storage and Processing Unit (GSPU) (see Fig. 1). Each module is arranged in virtual containers isolated as stand-alone applications [53] running a common Linux image (Debian 8) as the base operating system. The virtual container technology creates a common environment for each module, enabling the user to disregard the complications of working with heterogeneous computer infrastructures [54]. Its design allows the replication of several instances of the same module in a single computer or in a distributed network. Containerized applications are easier to replicate and migrate compared to large data volumes and databases, which often involve resource-intensive tasks in terms of energy, computing, network bandwidth, and management. The idea behind containerization is to move the processes not the data and, especially in the geospatial context, to perform spatial analysis where the data are located.

#### The Relational Geoprocessing Unit

The RGU module undertakes the storage and raster-vector processing. It relies on high-level abstractions that represent geospatial data stored in relational tables. The supported geometric features are (multi)points, (multi)lines, (multi)polygons, and multiple-band raster data. It features a fully operational Postgresql (9.4.9) server (port: 5241) with geospatial extension (Postgis 2.3.1) [37] and libraries for handling geospatial data (GDAL, OGR 1.10.1) [38], transformation between different geographic projections (PROJ 4.8 [40]), and computation of geometric operations (GEOS 3.6) [39] (Fig. 1b). The RGU image can be downloaded from [55].

#### The Graph Storage and Processing Unit

This module hosts a graph database that stores data on nodes and their relations in a network structure called the knowledge base (Fig. 1a). The graph database system is an instance of Neo4J (3.1.3), an open source ACID-compliant transactional database management system with native graph storage and processing [43]. It includes a web-based interface located in http:// < custom url >:7474 The interface allows the inspection and visualization of queries (subgraphs) using the Cypher interpreter (a No-SQL type declarative language for interrogating graph databases). The module also includes a plugin for spatial and topological lookups [56] and the Awesome Procedures on Cypher (APOC) [57], an extension library with >300 procedures for data integration, graph algorithms, or format conversion procedures. The GSPU image can be downloaded from [58].

#### The Biospytial Computing Engine

This module provides the interface and processing toolbox for accessing, exploring, and analysing data structures through the Object Mapping design. The container hosts a virtual environment and an Anaconda package manager [59] that includes all the dependencies required by the engine. The core code of the engine is contained in a new Python package called Biospytial [60] (Fig. 1c). The engine structure includes a drivers module to communfoicate with the graph database; the modules for accessing each dataset in the relational database; the module for graph traversals, data ingestion, gridding systems, vector sketching, and Jupyter notebooks; and external plugins such asspystats, a Python port of GeoR [61]. The image can be downloaded from [62].

#### Other features

##### Scalable

The implementation includes scripts for automating the engine’s deployment in a single host or in cluster mode. This mode provides a granular configuration for the allocation of resources and services in a distributed manner. For example, the BCE module can be hosted in a computer with high-performance architectures or multiprocessing (e.g., MPI) capabilities.

##### Message broker

The engine includes a messaging service (Redis [63]) that delivers information between the different components. It also serves as an in-memory data structure storage and message broker. The storage is useful for interchanging data between different platforms and languages. For example, it allows export of the results into intermediary files (e.g., CSV or DBF) for use in other software (e.g., [64, 65]).

##### Open Source—Open Contributions

The software used in all the modules has been released with open source and free software licenses, which allow users to reproduce, modify, and publish their research source code. The engine was developed using best practices for scientific computing [66], data transparency, and reproducibility [67].

#### Access to the engine

There are 2 ways of accessing the engine. One is through a command line interpreter based on the iPython console [68]. The other is with an online Jupyter notebook server [69] (localhost:8888). The Jupyter notebook is a web-based interactive Python interpreter that renders MarkDown documents, plots, and images in the browser. Analysts can create files in a notebook format (.ipdb) and share the results online. Peers can visit the notebook’s url, read the document, run the code, replicate the analysis, access the variables, import other libraries, modify the analysis, and export it into different formats (e.g., PDF, LaTeX, or HTML).

**Table 1:** Principal software components of the Biospytial Knowledge Engine System

Software name	Version	Description
**Biospytial Computing Unit**	Debian GNU/Linux 8.6	Container OS image
Conda	4.3.30	Package manager optimized for data science
Python	2.7.11	Programming language (scheduled update for v.3.x)
R-base	3.2	Language and software environment for statistical computing
Jupyter	1.0.0	Interactive web application for reproducible computational workflows
Scipy	1.01	Python library for numerical and scientific computation
Pandas	0.19	Python library for data structures and data analysis
Geopandas	0.3	Extension of Pandas to support geospatial data
GDAL	2.1	Library for converting and processing geospatial data
Shapely	1.5.16	Python library for manipulation and analysis of geometric objects in the Cartesian plane
Django	1.8.4	ORM, web framework and stand-alone server
Py2neo	3.11	A client Python library and toolkit for working with Neo4j
Pymc3	3.4.1	A Python-based probabilistic programming framework
Patsy	0.4.1	A Python library for describing statistical models
**Relational Geoprocessing Unit**	Debian GNU/Linux 8.6	Container OS image
Postgresql	9.4.9	Relational database management system
Postgis	2.3	Spatial extension for Postgresql
GDAL	1.10.1	Library for converting and processing geospatial data
GEOS	3.6	Geometric and topological library
Proj4	4.8	Coordinate transformation software
**Graph Storage and Processing Unit**	Alpine Linux 3.5	Container OS image
OpenJDK	IcedTea 3.3	Open source Java compiler and virtual machine
Neo4J	3.1.3 (C.E)	Graph database management system
APOC	3.1.3	Utilities, graph algorithms, and common procedures for Neo4j
**Message Broker**	Redis 5.0.3	A key-value data structure store

### Knowledge representation

The engine uses 2 database paradigms to store and represent data: a relational system with tables connected by primary and foreign keys and directed acyclic graphs (DAGs) where the data are stored as nodes (with associated attributes) and edges representing relations between nodes. Each node can belong to 1 or many classes. In our implementation, the relationships are semantic phrases that refer to location (e.g., “IS IN”), ancestry (“IS PARENT OF”), or topological features (“IS CONTAINED IN” or “IS NEIGHBOUR OF”). Thus, the engine uses explicit semantic relations between nodes to build a network of semantic information. The union of all these relationships is what we call a “knowledge graph.”

**Figure 2:**
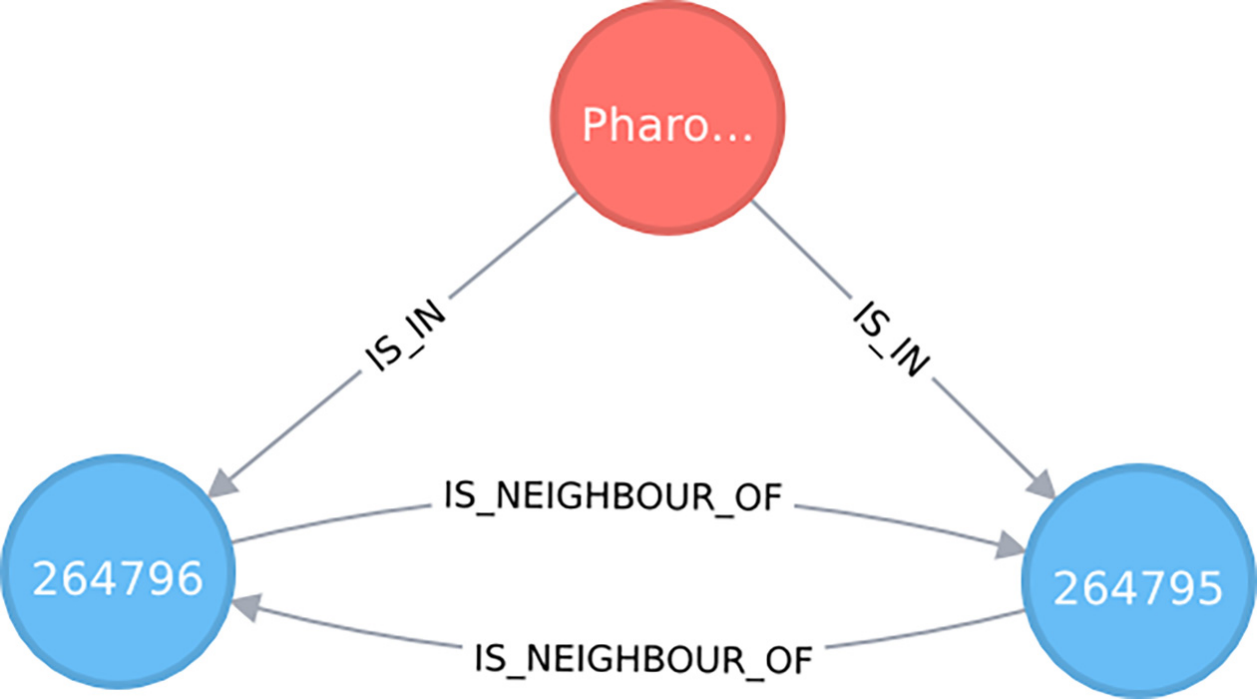
Graph showing the connection between a Species node and 2 Cell nodes. Here the species is *Pharomachrus mocinno* (quetzal) and the number shown in each Cell node is its respective ID number. This is an actual visualization taken from data stored in our knowledge graph.

The event of a species *s* being recorded at location *l* can be represented as a node of the class “Species” connected to a node *l* of class “Cell” using the relation “IS_IN”. The Cell nodes are contained in a regular lattice (grid) and are instantiated by a class that implements a geospatial type defined by a polygon that acts as a geometric border. As an example, Fig. 2 shows this diagram for the bird family of quetzales (Trogonidae) found in southeast Mexico. The node in red represents the species: *Pharomachrus mocinno*. The nodes in blue are 2 Cell types that associate the locations where *P. mocinno* was found. The arrows indicate the directional relationships between the nodes. The graph database allows easy manipulation of these nodes, their relations, and combinations. At the same time, the selected pattern can be filtered by chosen attribute values to generate customized design matrices.

### Integrating data with graph structures and object mappings

The object mapping approach serves to communicate different database management systems (relational or graph-based). A high-level Python-based Object Relational Mapping (ORM) library (Django [70]) was used to communicate with the RDBMS and the other components of the engine. It includes a high-level interface to translate sentences from the SQL declarative language into method calls from the object-oriented paradigm. Vector and raster operations are possible via the Open Source Geographic Information System (OSGIS) for Postgresql (Postgis [37]). Currently, all the spatial and tabular data are stored in the RDBMS.

The object mapping on the graph database system is achieved with py2neo, a client library and toolkit for communicating with the Neo4j database management system [71] within the Python programming language [72]. Topological information such as neighbouring cells and nodes contained within cells is stored as semantic relations. Some preprocessed information is stored in the knowledge graph. This includes some parameter estimates, aggregated data, summary statistics, and associated raster metadata.

The procedure for adding data into the engine varies according to the data format (tables or linked data) and requires a new class to be created. The class is responsible for accessing and managing data in both database systems. It includes specifications for storage, conversion between formats, and analysis. A simple implementation would include the name and type of the attributes, the name of the table (for the case of RDBMS), the node type, and incoming and outgoing relations between nodes (for graph-based datasets). Detailed information on all these procedures is given in the supplementary materials: “Adding data in Biospytial”.

### Graph traversals

As explained above, the knowledge graph is the totality of nodes and relationships stored in the database. Each node represents a type (defined by a class) of data or a more abstract concept that generalizes certain sets of data. Each node has associated edges to other nodes, as well as a list of attributes. In the example given in Fig. 2, the node is of type “Species” and one of its attributes is “name” with the associated value *P. mocinno*.

The graph engine can search and extract information from the knowledge graph using recursive rules based on semantic predicates. Typically, the search selects 1, or several, nodes and continues visiting (traversing) other connected nodes that match the specified criteria until the relationship is exhausted or a depth threshold has been reached. The resulting selection of relationships and nodes is a subgraph of the knowledge graph. We call this structure a “pattern,” and the set of rules that select a pattern is a “graph traversal.”

Graph traversals can be translated into data matrices that can be analysed within the scope of model-based geostatistics [61] or areal unit modelling in lattice systems using Gaussian Markov random fields [73–75]. Also, they can be analysed with network theory to answer questions about resilience, connectedness, modularity, or invariants across scales. The objects are compatible with the open source libraries for statistical inference and network analysis. Libraries already included in the engine are as follows: NetworkX [76], StatsModels [77], and PyMC3 [78].

#### Complex queries

Our implementation enforces the use of “lazy evaluations,” in which the evaluation of an expression is delayed until the value is needed and not performed directly upon the instantiation [79]. This helps in the creation of data primitives that can be composed into higher level graph traversals without the need to load in all the data. The design allows the request on demand of partial evaluations for a given traversal. This abstraction helps to explore, design, and automate the discovery of relevant patterns and structures. A concrete example of this design is shown in the next section with the analysis of local taxonomic trees; when the tree object is instantiated, it exists only as an abstract data container with no data requested to the database. As such, if an analyst is interested in studying the different species of bats (Order: Chiroptera) within this tree, she will need only to consider the descendant (children) nodes of the node Chiroptera of type Order (see Tutorial section for a practical example).

**Figure 3:**
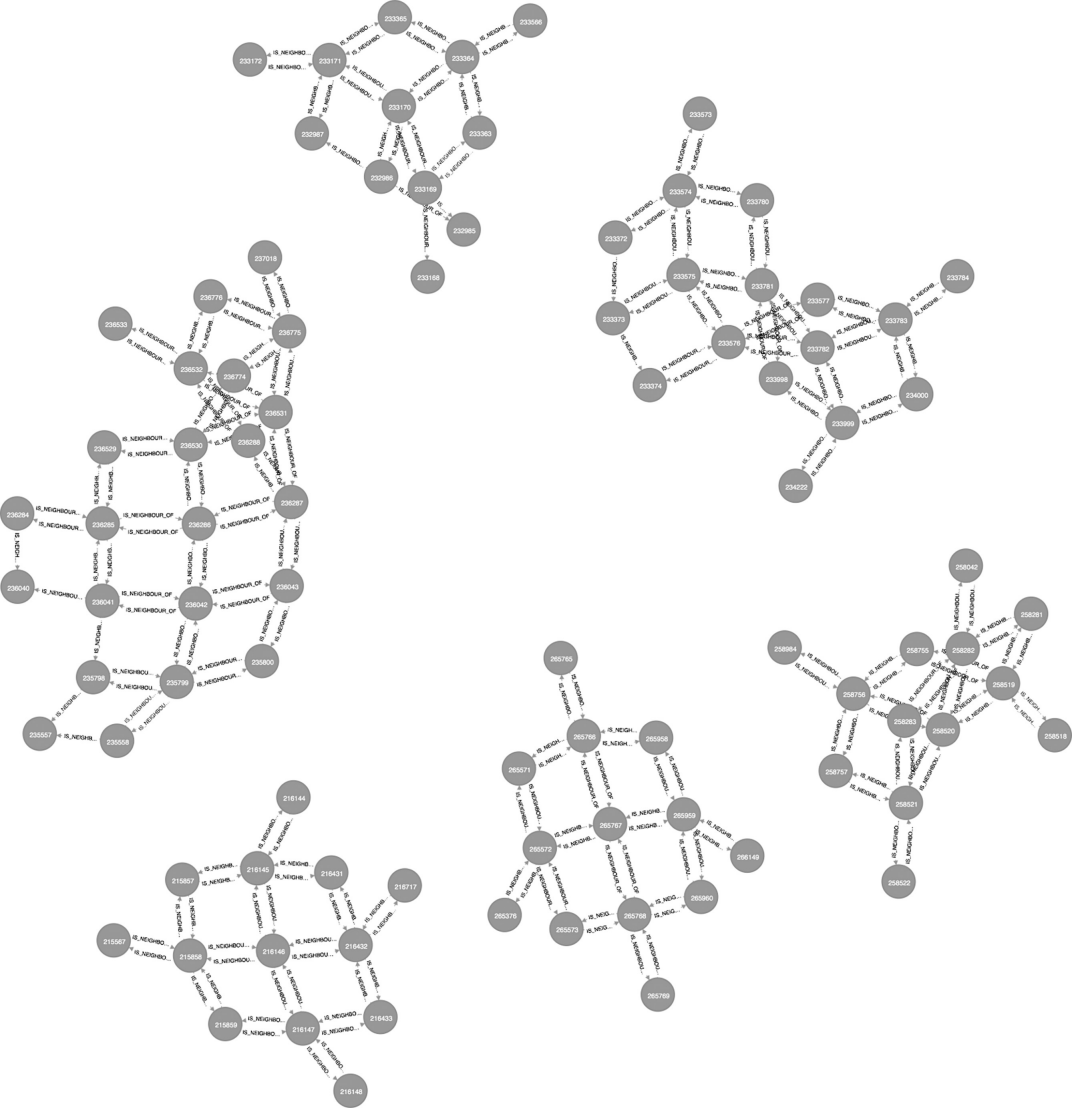
A subgraph from the knowledge engine that shows the second-order degree of neighbouring cells where ≥1 occurrence of any type of mosquito (family Culicidae) was registered. This query exemplifies the use of recursive lookups. In this case the relationship “IS_NEIGHBOUR_OF” is traversed twice.

Some traversals are exclusive of certain node classes and, therefore, have associated special methods. This is the case for nodes of type Cell, which include a method for extracting neighbouring cells. Fig. 3 shows an example of this where a selection of cells was obtained first by requesting all the occurrences of the family Culicidae and then traversing through the associated cells and their corresponding neighbours using the method getNeighbouringCells() twice.

### Geospatial management and processing

The engine supports and processes geospatial information using the GDAL/OGR library [38]. The default coordinate reference system (CRS) is the WGS84 with geographic coordinates. However, it is possible to use and reproject the data into any other CRS. This feature is supported by the Proj4 library [40]. See Tutorial section for a concrete example of this.

#### Vector data

Vector data are represented with tabular data structures. These tables should include the following information: ≥1 column with a unique identifier (ID) for each record, 1 column for each type of feature, and ≥1 geographic column to represent the geometric shape of each record. The available geometric types are points, multiple points, polylines, multiple polylines, polygons, and multiple polygons. Each type of dataset corresponds to both a vector layer and a table in the RDBMS. A mapping between the table structure and the engine needs to be created in the same way as described in engine's specification section. For large datasets the engine uses indexing methods for optimal performance on accessing and querying the data. Additional information is provided in the supplementary material: “Adding data in Biospytial - Vector data”.

#### Raster data

Raster data are represented as a table stored in the RDBMS together with its corresponding metadata. The table has 3 columns: a primary key (ID), a binary large object (BLOB) data type (encoding a stack of matrices) that represents a multiband image, and a reference to a file where the metadata are stored. The metadata includes projection type, affine parameters, datatype for entries (binary, integer, float), and other information related to provenance.

Ingesting raster data into the engine involves 2 steps: (i) the dataset is partitioned into regular tiles, and (ii) each tile is converted into a BLOB string and inserted into the table. Data ingestion scripts can be found in the supplementary materials: “Adding data in Biospytial - Add raster data”.

**Figure 4:**
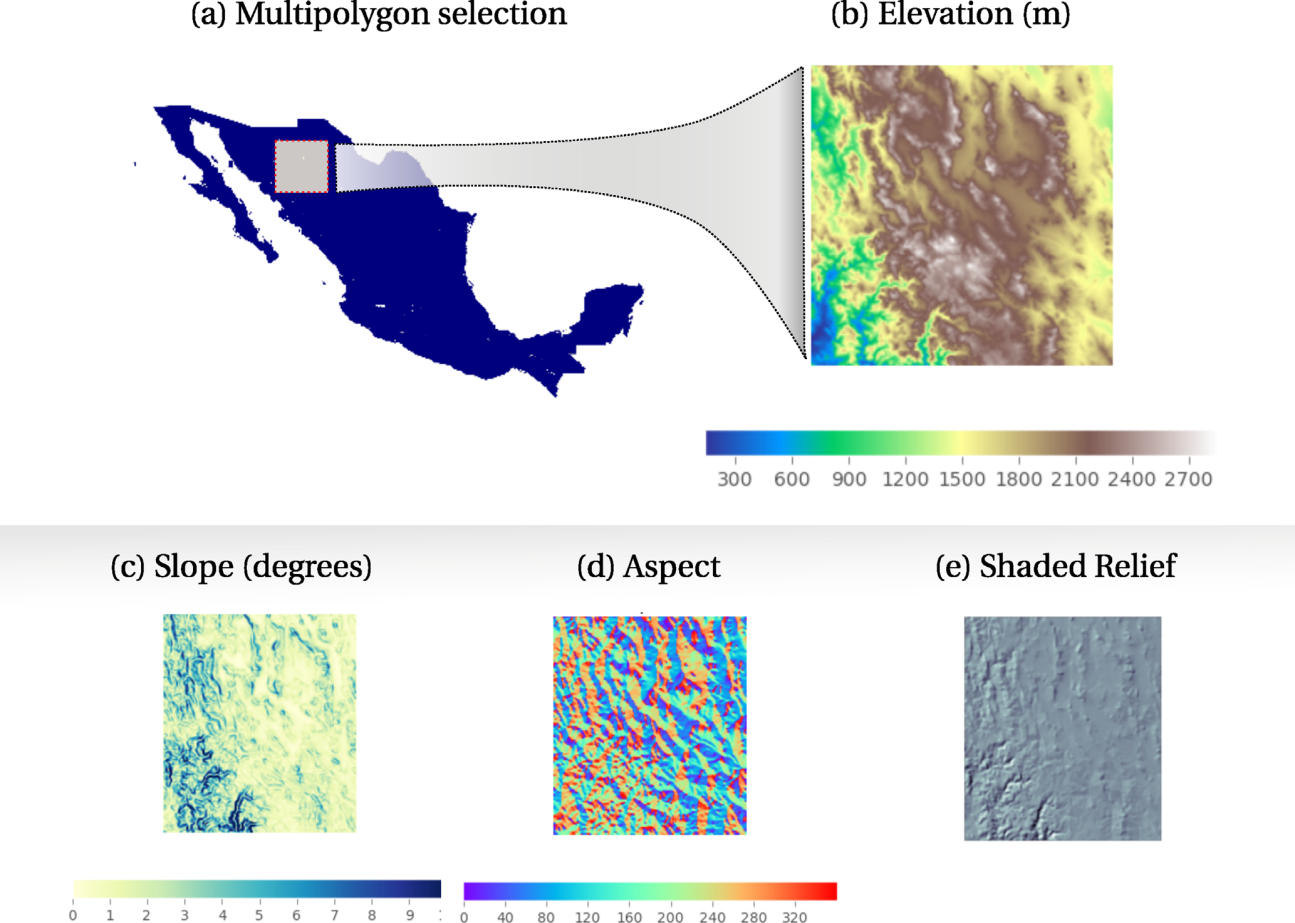
Raster manipulation in the knowledge engine. (a) A multipolygon selection corresponding to Mexico, an instance from the class Country that maps into the WorldBorders dataset. (b) An Elevation object (class RasterData) instantiated with a customized polygon, in this case a subregion of the object Mexico. (c–e) RasterData objects derived from the Elevation object. The data and visualizations were produced using the engine’s raster API .

The object mapping design is used to specify the definition of a RasterData type and its associated operations. The implemented class includes methods for clipping, downscaling, aggregating, exporting to image formats (Geotif and PNG), visualizing, intersecting vector data, extracting metadata, and conversion to arrays. An extended class for Digital Elevation Models (DEM) is also implemented to generate on-the-fly aspect, slope, and shaded relief (Fig. 4), without requiring the datasets (derived DEM products) to be stored directly in memory.

On instantiation, a RasterData object requires the definition of a boundary object passed as argument. This object should be a polygon type django.gis.contrib.GEOS.Polygon or a text string defining a polygon in the Well Known Text (WKT) format. The resulting selection can be transformed to a dataframe or *n*-array for statistical modelling. As in the other data structures, whenever a new raster model is added a new model class should be included (see Supplementary Materials: “Adding data in Biospytial - Add Raster data”).

## Using Biospytial to Analyse the Tree of Life

In this section we propose a process for integrating spatiotemporal data together with graph traversals to represent tree structures using taxonomic and topological relationships within the knowledge engine. The graph traversals use biodiversity occurrences and environmental data to build complex structures to analyse, visualize, and characterize biological occurrences in different forms. The structure restricted to the taxonomic classification is an acyclic graph (tree) in which all the species occurrences constitute leaf nodes. We call this structure the ToL and propose a set of graph traversals to retrieve subsets of the ToL constrained to arbitrary taxonomic groups, spatial regions, or temporal ranges. Several class definitions for handling taxonomic trees are implemented, making it possible to automate tasks for unveiling patterns. For a detailed definition of terms and computational structures see supplementary materials: “Mathematical formalisms”.

### Study area

The study site selected was restricted to Mexico because (i) Mexico is on the list of megadiverse countries [80,81]; (ii) the territory contains a diverse range of the world’s climatic regions [82,83]; and (iii) the country has policies for publishing open environmental data, including centralized repositories of curated data related to biodiversity, conservation, ecosystem services, land cover, and satellite sensor imagery [84]. The data in the study area provide a concrete example of the engine’s capabilities.

### Data used

The species occurrences were obtained from a snapshot taken from the global GBIF database in September 2016 [13]. The data were filtered to only include the occurrences located within the borders of Mexico. The total number of occurrences is 3,242,746 distributed in 54,828 species, 10,781 genera, 2,300 families, 543 orders, 113 classes, and 42 phyla, with acquisition years ranging from 1819 to 2016. The taxonomic classification was taken from the GBIF Taxonomy Backbone [50]. Each occurrence record has information of species name, location (point coordinates in WGS84), and acquisition date, and represents the observed presence of a certain species; therefore, it is entirely based on presence-only records.

The DEM "ETOPO1 1 Arc-Minute Global Relief Model" [85] was used at a spatial resolution of 1 minute. Precipitation, temperature (maximum, mean, and minimum), solar radiation, wind speed, and vapor pressure were obtained from the World Climatic Data WorldClim version 2 dataset [86]. Each variable is a 12-band raster model with 1 km^2^ spatial resolution that aggregates monthly average values from the years 1970 to 2000 per month, each band corresponding to 1 month. The data license for WorldClim restricts the redistribution of the data. Therefore, users need to download it and import it into the engine via an automated script:

raster_api.bash_raster_tools.migrateToPostgis.bash

The engine includes functions for generating grid systems at different spatial resolutions. When the grid system is created it stores a vector representation in the RGU and a network representation in the GSPU. The functions for generating the grid systems are located in the library mesh.tools.py.

### Traversals on the knowledge graph

The taxonomic tree structure was built with the relation IS_PARENT_OF (conversely, Has_Children) following the taxonomic classification of the occurrence data and the GBIF Backbone Taxonomy [50]. Each occurrence had a location attribute matched with environmental data (e.g., elevation or WorldClim) using a point-in-polygon query to the RGU. The spatial structure was built using the relations IS_IN and IS_CONTAINED_IN in accordance with topological relationships based on the DE-9IM model [87,88] (standardized by [89]).

**Figure 5:**
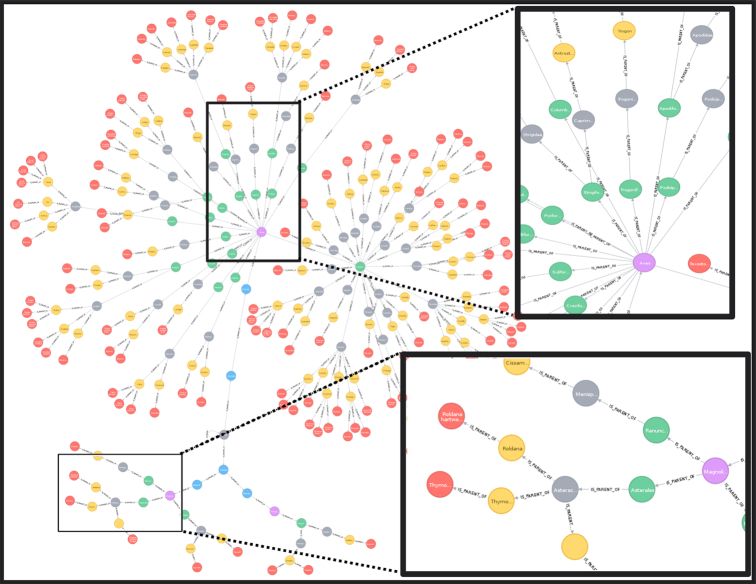
A visualization of a local taxonomic tree built with the relationship IS_PARENT_OF. The rectangles show zoomed-in areas in different sections of the tree (upper region for birds [Order Aves], lower for plants [Order Magnoliopsida]). Colored nodes indicate distinct taxonomic levels (red: species; yellow: genera; grey: families; green: orders; purple: classes).

The main traversal structure is defined in the TreeNeo class. Each instance comprises an area defined by a spatial polygon and a list of occurrences contained on it. The graph traversal was built recursively using the systematic classification of organisms, starting from the GBIF occurrences as leaf nodes and progressing through the parent nodes until the traversal reaches the node with no parent. That is, it begins at the species level and finalizes in the root node. At each step, the algorithm fetches the available nodes and groups them by their corresponding parent node, generating a set of parent nodes and their associated children. Each of these duples (parent, children) are incorporated into a LocalTree object that parses the relevant information into several attributes. This process is applied recursively on each derived parent node of the previous step. The recursion is terminated when the set of parent nodes is empty, generating the desired tree data structure. When this happens the LocalTree object is wrapped into a TreeNeo instance that extends some additional methods such as manipulating and querying trees, nodes, and multiple taxonomic groups as well as graph analysis and exportation to common exchange formats (e.g., graphml, data frames, png, geotif, or shapefiles). In addition, all the spatial structures were implemented with Open Source Geospatial (OSGEO) standards [90] to facilitate migration to other languages and platforms. A visualization of this traversal is shown in Fig. 5.

## Worked Examples

This section is a case study for analysing the frequency of coexistent taxonomic groups in the entire available dataset restricted to arbitrarily chosen branches of the ToL, included in a list of threatened species. These types of analyses are important in conservation studies, where the characterization of umbrella (or other surrogate) species constitutes the basis for protecting a significant number of associated species [91,92]. To account for this effect, we chose the jaguar (*Panthera onca*) as the species of interest. This is due to its preference for undisturbed ecosystems [93] and its wide geographic required range: 181 ± 4 km^2^ for females and 431 ± 152 km^2^ males [94].

### Additional data used

We use the International Union for Conservation of Nature Red List of Threatened Species (Red List) [95] in Mexico to account for the proportion of species (critically endangered, endangered, or vulnerable) associated with the presence of jaguars. For aggregating the data into taxonomic trees (i.e., TreeNeo objects), as well as for extracting their corresponding environmental covariates, we used a 0.05° (∼5 km) resolution grid intersected with the terrestrial regions of Mexico and Central America. The grid used is included in the default installation of the engine, and therefore, all the analysis performed in this example is reproducible.

### Methodology

We first obtain the grid cells with ≥1 occurrence of jaguar. Because these cells are Cell objects, it is possible to extract associated neighbouring cells using the method getNeighbours. We can apply the same method recursively 4 times to obtain a list of neighbouring cells within a 4-degree neighbourhood. For each cell, we obtain the local taxonomic tree. The resulting trees are merged into a single tree that contains the union of all the nodes of all the local trees. Therefore, the aggregated tree contains all the known co-occurrences of jaguar in a neighbourhood of degree 4. The resulting tree is filtered to select only the nodes that match the Red List of threatened species. A new tree object is created using the selected nodes, an operation known as “trimming.”

To provide an estimate of which nodes co-occur more often with jaguars, we rank all the nodes in the merged tree using the frequency of presence of each node at each neighbouring cell. To show the raster querying capabilities, we contrast these results with the environmental ranges of the following: jaguars, threatened species, and the entire country using the raster_api module. Finally, we provide methods for interactive visualizations of the extracted spatial data and the network structure.

### Results of the worked example

The taxonomic analysis revealed that the most abundant families across all neighbouring cells were Muridae (rodents, 29%), Phyllostomidae (a family of bats, 23%), and Cervidae (deers, 15%) for the case of mammals. For parrots (Order Psittaciformes) the most frequent species was *Ara militaris* (military macaws, 2%) and several species of the genus *Amazona*, accounting for 16% in total. Although the order Psittaciformes was abundant (23%) in the group of vertebrates, the most abundant taxon (*A. militaris*) only co-occurred 2% of the time with the jaguar’s neighbouring cells. This result shows the great diversity of species within the group of parrots. This is consistent with natural history records, where these species have been reported to inhabit humid forests, wooded foothills, and canyons in elevation ranges between 500 and 1,500 m above sea level [96].

The same analysis applied to plants showed that the most abundant genera and species were the epiphyte *Tillandsia* (19%), *Coussapoa oligocephala* (6%), *Pouteria* (several species, 9%), *Cedrela odorata* (3%), which are tropical trees, and other trees not typical from tropical rain forests such as *Oreopanax* (9%) and *Quercus* (6%). Longer lists of the most abundant taxa detailed in the worked example as well as their interactive version in the Jupyter notebook are provided in the file examples/Official Demo Co-occurrences.ipynb located in the Biospytial repository. A visualization of the threatened taxa tree is shown in Fig. 9 for kingdoms, phyla, classes, and orders.

**Figure 6:**
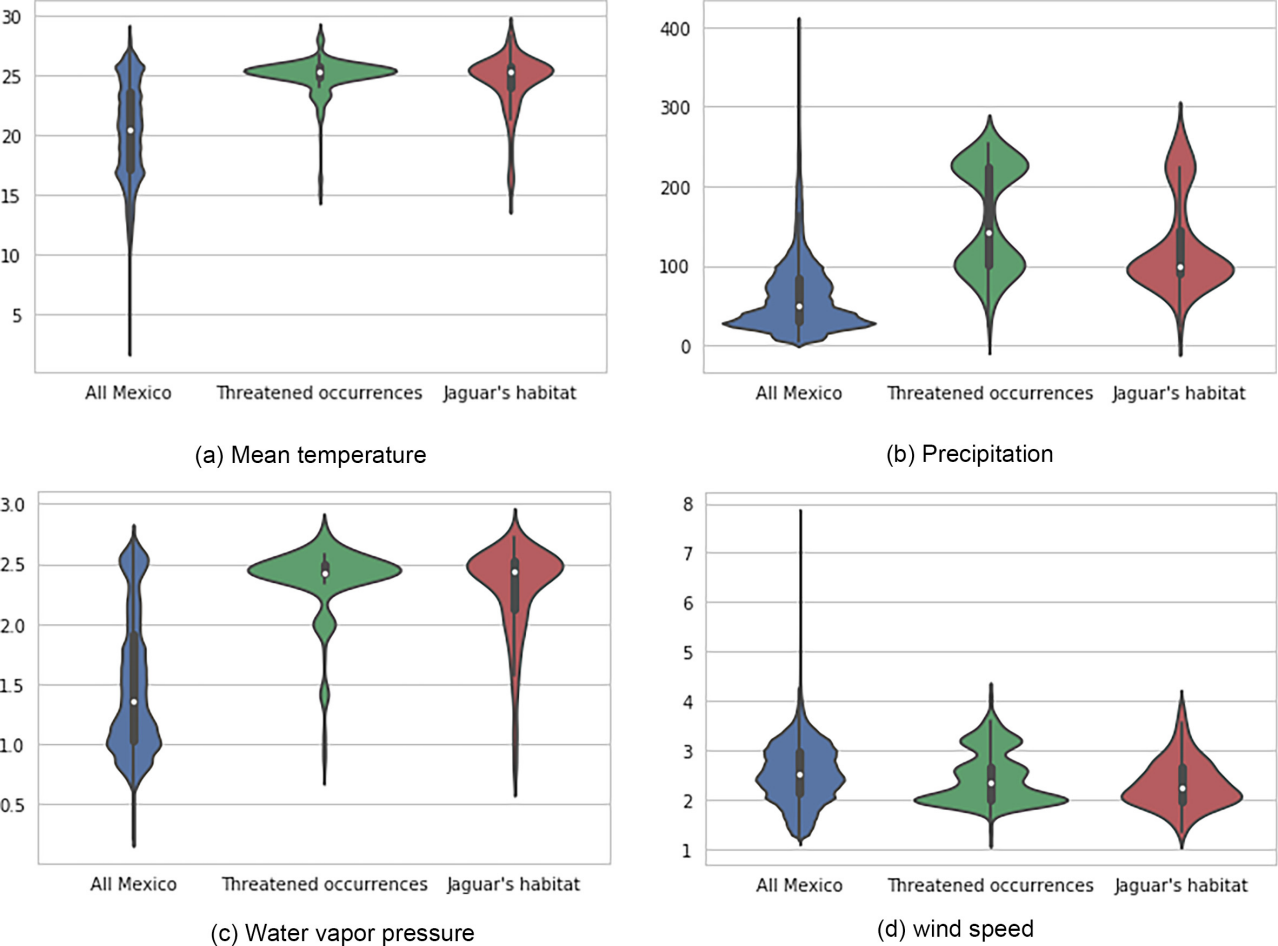
Comparison of mean annual environmental ranges between treatments: all Mexico, threatened taxa, and cells with occurrences of jaguars using violin plots. White dots indicate medians, black bars the interquartile range and stretched black lines lower and upper adjacent values.

From an environmental perspective there is a clear concordance between jaguars’ habitat and threatened taxa, when compared to all Mexico, for mean temperature (Fig. 6a), annual rainfall (Fig. 6b), and wind speed (Fig. 6d). In fact, threatened species and jaguars show environmental modalities distinct from all Mexico. To create the plots we used the Seaborn library [97]. Detailing the process for creating these graphs is out of the scope of the present tutorial. However, the snippet has been included in the interactive notebook.

## Tutorial

The time for executing the following example varies considerably depending on the group of interest, the size of the neighbourhood, and the computer platform. A quick workaround to speed up the processes is to reduce the number of neighbouring cells (order of the neighbourhood). For example using a degree of 1.

A reproducible version of this tutorial is included in the Biospytial source code (inside the folder examples/) in an interactive Jupyter notebook file named :

Official Demo Co-occurrences_jaguar.ipynb

The following section is a static version and is subject to minor modifications to fit the layout and format of this version.

### Selecting the node “Jaguar”

We begin by selecting the node in the ToL corresponding to the genus *Panthera*. This node is linked to some Species and Family type nodes and also has links to Occurrence nodes, where the information of location and time is stored. To start the traversal we need to first select this node. To do so we use the function pickNode using the following syntax:

pickNode(<Type of Node>,’name of the node’)

In the next example we see how to load the pickNode function and the appropriate node class (in this case Genus).

from drivers.graph_models import Genus, pickNode

jaguars = pickNode(Genus,”Panthera”)

The variable “jaguars” is now an instance of the class Genus. As such, it has associated attributes and methods. Its string representation is the following:

jaguars: <TreeNode type: Genus id = 2435194 name: Panthera>

We proceed to traverse through all the cells where any occurrence of the *Panthera* genus was registered. To do so we call the attribute “cells.” This attribute is abstracted with lazy evaluation. To fetch all the associated data we need to convert the object into a list (or a partial list using an iterator).

cells = list(jaguars.cells)

print(”cells has %s elements”%len(cells))

cells has 62 elements

The resulting list has cell instances, each one connected to other cells by the relation "IS NEIGHBOUR OF". Accessing their related cells is achieved by the method:

cell.getNeighbours(with_center=[Boolean],order=[Int])

where the parameter with_center returns the center of the neighbourhood, and the parameter order the size (in number of cells) of the neighbourhood (this value can be reduced to 1 for faster computation). In our case, we apply this method for each cell using a map function with a lambda expression.

neighbours = map(lambda cell:

cell.getNeighbours(with_center=True,order=4),

cells)

“Lambda expressions” are part of Python [98] and are used to create anonymous functions. The “map-lambda” technique allows the definition of statements that are applied to all the elements of a list, returning a new list of objects obtained by evaluating the lambda expression on every element of the given list. Along this tutorial, the map-lambda technique is frequently used. Whenever this expression comes it is recommended to read the form:

map(lambda x: <something involving x> , some_list)

as, "“for all *x* in some_list, do something involving *x*". In the example above, the object neighbours is a list of neighbouring cells obtained from the method getNeighbours , available on each cell instance (i.e., each element of the cells list).

Because this list is composed of list-type elements (i.e., it is a nested list), we need to reduce it into a single list composed of only cell instances, a process known as flattening. To do this simply reduce the list as follows.

# the + operator between 2 list instances merges them together.

neighbours = reduce(lambda list_a , list_b: list_a + list_b, neighbours)

The “reduce” function is a Python standard function that receives a 2-parameter function (in this case a lambda expression receiving parameters list_a and list_b) and the nested list neighbours. The reduce function applies the lambda expression to the first pair of elements of the list and iteratively applies the result to the next element. As the sum operation between lists (+) merges the elements of both lists into a single list, performing this operation across the entire nested list neighbours results in a flattened list.

The resulting neighbours list now has 2,497 Cell nodes. In the current implementation the name of the Grid (where all the Cells are contained) is called “mex4km”. We can display the first 3 elements as:

neighbours[:3]

[< Cell-mex4km id = 234686 >,

< Cell-mex4km id = 234685 >,

< Cell-mex4km id = 234684 >]

### Converting cells to local taxonomic trees

We obtain the ToL inside each Cell node by extracting the occurrences inside each cell (using the method occurrencesHere) and plugging them into the TreeNeo constructor. The name “TreeNeo” is used because the storage backend is the Neo4j graph database.

from drivers.tree_builder import TreeNeo

cell_1 = neighbours[1]

tree_1 = TreeNeo(cell_1.occurrencesHere())

print(tree_1)

<LocalTree Of Life | Root: LUCA - n.count: 1062- >

The n.count value indicates the number of total occurrences. We can generate all the trees iteratively using a mapping from the TreeNeo(cell.occurrencesHere()) through all neighbouring cells. This may take some time depending on the number of cells and occurrences on each cell. For reducing this time see subsection: “Selecting the node Jaguar”.

sample_trees = map(lambda cell: TreeNeo(cell.occurrencesHere()),neighbours)

As in the last example, we can see such basic information as object description. Here the first 4 elements are shown.

sample_trees[:4]

[<LocalTree Of Life | Root: LUCA - n.count: 3- >,

<LocalTree Of Life | Root: LUCA - n.count: 1062- >,

<LocalTree Of Life | Root: LUCA - n.count: 151- >,

<LocalTree Of Life | No record available: - n.count: 0- >]

The value n.count indicates the number of occurrences found for the present node. It is possible to have empty trees, when no occurrences were found. This is shown with the text No record available.

### Exploratory analysis on a single tree

We select a tree in this example and explore informative data.

tree = sample_trees[1]

The object tree wraps the entire tree structure. All tree objects have as their starting node the root of the Taxonomic Tree, representing all known life.

root = tree.node

root node is similar to Family node, Genus node, etc. They all belong to the class TreeNode. We can access a specific child node with the prefix to_[name of taxon].

For example, accessing the node "Animalia" can be done as follows:

animalia = root.to_Animalia

print(animalia)

<LocalTree | Kingdom: Animalia - n.count: 742- | AF: 0.05>

#### Traverse by child nodes

We can concatenate this method until the children attribute is empty. If running Biospytial in an interactive session (like a Jupyter notebook or iPython), we can use the key [TAB] to autocomplete and show the available nodes. For example, the family of rodents Muridae:

print(root.to_Animalia.to_Chordata.to_Mammalia.to_Rodentia.to_Muridae)

<LocalTree | Family: Muridae - n.count: 34- | AF: 0.05>

#### Tree traversal by taxonomic level

The taxonomic levels (e.g., families, orders) are stored as attributes of the TreeNeo class. For example, to see the available phyla in this tree do the following:

print(tree.phyla)

[<LocalTree | Phylum: Chordata - n.count: 740- | AF: 0.05 >,

<LocalTree | Phylum: Arthropoda - n.count: 2- | AF: 0.05 >,

<LocalTree | Phylum: Bryophyta - n.count: 99- | AF: 0.05 >,

<LocalTree | Phylum: Magnoliophyta - n.count: 175- | AF: 0.05 >,

<LocalTree | Phylum: Mycetozoa - n.count: 46- | AF: 0.05 >]

and for some families inside this tree:

print(tree.families[:5]

[<LocalTree | Family: Menispermaceae - n.count: 3- | AF: 0.05 >,

<LocalTree | Family: Piperaceae - n.count: 7- | AF: 0.05 >,

<LocalTree | Family: Lauraceae - n.count: 2- | AF: 0.05 >,

<LocalTree | Family: Acanthaceae - n.count: 7- | AF: 0.05 >,

<LocalTree | Family: Plantaginaceae - n.count: 1- | AF: 0.05 >]

### Tree operations

Tree objects allow symbolic operations for adding (merging) and intersecting other tree objects. These operations are currently implemented as sum (+) and intersection (&). These operations can be applied to an arbitrary number of trees, and they are useful in comparative studies that require the calculus of (α, β, γ)-diversity using a combination of these operations [99]. Mathematically, these operations are equivalent to set operations acting at the occurrence level. As an example consider the following: let t1 and t2 be 2 trees from the list of sampled_trees, i.e.,

t1 = sample_trees[1]

t2 = sample_trees[2]

#### Addition

Adding trees is equivalent to merging them. That is, performingunion of all the nodes (internodes and leaves). The tree objects (TreeNode and TreeNeo classes) allow the use of the + operation. For example, the merged tree of t1 and t2 is obtained as follows:

t3 = t1 + t2

We can see the effect of this by selecting the nodes of a certain taxonomic level, e.g., the classes of t1 and t2 are as follows:

print(t1.classes)

[<LocalTree | Class: Myxomycetes - n.count: 46- | AF: 0.05 >,

<LocalTree | Class: Bryopsida - n.count: 99- | AF: 0.05 >,

<LocalTree | Class: Amphibia - n.count: 1- | AF: 0.05 >,

<LocalTree | Class: Aves - n.count: 667- | AF: 0.05 >,

<LocalTree | Class: Reptilia - n.count: 2- | AF: 0.05 >,

<LocalTree | Class: Mammalia - n.count: 70- | AF: 0.05 >,

<LocalTree | Class: Liliopsida - n.count: 36- | AF: 0.05 >,

<LocalTree | Class: Magnoliopsida - n.count: 139- | AF: 0.05 >,

<LocalTree | Class: Insecta - n.count: 2- | AF: 0.05 >]

print(t2.classes)

[<LocalTree | Class: Protosteliomycetes - n.count: 2- | AF: 0.05 >,

<LocalTree | Class: Myxomycetes - n.count: 112- | AF: 0.05 >,

<LocalTree | Class: Agaricomycetes - n.count: 4- | AF: 0.05 >,

<LocalTree | Class: Liliopsida - n.count: 8- | AF: 0.05 >,

<LocalTree | Class: Magnoliopsida - n.count: 25- | AF: 0.05 >]

print(t3.classes)

[<LocalTree | Class: Protosteliomycetes - n.count: 2- | AF: 0.05 >,

<LocalTree | Class: Myxomycetes - n.count: 158- | AF: 0.05 >,

<LocalTree | Class: Agaricomycetes - n.count: 4- | AF: 0.05 >,

<LocalTree | Class: Bryopsida - n.count: 99- | AF: 0.05 >,

<LocalTree | Class: Amphibia - n.count: 1- | AF: 0.05 >,

<LocalTree | Class: Aves - n.count: 667- | AF: 0.05 >,

<LocalTree | Class: Reptilia - n.count: 2- | AF: 0.05 >,

<LocalTree | Class: Mammalia - n.count: 70- | AF: 0.05 >,

<LocalTree | Class: Liliopsida - n.count: 44- | AF: 0.05 >,

<LocalTree | Class: Magnoliopsida - n.count: 164- | AF: 0.05 >,

<LocalTree | Class: Insecta - n.count: 2- | AF: 0.05 >]

#### Intersection

Intersection is applied through the & operation, and it is equivalent to the intersection of sets applied only to the leaf nodes, i.e., the “Occurrence” nodes. Once the leaf nodes are selected, the algorithm propagates through the parent nodes until it reaches the root node. The formalization of the data structure is presented in the supplementary materials: “Mathematical formalisms”. To obtain the intersection of 2 trees do the following:

t = t1 & t2

print(t)

<LocalTree Of Life | No record available: - n.count: 0- >

In this case, the intersection is empty because the Occurrences are overlaid in a regular lattice that partitions the space (i.e., the cells are disjoint). See supplementary materials: “Mathematical formalisms” for a formal definition.

#### Efficient addition of trees from a list of cells

We can use the sum iteratively in a folding sum to obtain a Tree object representing all the areas defined in a list of Cells.

big_tree = reduce(lambda a , b: a+b , sample_trees)

However, this method is not efficient. In each step, a new tree is created and the internal logic to generate the union of all the intermediate nodes can result in redundant calculations. It is much faster to select first the occurrences for all the trees inside a list and then plug them into the TreeNeo constructor, as in the example below.

# Faster version

ocs = map(lambda s: s.occurrences,sample_trees)

## ocs is a nested list.

## We need to flatten this into a single list of occurrences

ocs = reduce(lambda a,b: a + b, ocs)

big_tree = TreeNeo(ocs)

print(big_tree)

<LocalTree Of Life | Root: LUCA - n.count: 374731- >

The resulting tree could be very large. In this case, the obtained tree (big_tree) comprises 374,731 occurrences. Remember that this tree is the resulting union of all the local taxonomic trees obtained from the neighbourhood of degree 4 around the cells where jaguars occurred.

### Selecting nodes from the Red List

We filter the “Species” nodes from the big_tree that are present in the Red List of threatened species. To do this we simply match the names using regular expressions. Using more sophisticated methods for data matching is out of the scope of the present example. We assume that the Red List data (a CSV file) have been loaded into a data frame with the name redlist.

## Filter critically endangered species

critical_sps = redlist[

(redlist.redlistCategory == 'Critically Endangered')

| (redlist.redlistCategory == 'Endangered')

| (redlist.redlistCategory == 'Vulnerable')

].scientificName.apply(str.lower)

protected_by_jaguar = map(lambda critical_sp:

filter(lambda sp: critical_sp in sp.name.lower(),

big_tree.species),

critical_sps)

## Remove empty lists

protected_by_jaguar = filter(lambda l:

l != [], protected_by_jaguar)

## flatten lists

threatened_species = reduce(lambda a,b: a + b ,protected_by_jaguar)

## remove species repetitions

threatened_species = list(set(threatened_species))

## Extract all corresponding occurrences and flatten list

t_ocs = reduce(lambda l1,l2: l1 + l2 ,

map(lambda l: l.occurrences, threatened_species))

## Instantiate new tree

threatened_tree = TreeNeo(t_ocs)

The threatened_tree is now a taxonomic tree that includes only the occurrences that match the species names of the Red List. To calculate the percentage of threatened species contained in the selected tree we can do the following:

## total number of critical endangered species

ncrit = len(critical_sps)

len(threatened_tree.species) / float(ncrit) * 100

13.49 %

That is, 13.49% of the threatened species are contained in the neighbouring regions where jaguars had been registered. To see whether this result is relevant, we calculate the percentage of the covered area with respect to the whole country. Before doing so, it is convenient to transform the selected geometries in a projected coordinate system with metric units.

#### Reprojecting data

The default CRS in the data used is in geographic coordinates with WGS84 datum (EPSG:4326). The units of this CRS are degrees; therefore, the calculated area is defined in squared degrees. To account for areas and distances in metres (or kilometres) we need to project the selected geometries into an appropriate projected coordinate system. To achieve this, we need to import some extra functions.

from shapely.ops import transform

from shapely import wkt,wkb

import pyproj

from functools import partial

Here we used the Alberts equal area conic projection to account for an accurate area representation. This projection is specified in a string using the Proj4 syntax.

projection_string = ''''''+proj=aea +lat_1=14.5 +lat_2=32.5 +lat_0=24

+lon_0=-105 +x_0=0 +y_0=0 +ellps=GRS80

+datum=NAD83 +units=m +no_defs;

''''''

mex_eq_area_proj = pyproj.Proj(projection_string)

## The WGS84 crs is defined as EPSG:4326

proj_in = pyproj.Proj(init='epsg:4326')

## function to project using the parameters of the

## original projection and the mexican equal area.

project = partial(

pyproj.transform,

proj_in,

mex_eq_area_proj)

## Transform all cells to calculate area.

projected_neighbours_cells = map(lambda cell:

transform(project,

cell.polygon_shapely),

neighbours)

To calculate the average cell size and the total area in square kilometers (1,000,000 m^2^) we do as follows:

tokm2 = 1000000 # to convert to sq. kilometers

areas = map(lambda cell: cell.area,

projected_neighbours_cells)

total_cell_area = sum(areas)

## calculate the mean

np.mean(areas) / tokm2

## standard deviation

np.std(areas)/ tokm2

The calculated average area of all cells is 27 ± 3 km^2^ and the total area is 8,509.81 km^2^.

### Trimming trees

In certain situations we need to select a particular branch of a tree. We can cut (trim) this branch by simply selecting a node and converting it into a TreeNeo instance to produce a full feature tree. The method (function) for converting a TreeNode into a full feature tree is plantTreeNode. We focus our attention on 4 branches of the threatened tree that co-occur with the presence of jaguars. These branches are mammals (class Mammalia), parrots (order Psittaciformes), amphibians (class Amphibia), and plants (kingdom Plantae).

#### Select the branch of interest

Trimming the tree is achieved by first selecting the nodes of interest and then converting all the descendant branches into fully featured trees. There is no restriction for selecting the taxonomic type of the node (mammals and amphibians are Class type while parrots are Order type).

mammals = threatened_tree.to_Animalia.to_Chordata.to_Mammalia

parrots = threatened_tree.to_Animalia.to_Chordata.to_Aves.to_Psittaciformes

amphibians = threatened_tree.to_Animalia.to_Chordata.to_Amphibia

plants = threatened_tree.to_Plantae

The method plantTreeNode() converts the TreeNode and resulting descendants into a full featured tree (TreeNeo object).

mammals = mammals.plantTreeNode()

birds = birds.plantTreeNode()

amphibians = amphibians.plantTreeNode()

plants = plants.plantTreeNode()

We can add all these trees together using the sum operation.

vertebrates = mammals + parrots + amphibians

However, as explained earlier, an optimized version for summing >2 trees is achieved by instantiating a TreeNeo with all the occurrences.

vertebrates = TreeNeo(mammals.occurrences +

parrots.occurrences +

amphibians.occurrences)

print(vertebrates)

The total number of occurrences contained in the vertebrates tree is:

<LocalTree Of Life | Root: LUCA - n.count: 2056- >

#### Ranking the most frequent nodes in the selected list of cells

We proceed now to rank some groups according to their frequency of occurrence within the cells of the study area (i.e., the jaguar’s neighbouring cells). The ranking analysis calculates this frequency for each node in a tree given a referential list of trees. That is, assuming that we have *n* different trees (e.g., 1 per cell) and a tree of interest (in this case threatened_tree), how frequently does each node appear in the global tree (e.g., threatened_trees) with respect to the list of *n* trees? Fig. 9 shows these frequencies visualized as the size of each node. In our implementation, this analysis is performed with the following method: countNodesFrequenciesOnList(list_of_trees). That is,

vertebrates.countNodesFrequenciesOnList(list_of_trees=sample_trees)

mammals.countNodesFrequenciesOnList(list_of_trees=sample_trees)

parrots.countNodesFrequenciesOnList(list_of_trees=sample_trees)

amphibians.countNodesFrequenciesOnList(list_of_trees=sample_trees)

plants.countNodesFrequenciesOnList(list_of_trees=sample_trees)

We can therefore rank by taxonomic level. In this example we show the procedure for family and species level in the different branches. Here, we show the corresponding top 5 nodes.

mammals.rankLevels()

mammals.families[:5]

[<LocalTree | Family: Muridae - n.count: 8 | AF: 0.30>,

<LocalTree | Family: Phyllostomidae - n.count: 8 | AF: 0.29>,

<LocalTree | Family: Cervidae - n.count: 14 | AF: 0.16>,

<LocalTree | Family: Heteromyidae - n.count: 3 | AF: 0.15>,

<LocalTree | Family: Tayassuidae - n.count: 158

| AF: 0.15>]

parrots.rankLevels()

parrots.species[:5]

[<LocalTree | Specie: Ara militaris (Linnaeus, 1766) - n.count: 27->,

<LocalTree | Specie: Amazona finschi (P. L. Sclater, 1864) - n.count: 23- >,

<LocalTree | Specie: Amazona auropalliata (Lesson, 1842) - n.count: 3- >,

<LocalTree | Specie: Amazona oratrix Ridgway, 1887 - n.count: 2- >,

amphibians.rankLevels()

amphibians.families[:3]

[<LocalTree | Family: Hylidae - n.count: 128- | AF: 0.083>,

<LocalTree | Family: Plethodontidae - n.count:

160 | AF: 0.05>,

<LocalTree | Family: Eleutherodactylidae -

n.count: 1- | AF: 0.016>]

plants.rankLevels()

plants.genera[:3]

[<LocalTree | Genus: Tillandsia - n.count: 3- | AF: 0.2>,

<LocalTree | Genus: Lonchocarpus - n.count: 5- | AF: 0.18>,

<LocalTree | Genus: Eugenia - n.count: 1- | AF: 0.15>]

### Associated raster (environmental) information

Here, we demonstrate how to access raster data associated with a taxonomic tree TreeNeo. The raster data used are related to environmental variables stored in the RGU. Currently there are 2 forms for accessing this information: (i) as a table with columns corresponding to environmental variables and rows defined by each occurrence (a point-based method) and (ii) as a raster object sampled from the associated geometry of each tree or, in general, any (multi) polygon object. The raster object features methods for visualization, geoprocessing, and data exchange.

#### Extracting raster information as table

To extract the data in this format use the method (function):

TreeNeo.associatedData.getEnvironmentalVariablesPoints()

The output is a Pandas dataframe with the associated values of climatic covariates. See the following example:

table = vertebrates.associatedData.getEnvironmentalVariablesPoints()

print(table[:1])

Here we only show the first record.

**Table 2:** Output for environmental variables

	MinTemperature	Precipitation	Vapor	SolarRadiation	WindSpeed
0	22.25	21.16	1.33	16,466.25	2.33

Here showing only mean values for some variables on a single record.

The geometric object of each tree is determined by the Occurrence nodes of the tree. In the graph database, each Occurrence node is linked to the Cell node that geographically contains the occurrence’s location. One of the attributes of the Cell object is the geographic polygon that defines its border. The union of all the corresponding Cell nodes is what determines the geometric feature of the tree TreeNeo. As such, the raster extraction process is performed on each of the tree’s associated cells.

#### Extracting raster objects from TreeNeo instances

To extract the associated raster object of a TreeNeo instance use the following method (function):

TreeNeo.associatedData.getAssociatedRasterAreaData([name of variable])

To obtain several environmental variables use associatedData.getEnvironmentalVariablesCells()

For example, information for a single variable can be obtained with

meantemp_data = vertebrates.associatedData.

getAssociatedRasterAreaData(

'MeanTemperature')

The raster object is automatically added to the TreeNeo object after the method is called. The raster objects are appended to the attribute associatedData.

### Extracting raster objects from arbitrary polygons

The extraction of raster objects is performed by the raster_api library, a Biospytial module for reading, writing, and processing raster objects using the RGU as back end.

The raster_api can use natively any object stored in the knowledge engine that has at least a 2D geometric feature (attribute). This includes the basic operations for querying, reading, and writing. For using external geometric objects such as Shapefiles, GeoPackages, or GeoJSON, the objects need to be transformed to their corresponding WKT or WKB (Well Known Binary) representation. Examples of these are described extensively in the Jupyter notebooks and in the documentation.

In this example we use the polygon defined by the border of Mexico to extract several raster objects (RasterData instances) using the raster_api module. We use these objects to compare the environmental ranges of the threatened species, the jaguars’ habitat, and the entire area of the country to conclude whether the environmental niches of the threated species are covered by the habitat of the jaguars and how these ranges are different with respect to the whole country.

#### Importing the polygon for Mexico

The first step in this is to import the polygon for Mexico. The default installation of Biospytial includes the WorldBorders dataset [100]. Assuming that this dataset is installed, we can import the polygon of Mexico with the API provided by the class Country located in sketches.models. Country is a vector dataset stored in the RDBMS. The geometric feature is stored as the geom column.

from sketches.models import Country

## The syntax follows the Django Query Set API

mexico = Country.objects.filter(name='Mexico').first()

mex_area = mexico.geom.area

## For reprojecting the area of Mexico we similarly do:

mex_shapely = wkt.loads(mexico.geom.wkt)

mex_projected= transform(project,mex_shapely)

To calculate the percentage of area covered by all the cells with respect with the total area of Mexico we can do

total_cell_area / mex_projected.area * 100

3.42%

**Figure 7:**
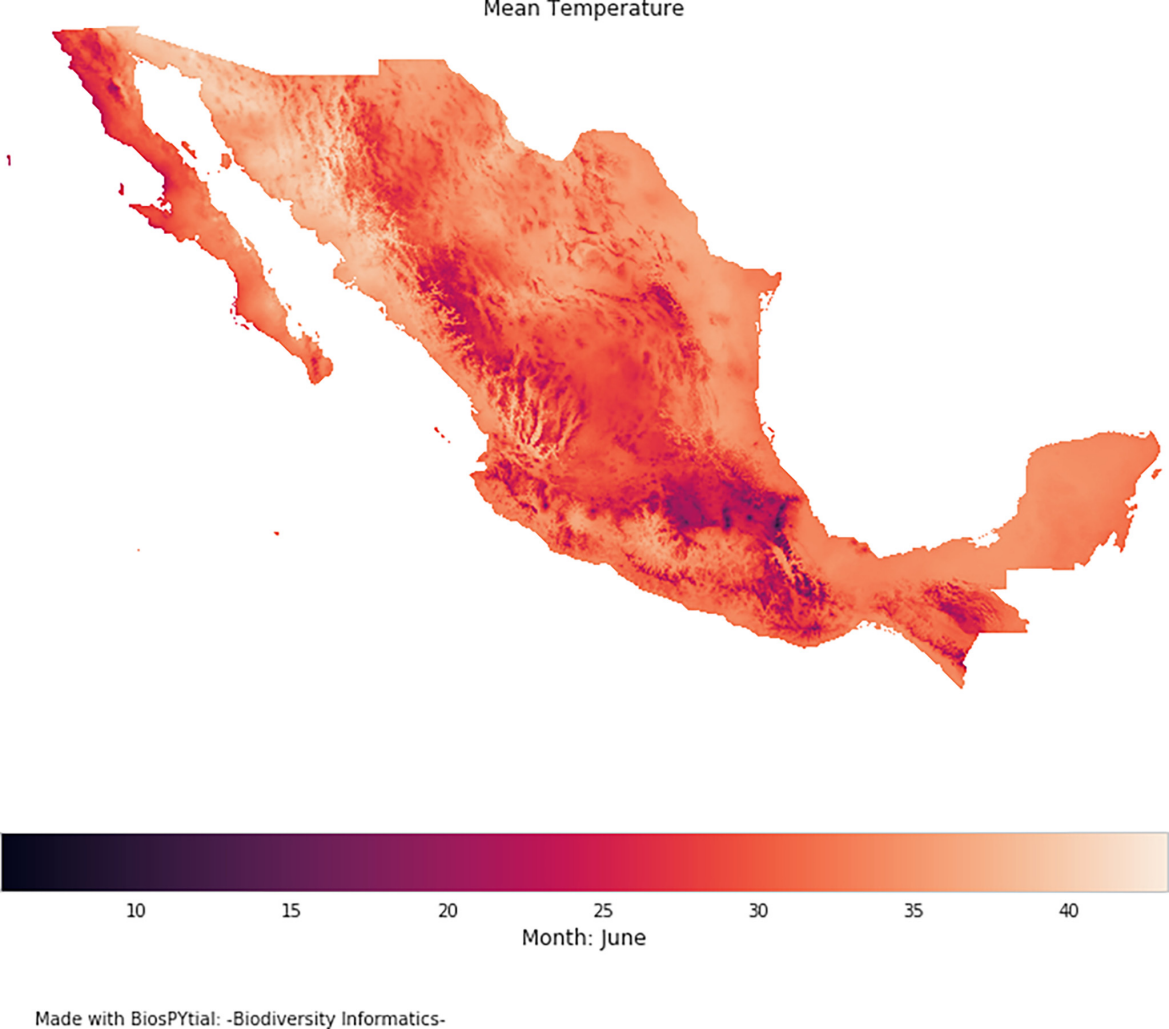
The output of the method display_field(), an easy way to visualize RasterData objects.

For example, we can display simple visualizations invoking the method display_field(). See Fig. 7.

vertebrates.associatedData.raster_MeanTemperature.display_field()

#### Interactive visualization

As an alternative, we can export the raster object as an xarray [101] instance for interactive visualization using the Geoviews [102] package. To export the associated raster data to an xarray object do the following:

meantemp = vertebrates.associatedData.raster_MeanTemperature.to_xarray()

**Figure 8:**
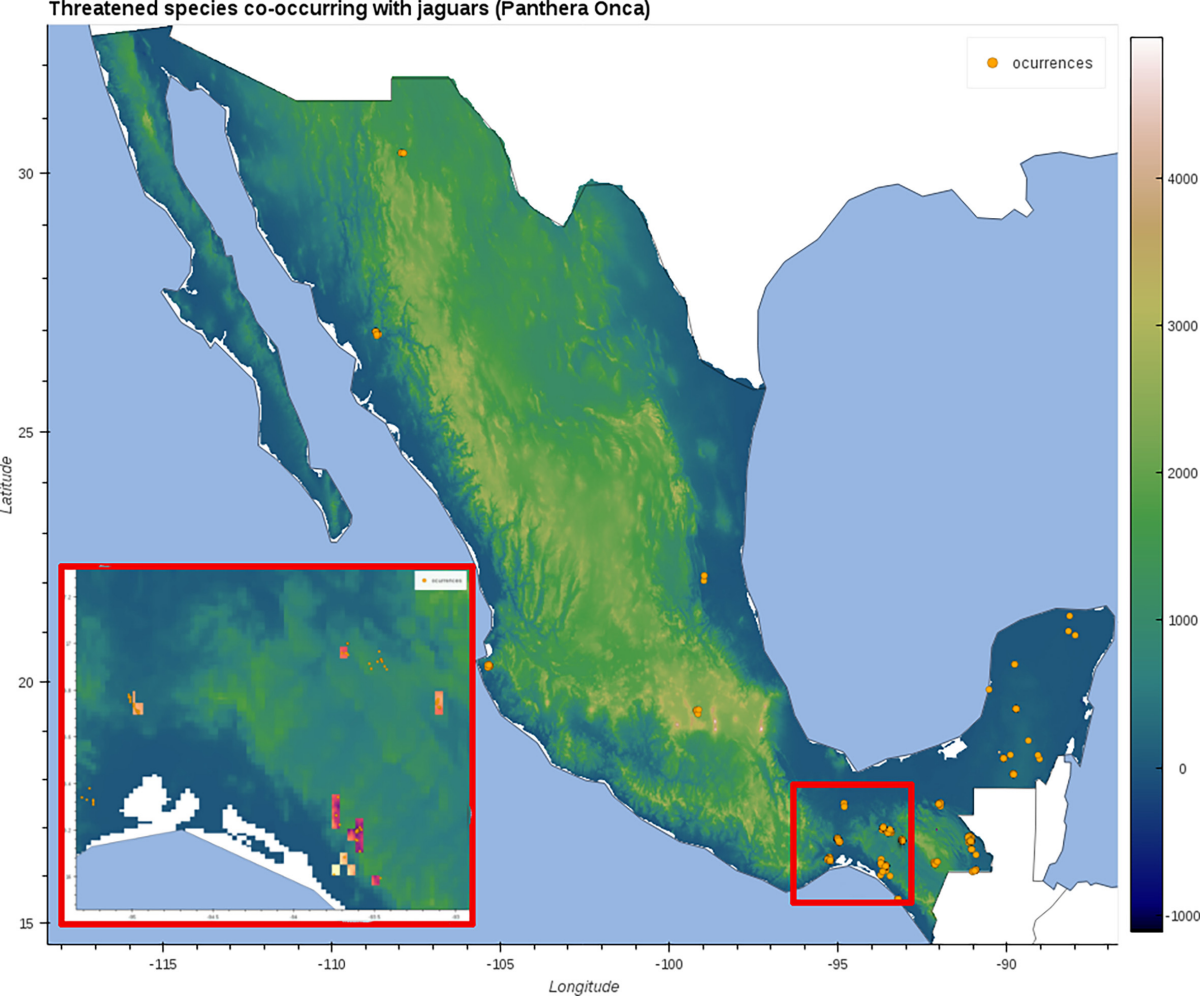
A composite figure showing 2 states of the interactive visualization. Orange dots represent occurrences of threated species associated with the presence of jaguars (*P. onca*). The inset shows the area inside the red square in the main map. The colored squares in the inset show the mean temperature associated with threatened vertebrates (phylum Chordata). The base map shows the elevation for all of Mexico. See section: “Data Used” for more information.

The following code gives an example of how to generate an interactive visualization using the vertebrates’ associated mean temperature data and the locations of the observed threatened species associated with the presence of jaguars. We used the elevation data for Mexico (extracted before) as base map. Fig. 8 shows this visualization at 2 different scales.

**Figure 9:**
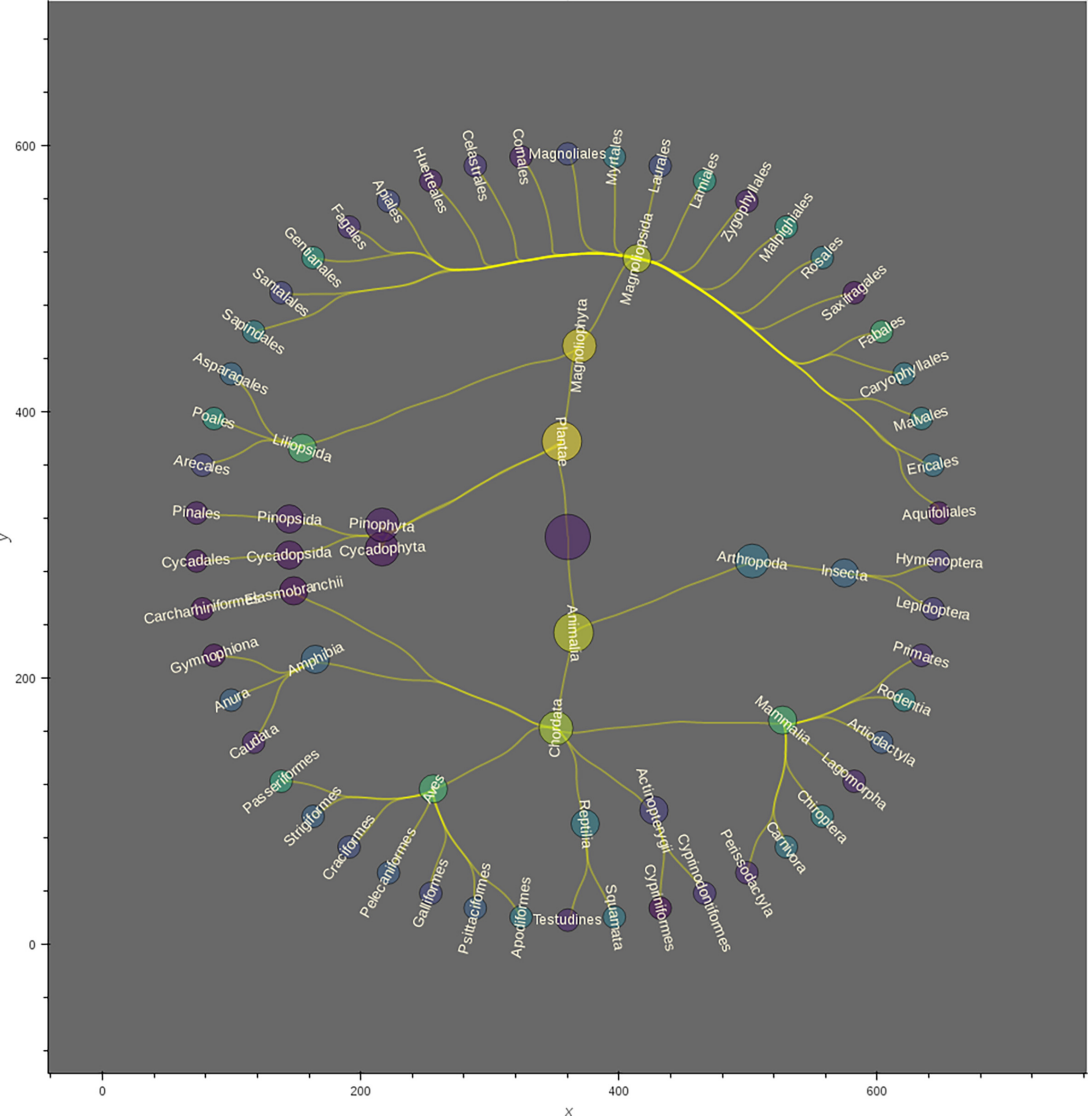
A tree visualization for the merged tree corresponding to threatened taxa, showing up to order level. The size of the nodes is proportional to the taxonomic level (the largest is the root of the tree, the smallest are orders). The node colouring indicates the frequency of occurrence with respect to all the neighbouring cells (neighbours of jaguars), ranging from brighter to darker for higher and lower ranked, respectively.

import geoviews as gv

from cartopy import crs

import geoviews.feature as gf

from geoviews import opts

gv.extension('bokeh')

sample_pt = gv.Points((env_threated_occurrences.x,env_threated_occurrences.y),

label='ocurrences').opts(

fill_color = 'orange',

line_color = 'black',

line_width = 0.5,

line_alpha = 0.4,

fill_alpha = 1.0,

size = 5,

)

elevation = all_mex_datasets[0].to_xarray()

elevds = gv.Dataset(elevation,crs=crs.PlateCarree())

elevimg = gvds.to(gv.Image,['Longitude','Latitude']

).opts(cmap=plt.cm.gist_earth)

temp = meantemp.where(((meantemp.Longitude > -95) &

(meantemp.Longitude < -89) &

(meantemp.Latitude > 15) &

(meantemp.Latitude < 19)),

drop=True)

temp.name = meantemp.name

tempds = gv.Dataset(temp,crs=crs.PlateCarree())

tempimg = tempds.to(gv.Image,['Longitude','Latitude']).opts(cmap=plt.cm.magma)

## Display the map

map_ = (elevimg * gf.ocean * gf.coastline * gf.borders * tempimg * sample_pt )

### Network visualization and analysis

Each tree instance induces an acyclic graph. We can convert the tree into a networkx object to visualize and analyse its network properties. To do this, we simply need to use the method tree.toNetworkx(depth_level=[k]), where *k* is the taxonomic level to reach in the tree, 0 for root 7 for species level.

#### Visualization

A method for interactive visualization has been developed using the Holoviews [103] framework. To do this we need to invoke the following method:

## Plot the Tree

from drivers.tools import to_interactivePlot

network = to_interactivePlot(threatened_tree,label_depth=8)

The output is a dictionary with 2 key items: 1 for labels and the other for the actual graph (nodes and edges). To plot the whole graph we need to overlay both items.

network['labels'] * network['graph']

#### Analysis with standard graph algorithms

The TreeNeo structures are particular cases of graph traversals. As such, they can be analysed with graph theoretic methods. The library NetworkX [104] is a Python package designed for analysing the structure, dynamics, and functions of complex networks. It includes standard graph algorithms and analysis measures as well as tools for import and export to other standard formats. We can convert a TreeNeo using the method toNetworkx(depth_level ), where depth_level is the depth of the graph to be generated. In the next example we convert the threatened_tree to a NetworkX object and use this to calculate its corresponding adjacency matrix.

threatened_graph = threatened_tree.toNetworkx(depth_level=7)

from networkx import adjacency_matrix

M = adjacency_matrix(threatened_graph)

# uncomment this to plot the matrix

#plt.imshow(M.todense())

Representing TreeNeo objects into NetworkX graphs brings new possibilities for analysis and modelling. We hope this example will awaken the spirit of the reader to explore the potential of representing data as complex graph structures.

## Conclusions

Biospytial uses open source standards to integrate geospatial ecological Big Data as a tool for ecological niche modelling and the analysis of species distributions. This integration creates a complex network of data with enormous potential for data mining, information retrieval, and visualization. At the core, a web of semantic-wise relationships constitutes a corpus of taxonomic and environmental knowledge that opens up new ways to query and unveil complex ecological relations. To our knowledge, there is no other open source system with the design and capacity to achieve this including (i) storing information in a hybrid relational-graph system and (ii) performing geospatial processes in vector and raster scalable databases.

A practical example provided a glimpse into how to query and manipulate taxonomic tree structures, as well as how to extract data, conduct frequency analysi,s and visualize results. The example demonstrated a new procedure to rank co-occurring taxonomic groups in an arbitrary size neighbourhood of pixels.

The GBIF occurrence data include information only on location and taxonomy, and in this sense the data are limited. However, the engine’s design allows the capture, extension, and exploration of a semantic interpretation of the data by adding other types of relations. For example, linking information on trophic networks to the taxonomic backbone can help in analysing spatial patterns of trophic groups and dependant species, a key question in conservation biology.

The development of Biospytial has followed best practices in scientific programming [105]. We recognize that spatial analyses are often not generalizable and therefore replicable. However replicability and reproducibility can be enhanced by increasing openness and documentation transparency and completeness [106–108]. In fact, Biospytial’s source code is open and can be accessed at [109] while this article is Open Access. In the future, Biospytial can be further developed into a system not only for integration and distribution of datasets but also as a tool for collaboration, experimentation, validation, and reproduction of results in the era of Open Science, satisfying also the requisites of second-generation SDI.

## Availability of Supporting Source Code and Requirements

- Project name: Biospytial
- Project home page: https://github.com/molgor/biospytial
- Operating System(s): Platform independent (not tested in Windows)
- Other requirements: Docker 1.13 or higher
- License: GNU General Public License version 3.0 (GPLv3)
- Memory requirements: 40GB in HD for installing the database and ≥16 GB in RAM for running the example.
- RRID:SCR_018226
- biotools:biospytial

The current example is located inside the folder examples with the name [Official Demo] Co-occurrences_Jaguar.ipynb. The example has been modified only in the neighbourhood order, changing from 4 to 1. This modification reduces the data to process and the executing time.

## Availability of Supporting Data and Materials

Snapshots of our code and other supporting data are openly available in the *GigaScience* repository, GigaDB [110]. The container images can be downloaded automatically using the script installEngine.sh. Instructions for installing and running the engine are located in the project’s home page.

**Table 3:** Corresponding URLs for source code and container images for the Biospytial engine.

Module name	URL
Graph Storage and Processing Unit	https://hub.docker.com/r/molgor/postgis_biospytial
Biospytial Computing Engine	https://hub.docker.com/r/molgor/biospytial
Relational Geoprocessing Unit	https://hub.docker.com/r/molgor/neo4j_biospytial
Source code	https://github.com/molgor/biospytial
Data	http://dx.doi.org/10.5524/100723

The modules and the source code do not include data. These should be installed separately or loaded independently.

## Additional Files

“Jupyter notebook for the tutorial section”: [Official Demo]Co-ocurrences_Jaguar.ipynb
“Supplementary materials I”: Adding data in Biospytial (pdf file)
“Supplementary materials II”: Mathematical formalisms (pdf file)

## Abbreviations

ACID: atomicity, consistency, isolation, durability; API: application programming interface; BCE: Biospytial Computing Engine; BLOB: binary large object; CONABIO: National Commission for the Knowledge and Use of Biodiversity; CRS: coordinate reference system; CSV: comma separated value; DAG: directed acyclic graph; DEM: digital elevation model; EBV: essential biodiversity variable; EPSG: European Petroleum Survey Group; ESA: European Space Agency; GBIF: Global Biodiversity Information Facility; GDAL: Geospatial Data Abstraction Software Library; GIS: Geographic Information Systems; GSPU: Graph Storage and Processing Unit; MPI: Message Passing Interface; NASA: National Aeronautics and Space Administration; OGM: object-graph mapping; ORM: object-relational mapping; RDBMS: Relational Database Management System; RGU: Relational Geoprocessing Unit; SDI: spatial data infrastructure; ToL: Tree of Life; WKB: Well Known Binary; WKT: Well Known Text.

## Competing Interests

The authors declare that they have no competing interests.

## Funding

J.M.E.M and the project were jointly sponsored by the Doctoral Scholarships Program from the Mexican Science and Technology Council (CONACYT, Becas al Extranjero), the Faculty of Science and Technology from Lancaster University (FST-LU) and the GBIF Consortium through the GBIF Young Researchers Award (2016). L.S and P.M.A are supported by the Engineering and Physical Sciences Research Council grant number EP/R01860X/1 “Data Science of the Natural Environment”

## Authors’ Contributions

J.M.E.M. and P.M.A. conceived the original idea, which was further refined by all authors. The semantic structures and graph traversals were designed by J.M.E.M. with the mentorship of L.S. for integrating datasets. The software and system’s design was developed by J.M.E.M. under the supervison of P.M.A. and L.S. The writing of the original draft was done by J.M.E.M. with reviewing and editing from P.M.A. and L.S.

## Supplementary Material

giaa039_GIGA-D-19-00265_Original_Submission

giaa039_GIGA-D-19-00265_Revision_1

giaa039_GIGA-D-19-00265_Revision_2

giaa039_Response_to_Reviewer_Comments_Original_Submission

giaa039_Response_to_Reviewer_Comments_Revision_1

giaa039_Reviewer_1_Report_Original_Submission Dawn Wright -- 8/4/2019 Reviewed

giaa039_Reviewer_2_Report_Original_Submission Jinfeng Wang -- 9/18/2019 Reviewed

giaa039_Reviewer_3_Report_Original_Submission Marc Macias-Fauria -- 10/6/2019 Reviewed

giaa039_Supplemental_Files
