# Supplementary material for: Biospytial: spatial graph-based computing for ecological Big Data
Source: Gigascience. 2020 May 11;9(5):giaa039. doi: 10.1093/gigascience/giaa039 (PMC7213554; doi:10.1093/gigascience/giaa039)
Supplement: giaa039_GIGA-D-19-00265_Revision_1 [file giaa039_giga-d-19-00265_revision_1.pdf]

GigaScience

Biospytial: spatial graph-based computing engine for ecological big data

--Manuscript Draft--

**Manuscript Number:**	GIGA-D-19-00265R1
**Full Title:**	Biospytial: spatial graph-based computing engine for ecological big data
**Article Type:**	Technical Note
**Funding Information:**	Consejo Nacional de Ciencia y Tecnología (Becas al Extranjero) — Mr. Juan Escamilla Molgora<br>Lancaster University (Faculty of Science and Technology) — Mr. Juan Escamilla Molgora<br>Global Biodiversity Information Facility (GBIF Young Researchers Award 2016) — Mr. Juan Escamilla Molgora
**Abstract:**	Biospytial is a modular open source knowledge engine designed to import, organise, analyse and visualise big spatial ecological datasets using the power of graph theory. Specifically, it handles species occurrences and their taxonomic classification for performing ecological analysis on biodiversity and species distributions. The engine uses a hybrid graph-relational approach to store and access information. The data are linked with relationships that are stored in a graph database, while tabular and geospatial (vector and raster) data are stored in a relational database management system (RDBMS). The graph data structure provides a scalable design that eases the problem of merging datasets from different sources. The linkage relationships use semantic structures (objects and predicates) to answer scientific questions represented as complex data structures stored in the graph database. In this sense, we used species occurrences, taxonomic classification, and climatic datasets to build a knowledge graph of the Tree of Life embedded in an environmental and geographical grid. Biospytial comprises three interconnected components: i) a Geospatial Processing unit (GPU) supported by a RDBMS with geoprocessing capabilities, ii) a Graph Storage and Querying Unit, and iii) a graph-relational package, called: The Biospytial Computing Engine (BCE) that integrates all the system's components. It also includes tools like: interactive notebooks (Jupyter), graph analytic libraries (NetworkX) and statistical frameworks (PyMC3). The Biospytial approach reduces the complexity of joining datasets using multiple primary-foreign key relations, a drawback in RDBMS. Applied to ecological data, it allows the discovery and inference of relationships using the interconnected network of taxonomic and spatial relationships. Its modular and scalable design makes it possible to run and distribute several instances simultaneously, allowing fast and efficient handling of big and complex ecological datasets. An example applied to the conservation of threatened species from the IUCN Red List using the co-occurrence of jaguars (Panthera onca) is included. This example demonstrates the engine's capabilities in performing basic taxonomic trees manipulation,analysis and visualization of taxonomic groups co-occurring in space.
**Corresponding Author:**	Juan Escamilla Molgora<br>Lancaster University<br>Lancaster, Lancashire UNITED KINGDOM
**Corresponding Author Secondary Information:**	
**Corresponding Author's Institution:**	Lancaster University
**Corresponding Author's Secondary Institution:**	
**First Author:**	Juan Escamilla Molgora
**First Author Secondary Information:**	
**Order of Authors:**	Juan Escamilla Molgora<br>Peter Atkinson<br>Luigi Sedda

Powered by Editorial Manager® and ProduXion Manager® from Aries Systems Corporation

Order of Authors Secondary Information:	
Response to Reviewers:	Dear Dr Nicole Nogoy,<br>Editor of GigaScience and reviewers<br><br><br>Object: Manuscript GIGA-D-19-00265 “Biospytial: spatial graph-based computing engine for ecological big data”.<br><br>We are grateful to the reviewers for their comments which helped in improving the quality of the manuscript. We also thank the Editor for giving us the opportunity to revise the<br>manuscript. We apologize for the delayed response and we thank you and the reviewers for<br>your patience during this long process also.<br>We took on board all the comments as shown in the point to point reply to reviewers starting in the next page. The software is publicly available via github, and the data will be<br>upload in SciCrunch shortly.<br><br>We hope these corrections satisfactorily address the various comments made by the Reviewers. Please let us know if any further corrections are required.<br><br>Yours sincerely,<br><br><br>Juan Escamilla Molgora on behalf of all the authors.<br><br># Responses to the reviewers<br>---<br>Notes:<br>Paragraphs that start with >> are our responses.<br>The format of the text is in Markdown and a PDF version of this text is included in this submission.<br><br>The new additions have been highlighted in the updated manuscript.<br><br>--<br># Reviewer 1<br><br>I very much enjoyed reading this paper and as a geospatial data scientist/GIScientist am very happy to see this considered for GigaScience.<br>I found the paper to be extremely comprehensive, very well-written, sound it computational approach, and reflecting a good knowledge of ecoinformatics that supports the global ecological community. The system architecture described is quite exciting. I admit to a lower level of expertise where knowledge engines are concerned, but know of many colleagues in the geospatial community who will be excited to see this new engine for **ecological data** that incorporates semantic relations and integrates into the geospatial semantic web.<br>Unfortunately these colleagues do not read GigaScience, but I will guide them appropriately. :-)<br><br>>> We thank the reviewer for the very positive general comment on our paper. We do hope to attract new readers to GigaScience.<br><br>## Elaborate more discussions around the SDI I found it interesting that the authors chose "spatial data infrastructure" as their first key word. I think<br>I know where they are heading with this, and this is an important connection to make, but might they consider adding a big more to the introduction or concluding sections of the paper to make a stronger connection to the traditional "SDI" community? For instance, I am wondering how their engine qualifies as an SDI in the more traditional sense of the term. A traditional SDI implements a broader framework of geographic data, metadata, standards, institutional arrangements, policies, and tools that are interactively connected in order to make the use of spatial data more efficient and flexible.

European INSPIRE is a shining example of this, as well as the United Nation's SDI, and the Convention on Biological Diversity. Could the authors briefly add how Biospytial is aiding in SDI beyond just employing GBIF or IUCN Red List data as a use case?

>>We fully agree with this vision of SDI and we thank the reviewer for raising this valid point. We have contextualised our engine in the light of current SDI definition. See lines 151-156.

By the way, how in the world does one properly pronounce "biospytial" (long y or short y). Could the authors provide a hint, if nothing else, just in their response to me as a reviewer. :-) In my mind I kept wanting to say "biospatial" with an "a!"

>>This comment is very useful, we have explained why we called the engine biospytial and how to pronunce it. See lines: 148 and 149.

Another very minor observation: the authors identify their GPU (Geospatial Processing Unit). So many of us are used to understanding GPU in the already established Graphics Processing Unit parlance of computer hardware terminology.

>>We have changed to RGU (relational geoprocessing unit) instead of using GPU.

More importantly, a major strength of this research is the interweaving of so many open source/open science technologies. I love Table 1 (a veritable "who's who"). And I applaud the use of containerization. I couldn't agree more with the statement on Line 172 that the idea here is to move the processes around, NOT the big data. I would add that the idea behind geospatial cloud computing, writ large, is to move the spatial analyses TO the data, rather than downloading or moving big data sets around. I mention this for discussion sake, not necessarily as a required change of wording in the paper.

>> We fully agree with this comment, and we extend our sentence in line 191-193 in order to stress the importance of performing the analyses where the data is located. See new line aaa.

What I *do* further suggest for minor changes have to do with the authors' important mention of reproducibility and replicability. Reproducibility is first mentioned on Line 17, and if I am understanding the authors' intent, I would like to suggest a few more references that they might consider consulting and adding:

* Barba LA. 2018. Praxis of reproducible computational science. Authorea: doi: 10.22541/au.153922477.77361922. doi:10.22541/au.153922477.77361922.
* Jasny BR, Wigginton N, McNutt M, Bubela T, Buck S, et al. 2017. Fostering reproducibility in industry-academia research. Science 357(6353): 759.
* Teytelman L. 2018. No more excuses for non-reproducible methods. Nature 560: 411. doi: 10.1038/d41586-018-06008-w.
* Shannon J, Walker K. 2018. Opening GIScience: A process-based approach. International Journal of Geographical Information Science 32(10): 1911-1926, doi: 10.1080/13658816.2018.1464167.

On Lines 170 and 224 they talk about *replicating* their applications and/or analysis. Do they mean reproduce instead? In the literature there is now an important distinction between reproducibility (a condition where results or products can be continually reproduced using the same data and methods) and replicability (a higher level of scientific rigor where results or products can be reproduced using different samples of data and different software). In addition to the references above a good primer is now at https://sgsup.asu.edu/sites/default/files/rr_workshop_sparc_summary.pdf, as part of a recent workshop at https://sgsup.asu.edu/sparc/RRWorkshop. This part is a digression, just for discussion only: I think we can all acknowledge that a "reproducibility crisis" has received widespread attention across the sciences, but perhaps nowhere as much as in psychology, where numerous attempts

Powered by Editorial Manager® and ProduXion Manager® from Aries Systems Corporation

to reproduce previous findings have failed. It can be argued that scientists generally lack the relevant skills and tools to ensure that their findings are reproducible and replicable, and that much academic literature amounts to little more than advertising of findings, rather than detailed reporting that would allow results to be reproduced and replicated. "Show me" should be more important than "trust me" in the culture of science. Efforts to build a culture of open science, in which data, tools, methods, and software are all made accessible to everyone, are welcome. But openness in and of itself is not sufficient to ensure that results can be reproduced, let alone replicated.

>>We fully agree with these comments and we thank the reviewer for sending us the references and workshop links. They were very useful and we have added a paragraph in the conclusions recognising the limitations in reproducibility and replicability in spatial analyses. We also added the Barba 2019, Teytelman and Shannon references. See lines 733 - 740.

Detected typos Lines 85-86 - the full definition of the GEO BON acronym is Group on Earth Observations Biodiversity Observation Network (they are missing the BON part)

>>Done.

Line 92 - I may have missed it, but can the authors please expand the PREDICTS acronym?

>>Done.

Congratulations again to the authors for this fine work and best wishes to them for continued success.

>> Thanks for your words we are really pleased with your comments and advices.

Reviewer 2

1.What classification algorithms are used to construct the tree?

>>The taxonomic classification mentioned in various points in the manuscript, is based on the classical natural systematic classification of the species in the tree of life. Therefore this classification already exists and it is used here to organize the taxa in a hierarchical structure. To remove any confusion we added a reference of the updated taxonomic classification used here.

2.Spatial stratified heterogeneity (SSH) becomes a serious problem when data is big and diverse. A sample is biased to SSH population when the sample don't cover all strata; and statistics become confounded when they are applied globally to SSH population. Therefore, SSH should be tested at early stage of big spatial data analysis. IF SSH is insignificant, a global model is safe; otherwise, a simple solution is to apply a model in strata, separately.

>>We thank the reviewer for this very good point. We have added a reference (Wang et al 2016) in line 87 recognising the important role of stratification to reduce bias in spatial analyses.

3.To illustrate the robust of the tool, the authors may provide several different examples for readers to practice. For example, cities evolution tree, besides the tree in the paper.

>>We understand the importance of robustness of our proposed engine. We have shown that any spatial data with an existing structure can be employed in our

Powered by Editorial Manager® and ProduXion Manager® from Aries Systems Corporation

engine. We believe that adding other examples is out of the scope. The algorithm is open and we hope that others will build up the portfolio of applications. However, we have added additional components to the jaguar example to fully show its aplicability.

4.To increase tool's users who are unfamiliar with computer language, draw a flowchart so a user can follow as he/she is doing in the real world.

>>A full updated working example is provided towards the end of the paper.

Review 3

Overall This manuscript presents a knowledge engine designed to manage large spatial ecological data in a variety of formats and in an efficient way, using graph theory to maximise this efficiency, and enabling a series of operations. The manuscript is essentially composed of two sections, one in which the engine is described, and another one where its potential and applicability are shown in the form of meaningful examples.The paper is well written and structured, and presents a tool that can be of great use to ecologists and natural scientists, as well as to conservation managers with a natural sciences background. I suggest only some minor revisions, several of them asking for clarifications.

The jaguar example ends somewhat abruptly. In order to make a stronger point in showing the potential of the presented knowledge engine, it would be good if the authors would add a paragraph rounding up the results obtained in the exercise e.g. the reader ends up not being presented with the taxa most associated with the jaguar, or if these taxa were expected or else they are surprising. Linked to this, it would be interesting to follow up with the potential of the environmental layers to describe if the areas with jaguar are exceptional or not climatically, or topographically, or both, within Mexico. In other words, your example ends too quickly and more could be shown of it towards the end that would increase the perception of the reader regarding the potential and usefulness of the engine you present.

>> We have added a new section in the manuscript (section 4.3) describing the taxa and environmental results for jaguars.

Linked to the above, you could be more creative with figure 6, and figure 7 is nice but too messy. Consider showing a subset, and discuss it more in the text.

>>Both figures have been modified. Figure 8 (formerly 6) shows the elevation map (DEM) as base map displaying occurrences as points and environmental raster data objects as small overlapping regions. In figure 9 (formerly 7) we reduced the tree to only include orders, classes, phyla and kingdoms which reduces the number of nodes drastically. We improved the readability by assigning same size for all nodes' labels. We changed the color of the nodes representing the frequency (abundance) of taxa.

In page 24 and after, the codes include the term 'lambda' in many lines, and no explanation is given as to what that means. Could you specify what it means? Is it an anonymous function? In any case, this paper will be read by biologists with no background on computing science and the terms should be specified clearly.

>>Full explanation of the lambda functions and the joint effect of the map-lambda expression was added. See lines 506 - 513 and lines: 519 - 522.

In page 32, the total area of the cells is computed. It is not clear what is the original area of each cell.

Powered by Editorial Manager® and ProduXion Manager® from Aries Systems Corporation

>> We did the following amendments (lines: 599 - 608):
* Added subsection for reprojecting to conic equal area for measuring areas in meters.
* Added subsection for importing polygon from Mexico with reprojection.
* Added total area calculation and average size for each cell.

The GBIF database is composed of points (coordinates). How is this translated to a cell of a given area? Are you using the 1' DEM or the 1km environmental layers? In any case, GBIF coordinates can be of varying reliability, and a buffer is normally advised. It would be good to know where the cell area comes from (it was not too clear in the manuscript as it stands), and whether the coordinates in GBIF are taken as precise points. See this as a reference: https://onlinelibrary.wiley.com/doi/full/10.1111/ele.12624

>> We use the precise location (lat, lon wgs84 coordinates) of the GBIF occurrences given by their GBIF API / data (line 387). The occurrences are aggregated according to their taxonomy given that each occurrence belongs to a certain species. Although this was explained in lines: 403 - 407, we acknowledge that it was not clear enough. We added a more comprehensive explanation of the process for generating the local taxonomic trees on lines: 408 to 418. In addition, the procedure for aggregating occurrences through all the different taxonomic levels is described in supplementary materials II.

>> We thank the reviewer for pointing out the lack of clarity in the used grid. We included a brief description on how the grid system is created with a reference to the functions that generate customized grids (Lines: 397-399). In addition, we included a more explicit description of the grid used in 'worked example' under a new section named: 'Additional data used' (lines: 436-440).

>> We agree on the importance of estimating multidimensional biases, gaps and uncertainties in opportunistic samplings and citizen science records such as GBIF. For this reason we decided to use the complete information of location (point coordinates) of every record. However, accounting for these problems in the current worked example is out of the scope of the engine at this moment. We are, however, optimistic that the engine will help identify better this limitations of the data with the use of automatic or semi automatic procedures applied to large volumes of occurrences. Nevertheless, we thank the reviewer for the suggested reference as this is an issue that hopefully could be tackled in further applications of the engine.

The paragraph starting in line 511 contains a conclusion that is very difficult to sustain, since it is based on the assumption that threatened species are evenly distributed across the country. We know that this is not the case. I would be less categorical with it (i.e. it would seem that jaguars occur in places where other threatened species tend to cluster). The 'five times more likely' is not believable given the assumption.

>> Agreed, the paragraph is misleading or meaningless with the presented assumption. It has been removed.

In general, the use of numbers for references is fine, but in some instances, it is strange: e.g. in cases where you refer to a citation in the form "…[20] proposed that…", it would help the readability to add "Smith et al. [20] proposed that…"

>> The numbering system for references have been changed to author names plus year.

	### Detected typos<br>>> All suggestions were covered. We thank the reviewer for her/his time and positive feedback.
**Additional Information:**	
**Question**	**Response**
Are you submitting this manuscript to a special series or article collection?	No
**Experimental design and statistics**<br><br>Full details of the experimental design and statistical methods used should be given in the Methods section, as detailed in our Minimum Standards Reporting Checklist. Information essential to interpreting the data presented should be made available in the figure legends.<br><br>Have you included all the information requested in your manuscript?	No
If not, please give reasons for any omissions below.<br><br>as follow-up to "**Experimental design and statistics**<br><br>Full details of the experimental design and statistical methods used should be given in the Methods section, as detailed in our Minimum Standards Reporting Checklist. Information essential to interpreting the data presented should be made available in the figure legends.<br><br>Have you included all the information requested in your manuscript?<br><br>"	The manuscript describes a software for data management and analysis. It does not use or describe any statistical analysis or experimental design.
**Resources**<br><br>A description of all resources used, including antibodies, cell lines, animals and software tools, with enough information to allow them to be uniquely	Yes

identified, should be included in the Methods section. Authors are strongly encouraged to cite Research Resource Identifiers (RRIDs) for antibodies, model organisms and tools, where possible.<br><br>Have you included the information requested as detailed in our Minimum Standards Reporting Checklist?	
**Availability of data and materials**<br><br>All datasets and code on which the conclusions of the paper rely must be either included in your submission or deposited in publicly available repositories (where available and ethically appropriate), referencing such data using a unique identifier in the references and in the “Availability of Data and Materials” section of your manuscript.<br><br>Have you have met the above requirement as detailed in our Minimum Standards Reporting Checklist?	Yes

Powered by Editorial Manager® and ProduXion Manager® from Aries Systems Corporation

Manuscript (revised) Click here to access/download;Manuscript;paperBiospytial-2revision.pdf

Biospytial: spatial graph-based computing engine for ecological big data

Juan M. Escamilla Molgora[a,b,1,*], Luigi Sedda[b,2], Peter M. Atkinson[c,3]

[a] *Lancaster Environment Center, Lancaster University, Lancaster LA14YQ, UK*
[b] *Centre for Health Informatics, Computing and Statistics (CHICAS), Lancaster Medical School, Faculty of Health and Medicine, Lancaster University, Lancaster LA1 4YQ, UK*
[c] *Faculty of Science and Technology, Lancaster University, Lancaster LA1 4YR, UK*
[d] *Lancaster Medical School, Faculty of Health and Medicine, Lancaster University, Lancaster LA1 4YQ, UK*

Abstract

Biospytial is a modular open source knowledge engine designed to import, organise, analyse and visualise big spatial ecological datasets using the power of graph theory. Specifically, it handles species occurrences and their taxonomic classification for performing ecological analysis on biodiversity and species distributions. The engine uses a hybrid graph-relational approach to store and access information. The data are linked with relationships that are stored in a graph database, while tabular and geospatial (vector and raster) data are stored in a relational database management system (RDBMS). The graph data structure provides a scalable design that eases the problem of merging datasets from different sources. The linkage relationships use semantic structures (objects and predicates) to answer scientific questions represented as complex data structures stored in the graph database. In this sense, we used species occurrences, taxonomic classification, and climatic datasets to build a *knowledge graph* of the Tree of Life embedded in an environmental and geographical grid. Biospytial comprises three interconnected components: *i)* a relational geoprocessing unit (RGU) supported by a RDBMS with geospatial capabilities, *ii)* a Graph Storage and Querying Unit, and *iii)* a graph-relational package, called: *The Biospytial Computing Engine (BCE)* that integrates all the system's components. It also includes tools like: interactive notebooks (Jupyter), graph analytic libraries (NetworkX) and statistical frameworks (PyMC3). The Biospytial approach reduces the complexity of joining datasets using multiple *primary-foreign* key relations, a drawback in RDBMS. Applied to ecological data, it allows the discovery and inference of relationships using the interconnected network of taxonomic and spatial relationships. Its modular and scalable design makes it possible to run and distribute several instances simultaneously, allowing fast and efficient handling of big and complex ecological datasets. An example applied to the conservation of threatened species from the IUCN Red List using the co-occurrence of jaguars (*Panthera onca*) is included. This example demonstrates the engine's capabilities in performing basic taxonomic trees manipulation,analysis and visualization of taxonomic groups co-occurring in space.

Keywords: spatial data infrastructure, biodiversity informatics, ecological knowledge engine, big ecological data, open science

*Corresponding author
Email addresses: j.escamillamolgora@lancaster.ac.uk (Juan M. Escamilla Molgora), l.sedda@lancaster.ac.uk (Luigi Sedda), pma@lancaster.ac.uk (Peter M. Atkinson)
[1]https://orcid.org/0000-0002-3682-9828
[2]https://orcid.org/0000-0002-9271-6596
[3]https://orcid.org/0000-0002-5489-6880

1. Introduction

The IT revolution has created the opportunity to compute, store and transfer massive amounts of information. It is estimated that the volume of all digital information will surpass 175 Zettabytes (ZB) (1 ZB = 10^{21} bytes) by 2020 (Reinsel et al., 2018). In addition, the growth in data follows an exponential curve that doubles in volume every two years ((Kurzweil, 2004; Hilbert and López, 2011; Gantz and Reinsel, 2011)). Moreover, this expansion in data production has occurred in all human activities, including the environmental sciences. Novel approaches for measuring natural processes are being applied, adding more reliable and diverse data, and environmental measurements cover a wide range of spatial and temporal scales ranging, for example, from long-term ecological experimental plots (Weigelt et al., 2010; Borer et al., 2014) to near-real time imagery from Earth observation satellites systems like NASA's *Joint Polar Satellite System* (National Aeronautics and Space Administration et al., 2020) and ESA's *Copernicus* programme (European Space Agency, 2014). This IT era is opening new opportunities for greater understanding of nature. For example, pervasive Internet connectivity has made possible the transfer of data across large distances in a short time; and the multifunctional capabilities of mobile and *smart* devices has enabled the management and deployment of collaborative surveys at low marginal costs. Geospatial sciences have benefited in particular. Methodologies for collecting, annotating and curating these new sources of spatial data have been proposed by (Goodchild, 2007; Heipke, 2010; Kamel Boulos et al., 2011) under the term *citizen-science*; where data are collectively assembled by a community of enthusiasts and volunteers. Some iconic examples of these (*crowd-based*) platforms are OpenStreetMap (OpenStreetMap Contributors, 2019) for geographic maps and the *Global Biodiversity Information Facility* (GBIF), an international consortium of research and governmental institutions that gathers and publishes information of all types of biodiversity occurrences (GBIF Secretariat, 2015).

The exponential growth of data imposes new challenges for storage, access, integration and analysis. In recent years, new theoretical methods and technologies are being developed to tackle

these problems. The name *Big Data* is now an umbrella term for methods dealing with huge, complex, and heterogeneous datasets that cannot be handled with traditional methods. See (Chen et al., 2014) and (Mikalef et al., 2018) for a review of the field and (Li et al., 2016) for theoretical and practical challenges involving big geospatial data.

A fundamental goal in ecology is the understanding of the relationships between living beings and the environment. A requirement to achieve this goal is the integration of independent studies and measurements to validate hypotheses on potential causal relations. To test the existence of these causalities, a substantial number of inputs in terms of theory, methods and data is needed. Moreover, reliable, reproducible, and easy to access methods are especially important given the urgency in addressing ongoing environmental crises (e.g. rapid ecosystem degradation, global climate change, accelerated extinctions and biodiversity loss) (Stocker et al., 2013; Brondizio et al., 2019). Ecology is thus adapting rapidly to these critical challenges and is starting to adopt and develop novel theoretical and computational methods to answer a central problem: *How to synthesise and integrate ecological theory with big ecological data?* Answering this question requires an interdisciplinary approach that touches many fields, including: theoretical ecology, mathematical modelling, statistics, computer science and information sciences. For example, (Loreau, 2010) proposed a conceptual framework for integrating ecological theory by centering evolution as the link to unify ecology; and (Pavoine and Bonsall, 2011) proposed a semantic and mathematical formalization for unifying traits, species and phylogenetic diversity. The two approaches exemplify how evolutionary (ancestry) relationships between biological objects constitute a solid base to unify distant branches of ecology. From a statistical perspective, meta-analysis has been effective in synthesizing research evidence across independent studies, including unveiling general relations through a statistically sound framework(Koricheva et al., 2013).

Geospatial data constitute a crucial component for data fusion and harmonization; see (Wiemann and Bernard, 2016) for a review of methods for heterogeneous spatial big data fusion, and

removing bias using spatial data stratification methods (Wang et al., 2016). A clear example of geospatial data fusion is the building of Essential Biodiversity Variables (EBVs) to identify biodiversity and ecosystem change (Pereira et al., 2010). EBVs constitute a minimal set of critical variables aimed to standardize and harmonize global biodiversity variables. Originally proposed by the Group on Earth Observations Biodiversity Observation Network (GEO BON) to assess biodiversity change globally (Navarro et al., 2017); EBVs are now being used to predict global species distributions and potential scenarios for policy options (Pereira et al., 2013). EBVs integrate data in a standardised framework that describes spatial, temporal and biological organization (Schmeller et al., 2017). Recently, methodologies for building EBVs are drawing the attention of interdisciplinary research for reliability and data quality (Kissling et al., 2018). System designs and infrastructures for integrating heterogeneous big ecological data are emerging. Examples of these are the *citizen-based* bird observation network (eBird (Sullivan et al., 2009)), the TRY database for plant traits (Kattge et al., 2011), the PREDICTS project (Projecting Responses of Ecological Diversity In Changing Terrestrial Systems) (Hudson et al., 2014) and the Botanical Information and Ecology Network (Enquist et al., 2016). Despite the data heterogeneity and biased information against real absences (a consequence of opportunistic sampling), these types of infrastructures are able to collect sufficient quantities of data to perform statistical inference ((Hartig et al., 2012) and (Kelling et al., 2015)). The use of high performance computational technologies with novel statistical methods for representing and modelling big ecological data can provide deeper understanding of biodiversity evolution and its dynamics in a changing world (La Salle et al., 2016; Navarro et al., 2017; Schmeller et al., 2017). Moreover, its implications can be extended to other branches of ecology and Earth sciences. For example, a process-based approach by (Scheiter et al., 2013) showed how community assemblages can be integrated into dynamic vegetation models to increase the precision of climatic and Earth System models.

From a technical perspective, environmental and ecological data often come in matrix form

such that they can be stored and analysed efficiently with a relational database management systems (RDBMS) or other tabular data structure. RDBMS are reliable and sophisticated tools. An important feature is the possibility to extend their functionality with programming languages such as: C, Java, Python, R-Cran, etc.. This allows the combined use of an efficient data management system with a broad range of statistical libraries and programming methodologies. An example of this is the integration of spatial analysis tools into the RDBMS through the Postgis project (Ramsey et al., 2018); a set of compiled functions written in the Postgresql Procedural Language (PostgresPL) that interfaces with high level geospatial libraries (e.g. (GDAL/OGR Contributors, 2018), (Geometry Engine Open Source (Contributors), 2019) and (contributors, 2019)). Postgis adds GIS capabilities to the database engine, giving superior performance for querying information with geometric and topological features in space.

Integrating large datasets using only relational methods is computationally intensive. For example, matching data by a common feature involves the definition of join clauses plus computing the joined lookup between the pair of tables. The resulting product is often stored in volatile memory, a limiting factor when integrating large datasets. In a typical database design, table indices cost $O(log(n))$ in time, where $O(\cdot)$ is the classic *Big O*, a measure of computational complexity and n the size of the input dataset. A query involving multiple joins (from multiple data tables) can involve reverse and recursive lookups, that can increase the load from $O(n)$ to $O(n^k)$, where k is the number of data tables to join. Although this issue may be addressed with database design techniques such as normalization (Harrington, 2009) or caching (Altinel et al., 2002), the solution likely obfuscates the comprehension of the relational schema by adding unintuitive tables and other auxiliary information. It also requires a learning curve and expertise for implementation as well as increasing complexity when more datasets are added.

Data structures based on direct acyclic graphs (DAGs) are advantageous in relation to the above approaches. Traversing a relationship in a graph database has constant cost ($O(1)$) (Celko, 2014)

if the relations are defined explicitly for every node. Whenever a new dataset is added, a new link can be created to relate it with an existing record. Graph databases, however, are not as efficient at processing geospatial queries or handling simultaneous queries (Vicknair et al., 2010). In this sense, hybrid data management systems, capable of handling both paradigms (relational tables and DAGs), were proposed to overcome the limitations of both systems. However, to the best of our knowledge, these proposals have not been yet implemented (Grund et al., 2013), their code is closed (van Iersel et al., 2010) or their scope is not suited for environmental and spatial datasets, as is the case of the Reactome Database (Fabregat et al., 2018).

In this paper we propose an implementation of an open source knowledge engine (i.e. a hybrid database system) that stores, accesses and processes geospatial and temporal information, to integrate, analyse and visualise heterogeneous environmental, EVBs and big ecological data. The engine, named *Biospytial* (composed by the words *biodiversity, Python* and *spatial* and pronounced *Biospatial*) incorporates semantic relations that integrate data in a web of semantic knowledge able to represent complex graph (network) data structures.

Biospytial can be considered a component of traditional Spatial Data Infrastructure (SDI) because we simplify access and analysis of big datasets while satisfying the need of producing information for scientists and policy makers, among others (Hendriks et al., 2012). This is possible due to the engine's capability to identify intrinsic and extrinsic relationships within environmental and socio-economic processes. Therefore, the developed engine aims to serve SDI-based decision making framework as, for example, the European project INSPIRE.

The engine serves as a multi-purpose platform for modelling complex and heterogeneous data relationships using the power of graph theory. The current implementation uses occurrences data from the GBIF and its updated systematic classification (GBIF Secretariat, 2017) to build the acyclic graph of the *Tree of Life*. To exemplify the geospatial capabilities, some EVBs like: mean monthly temperature, elevation and mean monthly precipitation are also included in the engine. The pa-

per is structured as follows: The specification and general description of the engine is given in section 2. Section 3 proposes a methodology and software implementation for accessing biodiversity records arranged in a taxonomic tree. The graph of the *Tree of Life* is explained with examples for traversing and extracting spatial and taxonomic sub-networks. Section 4 explores the capabilities of the engine with a practical demonstration. It shows the syntax and discusses ways to interpret and traverse the knowledge graph. Finally, section 5 includes general conclusions, and future research directions.

2. An *Open Source* graph-based engine for geospatial analysis

The engine is able to import, organise, analyse and visualise big ecological datasets using the power of graph theory. It performs geospatial and temporal computations to synthesise information in different forms. The data can be queried and aggregated according to customised specifications defined by structural patterns called *graph traversals* (Rodriguez, 2015). The software has been developed with object-relational and object-graph mappings that use the object-oriented paradigm to abstract interrelated data into class instances (Juneau, 2018; Celko, 2014). In this sense, every record is represented as an instance of a certain class with its attributes mapped one-to-one to entries in a particular table (if it is stored in a relational database) or in a key:value hash table (if it is stored in a graph-based database). This approach allows the building of complex and persistent data structures that can represent different aspects of the knowledge base. It also allows the assemblage of automatic methods for exploring, filtering, aggregating and storing information.

2.1. System architecture

The engine is composed of three interconnected modules : i) A *Relational Geoprocessing Unit* (RGU), ii) the *Biospytial Computing Engine* (BCE) and iii) a *Graph Storage and Processing Unit* (GSPU) (see figure 1). Each module is arranged in virtual containers isolated as standalone applications (Docker Inc., 2019) running a common Linux image (Debian 8) as the base operating system. The virtual container technology creates a common environment for each module disregarding the complications of working with heterogeneous computer infrastructures (Pahl and Lee, 2015). Its design allows the replication of several instances of the same module in a single computer or in a distributed network. Containerised applications are easier to replicate and migrate compared to large data volumes and databases, which often involve resource intensive tasks in terms of energy, computing, network bandwidth and management. The idea behind containerization is: *move the processes not the data* and especially in the geospatial context, to perform spatial analysis where the data is located.

2.1.1. The Relational Geoprocessing Unit (RGU)

The RGU module undertakes the storage and raster-vector processing. It relies on high-level abstractions that represent geospatial data stored in relational tables. The supported geometric features are (multi)points, (multi)lines, (multi)polygons and multiple band raster data. It features a fully operational Postgresql (9.4.9) server (port: 5241) with geospatial extension (Postgis 2.3.1)(Ramsey et al., 2018) and libraries for handling geospatial data (GDAL, OGR 1.10.1)(GDAL/OGR Contributors, 2018), transformation between different geographic projections (PROJ 4.8, contributors (2019)), and computation of geometric operations (GEOS 3.6)(Geometry Engine Open Source (Contributors), 2019) (figure 1 b). The RGU image can be downloaded from: https://hub.docker.com/r/molgor/postgis_biospytial/

2.1.2. The Graph Storage and Processing Unit (GSPU)

This module hosts a graph database that stores data on nodes and their relations in a network structure called the knowledge-base (figure 1 a). The graph database system is an instance of Neo4J (3.1.3), an open source ACID-compliant transactional database management system with native graph storage and processing (Celko, 2014). It includes a web-based interface located in `http://<urlofhost>:7474`. The interface allows the inspection and visualisation of queries (subgraphs) using the Cypher interpreter (a No-SQL type declarative language for interrogating graph databases). The module also includes a plugin for spatial and topological lookups[4] and the *Awesome Procedures on Cypher* (APOC) [5]. ; an extension library with more than 300 procedures for data integration, graph algorithms or format conversion procedures. The GSPU image can be downloaded from: https://hub.docker.com/r/molgor/neo4j_biospytial/.

[4] `https://neo4j-contrib.github.io/spatial/0.24-neo4j-3.1/index.html`
[5] `https://neo4j-contrib.github.io/neo4j-apoc-procedures/index31.html`

2.1.3. *The Biospytial Computing Engine (BCE)*

This module provides the interface and processing toolbox for accessing, exploring and analysing data structures through the *Object Mapping* design. The container hosts a virtual environment and an *Anaconda* package manager (ANACONDA, 2016) that includes all the dependencies required by the engine. The core code of the engine is contained in a new Python package called *Biospytial* [6] (figure 1 c). The engine structure includes a `drivers` module to communicate with the graph database, the modules for accessing each dataset in the relational database; the module for graph traversals, data ingestion, griding systems, vector sketching, Jupyter notebooks; and external plugins like `spystats`, a Python port of GeoR (Diggle et al., 2002). The image can be downloaded from:

https://hub.docker.com/r/molgor/biospytial/

2.1.4. *Other features*

Scalable. The implementation includes scripts for automating the engine's deployment in a single host or in cluster mode. This mode provides a granular configuration for the allocation of resources and services in a distributed manner. For example, The BCE module can be hosted in a computer with high performance architectures or multiprocessing (e.g. MPI) capabilities.

Message broker. The engine includes a messaging service (Redis (Labs, 2012)) that delivers information between the different components. It also serves as an in-memory data structure storage and message broker. The storage is useful for interchanging data between different platforms and languages. For example, it allows export of the results into intermediary files (e.g. CSV or DBF) for use in other software (e.g. Team and R Development Core Team (2016) and Hornik (2012)).

Open Source - Open Contributions. The software used in all the modules has been released with Open Source and Free Software licenses which allow users to reproduce, modify and publish their

[6] `https://github.com/molgor/biospytial`

research source code. The engine was developed using best practices for scientific computing (Wilson et al., 2014b),data transparency and reproducibility (Perkel, 2018).

2.1.5. *Access to the engine*

There are two ways of accessing the engine. One is through a command-line interpreter based on the `iPython` console (Perez et al., 2007). The other is with an online Jupyter notebook server (Kluyver et al., 2016) (`localhost:8888`). The Jupyter notebook is a web-based interactive Python interpreter that renders MarkDown documents, plots and images in the browser . Analysts can create files in a *notebook* format (.ipdb) and share the results on-line. Peers can visit the notebook's url, read the document, run the code, replicate the analysis, access the variables, import other libraries, modify the analysis and export it into different formats (e.g. PDF, Latex or HTML).

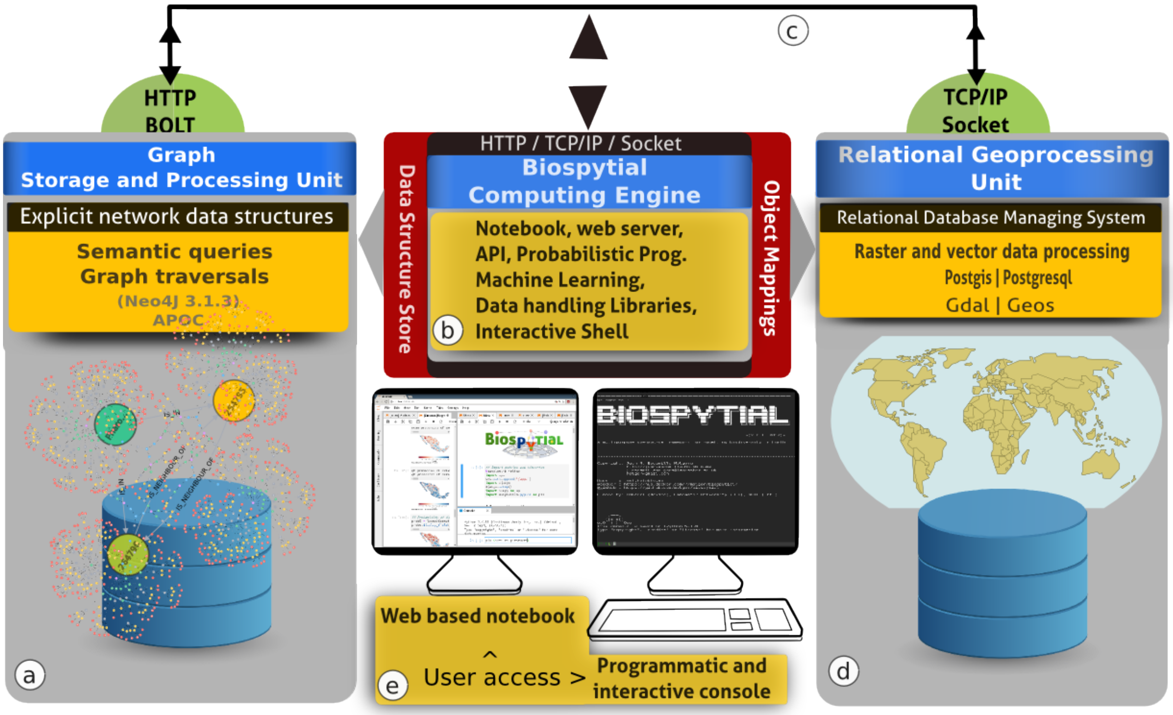

Figure 1: The Biospytial System with the three interconnected modules. a) The GSPU, where semantic queries and graph traversals take place. b) The BCE, where object mappings, web services and the modelling framework takes place. It includes several libraries for performing exploratory analysis as well as Bayesian statistical inference and prediction using the probabilistic programming language: PYMC3; c) All the components can be allocated in the cloud and are connected using virtual and physical networks. d) The RGU, where the geoprocessing and spatial indexing occurs, storing efficiently any raster and vector data sources. e) Interactive access is possible in two ways: using an online web notebook (Jupyter) or an interactive console (iPython).

Table 1: Principal software components of the Biospytial Knowledge Engine System

Software name	Version	Description
**Biospytial Computing Unit**	Debian GNU/Linux 8.6	Container OS image
Conda	4.3.30	Package manager optimized for Data Science
Python	2.7.11	Programming language (scheduled update for v.3.x)
R-base	3.2	Language and software environment for statistical computing
Jupyter	1.0.0	Interactive web application for reproducible computational workflows
Scipy	1.01	Python library for numerical and scientific computation
Pandas	0.19	Python library for data structures and data analysis
Geopandas	0.3	Extension of Pandas to support geospatial data
GDAL	2.1	Library for converting and processing geospatial data
Shapely	1.5.16	Python library for manipulation and analysis of geometric objects in the Cartesian plane
Django	1.8.4	ORM, web framework and standalone server
Py2neo	3.11	A client python library and toolkit for working with Neo4j
Pymc3	3.4,1	A Python based Probabilistic Programming Framework
Patsy	0.4.1	A Python library for describing statistical models
**Relational Geoprocessing Unit**	Debian GNU/Linux 8.6	Container OS image
Postgresql	9.4.9	Relational database management system
Postgis	2.3	Spatial extension for Postgresql
GDAL	1.10.1	Library for converting and processing geospatial data
GEOS	3.6	Geometric and Topological library
Proj4	4.8	Coordinate transformation software
**Graph Stor. and Process. Unit**	Alpine Linux 3.5	Container OS image
OpenJDK	IcedTea 3.3	Open Source Java compiler and virtual machine
Neo4J	3.1.3 (C.E)	Graph Database Management System
APOC	3.1.3	Utilities, graph algorithms and common procedures for Neo4j
**Message Broker**	Redis 5.0.3	a Key-value data structure store

2.2. Knowledge representation

The engine uses two database paradigms to store and represent data: a relational system with tables connected by primary and foreign keys and directed acyclic graphs (DAGs) where the data are stored as nodes (with associated attributes) and edges representing relations between nodes. Each node can belong to one or many classes. In our implementation, the relationships are semantic phrases that refer to location (e.g. *"IS IN"*), ancestry (*"IS PARENT OF"*) or topological features (*"IS CONTAINED IN"* or *"IS NEIGHBOUR OF"*). Thus, the engine uses explicit semantic relations between nodes to build a network of semantic information. The union of all these relationships is what we call *knowledge graph.*

The event of a species s being recorded at location l can be represented as a node of the class *Species* connected to a node l of class *Cell* using the relation *IS_IN*. The *Cell* nodes are contained in a regular lattice (grid) and are instantiated by a class that implements a geospatial type defined by a polygon that acts as a geometric border. As an example, figure 2 shows this diagram for the bird family of quetzales (Trogonidae) found in southeast Mexico. The node in red represents the species: *Pharomachrus mocinno.* The nodes in blue are two *Cell* types that associate the locations where *P.mocinno* was found. The arrows indicate the directional relationships between the nodes. The graph database allows easy manipulation of these nodes, their relations and combinations. At the same time, the selected pattern can be filtered by chosen attribute values to generate customized design matrices.

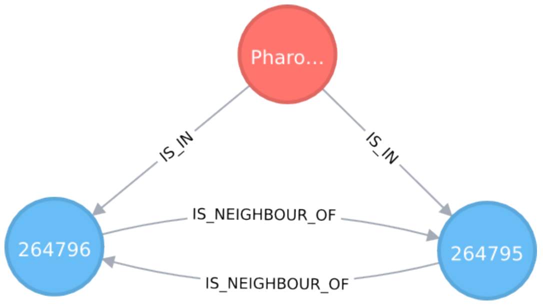

Figure 2: The graph showing the connection between a *Species* node and two *Cell* nodes. Here: the species is *Pharomachrus mocinno* (Quetzal) and the number shown in each Cell node is its respective ID number. This is an actual visualisation taken from data stored in our Knowledge Graph.

2.3. Integrating data with graph structures and object mappings

The *Object Mapping* approach serves to communicate different database management systems (relational or graph-based). A high level Python-based Object Relational Mapping (ORM) library (`Django` (dja, 2018)) was used to communicate with the RDBMS and the other components of the engine. It includes a high level interface to translate sentences from the SQL declarative language into method calls from the object-oriented paradigm. Vector and raster operations are possible via the Open Source Geographic Information System (OSGIS) for Postgresql (Postgis (Ramsey et al., 2018)). Currently, all the spatial and tabular data are stored in the RDBMS.

The *object mapping* on the graph database system is achieved with `py2neo`, a client library and toolkit for communicating with the Neo4j database management system [7] within the Python programming language(Small, 2017). Topological information like neighbouring cells and nodes contained within cells are stored as semantic relations. Some preprocessed information is stored in the knowledge graph. This includes some parameter estimates, aggregated data, summary statistics and associated raster metadata.

The procedure for adding data into the engine varies according to the data format (tables or linked data) and requires a new class to be created. The class is responsible for accessing and managing data in both database systems. It includes specifications for storage, conversion between formats and analysis. A simple implementation would include: the name and type of the attributes; the name of the table (for the case of RDBMS), the node type and incoming and outgoing relations between nodes (for graph-based datasets). Detailed information on all these procedures is given in the supplementary materials.

2.4. Graph Traversals

As explained above, the *Knowledge Graph* is the totality of nodes and relationships stored in the database. Each node represents a type (defined by a class) of data or a more abstract concept

[7] `https://neo4j.com`

that generalises certain sets of data. Each node has associated edges to other nodes, as well as a list of attributes. In the example given in figure 2, the node is of type *Species* and one of its attributes is *name* with the associated value *P.mocinno*.

The graph engine can search and extract information from the knowledge graph using recursive rules based on semantic predicates. Typically, the search selects one, or several, nodes and continues visiting (traversing) other connected nodes that match the specified criteria until the relationship is exhausted or a depth threshold has been reached. The resulting selection of relationships and nodes is a subgraph of the knowledge graph. We call this structure a *pattern* and the set of rules that select a pattern is a *graph traversal*.

Graph traversals can be translated into data matrices that can be analysed within the scope of model-based geostatistics (Diggle et al., 2002) or areal unit modelling in lattice systems using Gaussian Markov Random Fields (Besag, 1974; Besag et al., 1991; Rue and Held, 2005). Also, they can be analysed with network theory to answer questions about resilience, connectedness, modularity or invariants across scales. The objects are compatible with the open source libraries for statistical inference and network analysis. Libraries already included in the engine are: NetworkX (Hagberg et al., 2008), StatsModels (Seabold and Perktold, 2010) and PyMC3 (Salvatier et al., 2016).

2.4.1. Complex queries

Our implementation enforces the use of *lazy evaluations*, in which the evaluation of an expression is delayed until the value is needed and not directly upon the instantiation (Hudak and Paul, 1989). This helps in the creation of data primitives that can be composed into higher level graph traversals without the need to load in all the data. The design allows the request on demand of partial evaluations for a given traversal. This abstraction helps to explore, design and automate the discovery of relevant patterns and structures. A concrete example of this design is showed in section 3 with the analysis of local taxonomic trees, when the tree object is instantiated, it exists only as an abstract data container with no data requested to the database. As such, if an analyst

is interested in studying the different species of bats (*Order:Chiroptera*) within this tree, she will need only to consider the descendant (children) nodes of the node *Chiroptera* of type *Order* (See section 5.1 for a practical example).

Some traversals are exclusive of certain node classes and, therefore, have associated special methods. This is the case for nodes of type *Cell* which include a method for extracting neighbouring cells. Figure 3 shows an example of this where a selection of cells was obtained first by requesting all the occurrences of the Family *Culicidae* and then traversing through the associated cells and their corresponding neighbours using the method getNeighbouringCells() twice.

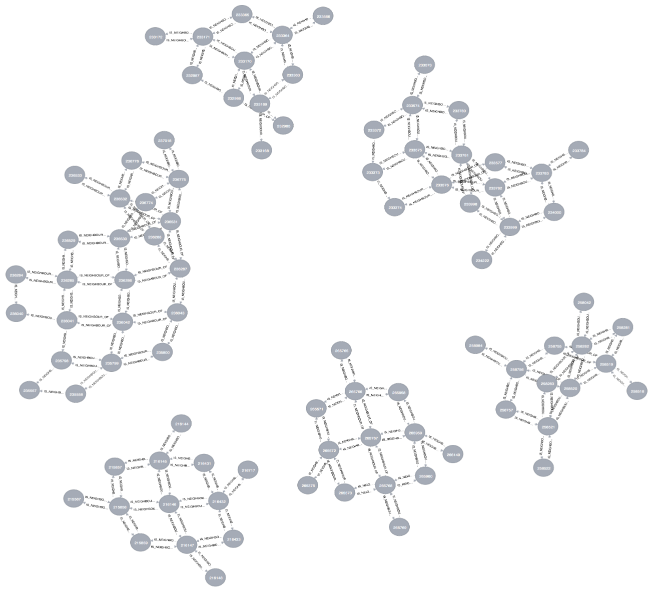

Figure 3: A subgraph from the Knowledge Engine that shows the second order degree of neighbouring cells where at least one occurrence of any type of mosquito (family *Culicidae*) was registered. This query exemplifies the use of recursive lookups. In this case the relationship "IS_NEIGHBOUR_OF" is traversed twice.

2.5. Geospatial management and processing

The engine supports and processes geospatial information using the GDAL/OGR library (GDAL/OGR Contributors, 2018).The default Coordinate Reference System (CRS) is the WGS84 with geographic coordinates. However, it is possible to use and reproject the data into any other CSR. This feature is supported by the *Proj4* library (contributors, 2019). See section 5.8.1 for a concrete example of this.

2.5.1. Vector data

Vector data are represented with tabular data structures. These tables should include the following information: at least one column with a unique identifier (id) for each record, one column for each type of feature, and at least one geographic column to represent the geometric shape of each record. The available geometric types are: points, multiple points, polylines, multiple polylines, polygons and multiple polygons. Each type of dataset corresponds to both a vector layer and a table in the RDBMS. A mapping between the table structure and the engine needs to be created in the same way as described in section 2.3. For large datasets the engine uses indexing methods for optimal performance on accessing and querying the data. Additional information is provided in the supplementary material. 14.4

2.5.2. Raster data

Raster data are represented as a table stored in the RDBMS together with its corresponding metadata. The table has three columns: a primary key (id); a Binary Large Object (BLOB) data type (encoding a stack of matrices) that represent a multiband image; and a reference to a file where the metadata is stored. The metadata includes: projection type, affine parameters, datatype for entries (binary, integer, float) and other information related to provenance.

Ingesting raster data into the engine involves two steps, i) the dataset is partitioned in to regular tiles; and ii) each tile is converted into a BLOB string and inserted into the table. Data ingestion scripts can be found in the supplementary materials.14.7

The *Object Mapping* design is used to specify the definition of a *RasterData* type and its associated operations. The implemented class includes methods for clipping, downscaling, aggregating, exporting to image formats (Geotif and PNG), visualising, intersecting vector data, extracting metadata and conversion to arrays. An extended class for Digital Elevation Models (DEM) is also implemented to generate *on the fly* aspect, slope and shaded relief (figure 4), without requiring the datasets (derived DEM products) to be stored directly in memory.

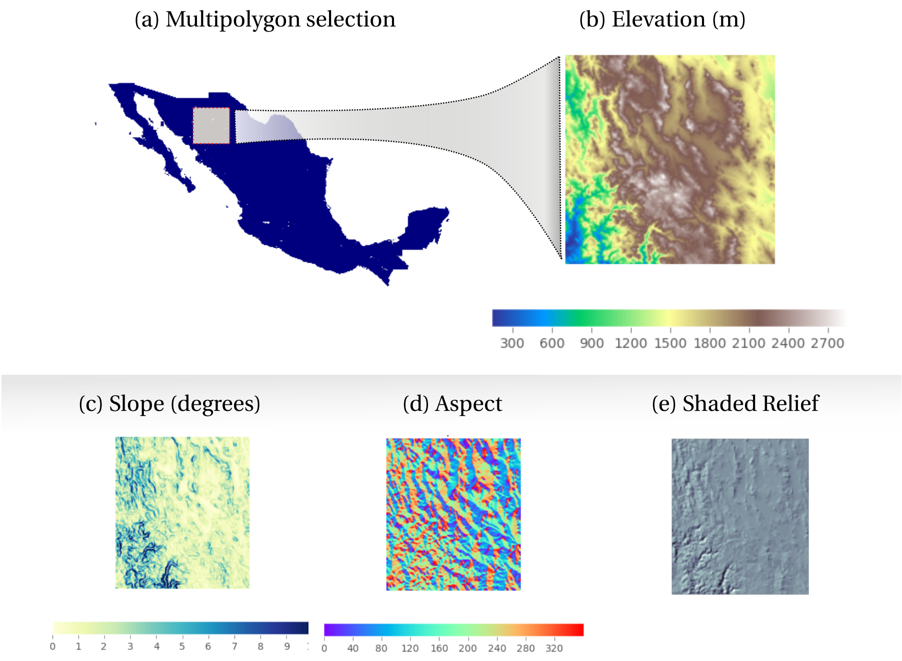

Figure 4: Raster manipulation in the knowledge engine. a) a multipolygon selection corresponding to Mexico, an instance from the class `Country` that maps into the *WorldBorders* dataset. b) An `Elevation` object (class `RasterData`) instantiated with a customized polygon, in this case a subregion of the object Mexico. c), d) and e) are `RasterData` objects derived from the Elevation object. The data and visualisations were produced using the engine's raster API. The code for generating these figures are in supplementary materials.

On instantiation, a *RasterData* object requires the definition of a boundary object passed as argument. This object should be a polygon type `django.gis.contrib.GEOS.Polygon` or a text string defining a polygon in the *Well Known Text* (WKT) format. The resulting selection can be transformed to a dataframe or n-array for statistical modelling. As in the other data structures, whenever a new raster model is added a new model class should be included (See Supplementary Materials) 14.7.

3. Using Biospytial to analyse the Tree of Life

In this section we propose a process for integrating spatio-temporal data together with graph traversals to represent tree structures using taxonomic and topological relationships within the knowledge engine. The graph traversals use biodiversity occurrences and environmental data to build complex structures to analyse, visualise and caracterize biological occurrences in different forms. The structure restricted to the taxonomic classification is an acyclic graph (tree) in which all the species occurrences constitute leaf nodes. We call this structure the *Tree of Life* (ToL) and propose a set of graph traversals to retrieve subsets of the ToL constrained to arbitrary taxonomic groups, spatial regions or temporal ranges. Several class definitions for handling taxonomic trees are implemented, making it possible to automate tasks for unveiling patterns. For a detailed definition of terms and computational structures see suplementary materials II.

3.1. Study Area

The study site selected was restricted to Mexico since (i) Mexico is in the list of Megadiverse countries (UNEP/CBD, 2002, 2016); (ii) the territory contains a diverse range of the world's climatic regions (Vidal Zepeda, 2005; Rzedowski, 2006); (iii) the country has policies for publishing open environmental data, including centralized repositories of curated data related to biodiversity, conservation, ecosystem services, land cover and satellite sensor imagery (Sarukhán et al., 2009). The data in the study area provide a concrete example of the engine's capabilities.

3.2. Data used

The species occurrences were obtained from a snapshot taken from the global GBIF database on September 2016 (GBIF Secretariat, 2015). The data was filtered to only include the occurrences located within the Mexican borders. The total number of occurrences is 3,242,746 distributed in 54,828 species, 10,781 genera, 2,300 families, 543 orders, 113 classes and 42 phyla, with acquisition years ranging from 1819 to 2016. The taxonomic classification was taken from the GBIF

Taxonomy Backbone (GBIF Secretariat, 2017). Each occurrence record has information of species name, location (point coordinates in WGS84) and acquisition date, and represents the observed presence of a certain species, therefore it is only based on presence-only records.

The digital elevation model (DEM) *ETOPO1 1 Arc-Minute Global Relief Model* (Amante and Eakins, 2009) was used at a spatial resolution of 1 minute. Precipitation, temperature (maximum, mean and minimum), solar radiation, wind speed and vapor pressure were obtained from the World Climatic Data *WorldClim* version 2 dataset (Fick and Hijmans, 2017). Each variable is a 12 band raster model with 1 km^2 spatial resolution that aggregates monthly average values from the years 1970 to 2000 per month, each band corresponding to each month. The data license for *WorldClim* restricts the redistribution of the data. Therefore, users need to download it and import it into the engine via an automated script:

```
raster_api.bash_raster_tools.migrateToPostgis.bash
```

The engine includes functions for generating grid systems at different spatial resolutions. When the grid system is created it stores a vector representation in the RGU and a network representation in the GSPU. The functions for generating the grid systems are located in the library: `mesh.tools.py`.

3.3. *Traversals on the Knowledge Graph*

The taxonomic tree structure was built with the relation: `IS_PARENT_OF`[8] following the taxonomic classification of the occurrence data and the GBIF *Backbone Taxonomy*(GBIF Secretariat, 2017). Each occurrence had a location attribute matched with environmental data (e.g. elevation or WorldClim) using a *point in polygon* query to the RGU. The spatial structure was built using the relations `IS_IN` and `IS_CONTAINED_IN` in accordance with topological relationships based on the DE-9IM model (Egenhofer and Franzosa, 1991; Clementini et al., 1993) (standardised by (Herrig, 2011)).

[8] Conversely, `Has_Children`

The main travesal structure is defined in the *TreeNeo* class. Each instance comprised of an area defined by a spatial polygon and a list of occurrences contained on it. The graph traversal was built recursively using the systematic classification of organisms, starting from the GBIF occurrences as leaf nodes and progressing through the parent nodes until the traversal reaches the node with no parent. That is, it begins by the species level and finalises in the root node. On each step, the algorithm fetches the available nodes and group them by their corresponding parent node, generating a set of parent nodes and their associated children. Each of these duples (parent,children) are incorporated into a `LocalTree` object that parses the relevant information into several attributes. This process is applied recursively on each derived parent node of the previous step. The recursion is terminated when the set of parent nodes is empty, generating the desired tree data structure. When this happen the `LocalTree` object is wrapped into a *TreeNeo* instance that extends some additional methods like: manipulating and querying trees, nodes and multiple taxonomic groups as well as graph analysis and exportation to common exchange formats (e.g. graphml, data frames, png, geotif or shapefiles). In addition, all the spatial structures were implemented with Open Source Geospatial(OSGEO) standards (Kemp and Haklay, 2014) to facilitate the migration to other language and platforms. A visualisation of this traversal is showed in figure 5.

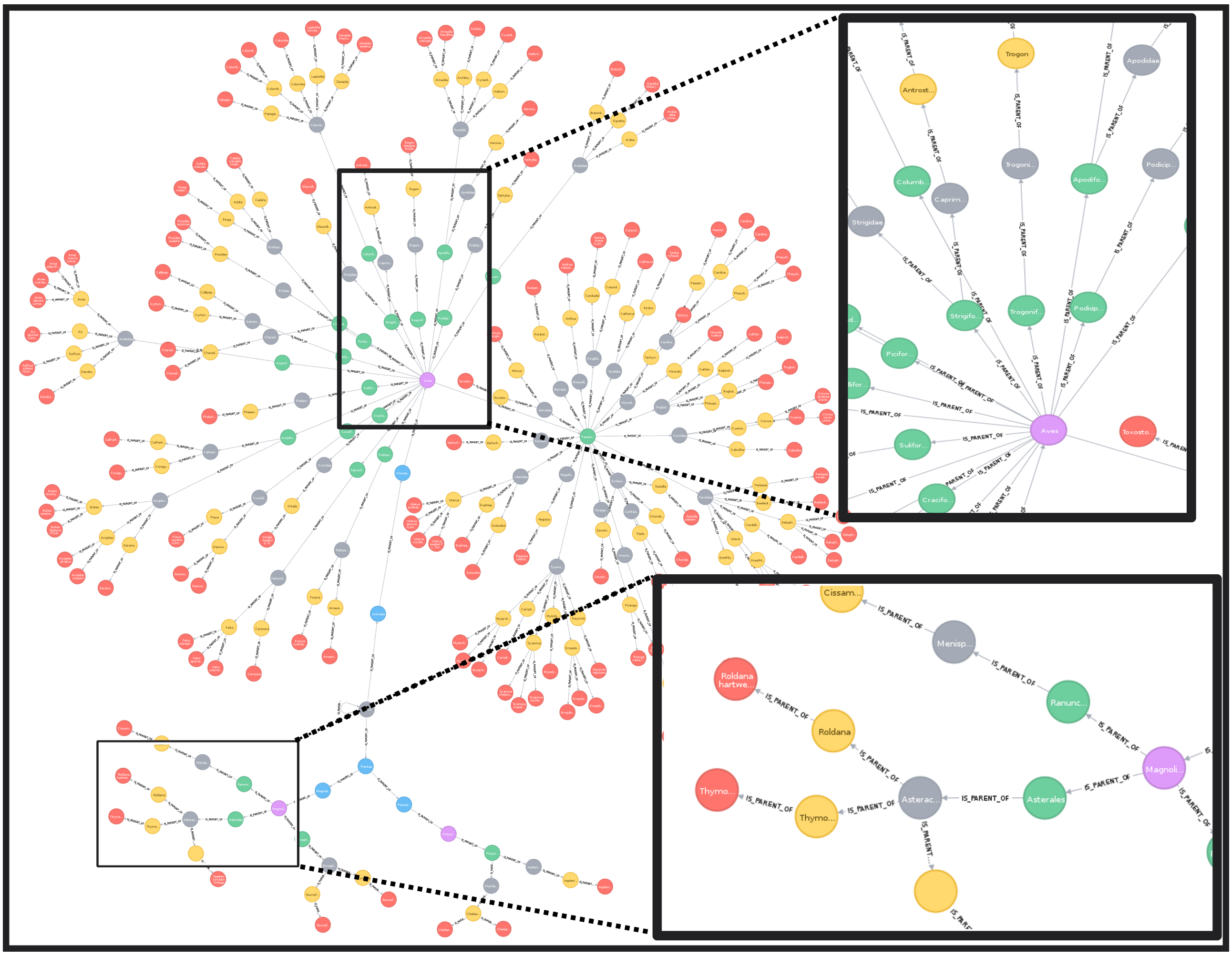

Figure 5: A visualisation of a Local Taxonomic Tree built with the relationship: `IS_PARENT_OF`. The rectangles show zoomed areas in different sections of the tree (upper region for Birds (Order Aves), lower for plants (Order Magnoliopsida)). Colored nodes indicate distinct taxonomic levels (red : species, yellow: genera, grey: families, green orders, purple: classes).

4. Worked examples

This section is a case study for analysing the frequency of coexistent taxonomic groups in all the available dataset restricted to arbitrarily chosen branches of the Tree of Life (ToL) and included in a list of threatened species. These types of analyses are important in conservation studies, where the characterisation of umbrella (or other surrogate) species constitute the basis for protecting a significant number of associated species (Andelman and Fagan, 2000; Drever et al., 2019). To account for this effect, we chose the jaguar (*Panthera onca*) as the species of interest. This due to its preference for undisturbed ecosystems (Thornton et al., 2016) and its wide geographic required range; $181 \pm 4 km^2$ for females and $431 \pm 152 km^2$ males (de la Torre et al., 2017).

4.1. Additional data used

We use the IUCN Red List of Threatened Species (Red List) (IUCN, 2019) in Mexico to account for the proportion of species (critically endangered, endangered or vulnerable) associated with the presence of jaguars. For aggregating the data into taxonomic trees (i.e `TreeNeo` objects), as well as for extracting their corresponding environmental covariates, we used a 0.05° (c. $5 km$) resolution grid intersected with the terrestrial regions of Mexico and Central America. The used grid is included in the default installation of the engine and therefore, all the analysis performed in this example is reproducible.

4.2. Methodology

We first obtain the grid cells with at least one occurrence of jaguar. As these cells are `Cell` objects, it is possible to extract associated neighbouring cells using the method: `getNeighbours`. We can apply the same method recursively four times to obtain a list of neighbouring cells within a 4 degree neighbourhood. For each cell, we obtain the local taxonomic tree. The resulting trees are merged into a single tree that contains the union of all the nodes of all the local trees. Therefore, the aggregated tree contains all the known co-occurrences of jaguar in a neighbourhood of degree 4.

The resulting tree is filtered to select only the nodes that match the Red List of threatened species. A new tree object is created using the selected nodes, an operation know as *trimming*.

To provide an estimate of which nodes co-occur more often with jaguars, we rank all the nodes in the merged tree using the frequency of presence of each node at each neighboring cell. To show the raster querying capabilities, we contrast these results with environmental ranges of: jaguars, threated species and the entire country using the `raster_api` module. Finally, we provide methods for interactive visualisations of the extracted spatial data and the network structure.

4.3. *Results of the worked example*

The taxonomic analysis of the most abundant families across all neighbouring cells where: Muridae (rodents, 29%), Phyllostomidae (a family of bats, 23%) and Cervidae (deers, 15%) for the case of mammals. For of parrots (Order Psittaciformes) the most frequent species was *Ara militaris* (military macaws, 2%) and several species of the genus Amazona, accounting for 16% in total. Although the order Psittaciformes was abundant (23%) in the group of vertebrates, the most abundant taxon (*A. militaris*) only co-occurred 2% of the time with the jaguar's neighbouring cells. This result shows the great diversity of species within the group of parrots. This is consistent with natural history records, where it has been described that these species inhabit humid forests, wooded foothills and canyons in elevation ranges between 500 and 1,500 metres above sea level EOL.

The same analysis applied to plants showed that the most abundant genera were: the epiphyte Tillandsia (19%), the *Coussapoa oligocephala* (6%) , Pouteria (several species, 9%), *Cedrela odorata* (3.2%), which are tropical trees, and other trees not typical from tropical rain forests like Oreopanax (9%) and Quercus (6%). Longer lists of the most abundant taxa detailed in the worked example as well as their interactive version in the Jupyter notebook are provided in the file `examples/Official Demo Co-occurrences.ipynb` located in the Biospytial repository. A visualization of the threatened taxa tree is shown in figure 9 for: kingdoms, phyla, classes and orders.

From an environmental perspective there is a clear concordance between jaguars' habitat and

threatened taxa, when compared to all Mexico, for mean temperature (fig 6a), annual rainfall (fig 6b and wind speed (fig 6d). In fact, threatened species and jaguars show environmental modalities distinct from all Mexico. To create the plots we used the library seaborn. Detailing the process for creating these graphs is out of the scope of the present tutorial. However, the snippet has been included in the interactive notebook.

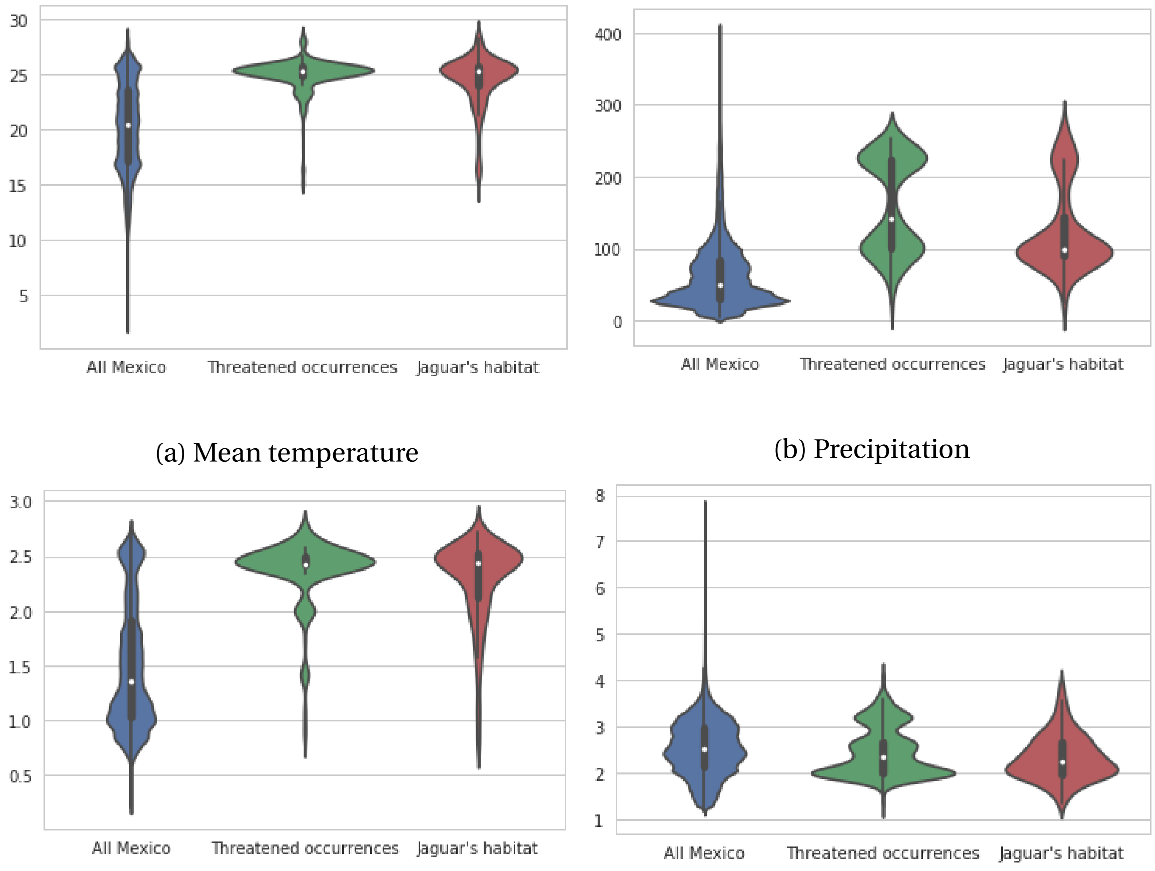

(a) Mean temperature

(b) Precipitation

(c) Water vapor pressure

(d) wind speed

Figure 6: Comparison of mean annual environmental ranges between treatments: All Mexico, threatened taxa and cells with occurrences of jaguars. See next section for more details.

5. Tutorial

The time for executing the following example varies considerably depending on the group of interest, the size of the neighbourhood and the computer platform. A quick workaround to speed up the processes is to reduce the number of neighbouring cells (order of the neighbourhood), for example a degree of 1.

A reproducible version of this tutorial is included in the Biospytial source code (inside the folder `examples/`) in an interactive jupyter notebook file named:

```
Official Demo Co-occurrences_jaguar.ipynb
```

The following section is a static version and is subject to minor modification to fit the layout and format of this PDF version.

5.1. Selecting the node Jaguar

We begin by selecting the node in the ToL corresponding to the genus *Panthera*. This node is linked to some `Species` and `Family` type nodes and also has links to `Occurrence` nodes, where the information of location and time is stored. To start the traversal we need to first select this node. To do so we use the function `pickNode` using the following syntax:

```
pickNode(<Type of Node>,'name of the node')
```

In the example below we see how to load the `pickNode` function and the appropriate node class (in this case `Genus`).

```
from drivers.graph_models import Genus, pickNode
jaguars = pickNode(Genus,"Panthera")
```

The variable *jaguars* is now an instance of the class **Genus**. As such, it has associated attributes and methods. Its string representation is the following:

```
jaguars :  <TreeNode type: Genus id = 2435194 name: Panthera>
```

We proceed to traverse through all the cells where any occurrence of the *Panthera* genus was registered. To do so we call the attribute *cells*. This attribute is abstracted with *lazy evaluation*. Therefore, to fetch all the associated data we need to convert the object into a list (or a partial list using an iterator).

```
cells = list(jaguars.cells)
print("cells has %s elements"%len(cells))
```

```
cells has 62 elements
```

The resulting list has cell instances, each one connected to other cells by the relation: 'IS NEIGHBOUR OF'. Accessing their related cells is achieved by the method:

```
cell.getNeighbours(with_center=[Boolean],order=[Int])
```

where the parameter `with_center` returns the center of the neighborhood, and the parameter `order` the size (in number of cells) of the neighborhood (this value can be reduced to 1 for faster computation). In our case, we apply this method for each cell using a `map` function with a `lambda` expression.

```
neighbours = map(lambda cell :
                 cell.getNeighbours(with_center=True,order=4),
                 cells)
```

Lambda expressions are part of the Python syntax and are used to create anonymous functions. The *map-lambda* technique allows the definition of statements that are applied to all the elements of a list, returning a new list of objects obtained by evaluating the lambda expression on every element of the given list. Along this tutorial, the use of the *map-lambda* technique is frequently used. Whenever this expression comes it is recommended to read the form:

```
map(lambda x : <something involving x> , some_list)
```

As, "for all *x* in `some_list`, do *something involving*`x`". In the example above, the object `neighbours` is a list of neighbouring cells obtained from the method `getNeighbours` available on each cell instance (i.e. each element of the `cells` list).

As this list is composed of list-type elements (i.e. it is a nested list), we need to reduce it into a single list composed of only cell instances, a process known as flattening. To do this simply reduce the list as this.

```
# the + operator between two list instances merges them together.
neighbours = reduce(lambda list_a , list_b : list_a + list_b, neighbours)
```

The *reduce* function is a Python standard function that receives a two parameter function (in this case a lambda expression receiving parameters `list_a` and `list_b`) and the nested list `neighbours`. The *reduce* function applies the lambda expression to the first pair of elements of the list and iteratively applies the result to the next element. As the sum operation between lists (+) merges the elements of both lists into a single list, performing this operation across the entire nested list `neighbours` result in a flattened list.

The resulting `neighbours` list now has 2497 Cell nodes. In the current implementation the name of the Grid (where all the Cells are contained) is called *mex4km*. We can display the first three elements as:

```
neighbours[:3]
```

```
[< Cell-mex4km id = 234686 >,
 < Cell-mex4km id = 234685 >,
 < Cell-mex4km id = 234684 >]
```

5.2. Converting cells to local taxonomic trees

We obtain the ToL inside each Cell node by extracting the occurrences inside each cell (using the method `occurrencesHere`) and plugging them into the *TreeNeo* constructor. The name *TreeNeo* is used because the storage backend is the Neo4j graph database.

```
from drivers.tree_builder import TreeNeo
cell_1 = neighbours[1]
```

```
tree_1 = TreeNeo(cell_1.occurrencesHere())
print(tree_1)
```

```
<LocalTree Of Life | Root: LUCA - n.count : 1062- >
```

The `n.count` value indicates the number of total occurrences. We can generate all the trees iteratively using a mapping from the `TreeNeo(cell.occurrencesHere())` through all neighbouring cells. This may take some time depending on the number of cells and occurrences on each cell. For reducing this time go to subsection 5.1.

```
sample_trees = map(lambda cell : TreeNeo(cell.occurrencesHere()),neighbours)
```

As in the last example, we can see basic information as object description. Here the first four elements are shown.

```
sample_trees[:4]
```

```
[<LocalTree Of Life | Root: LUCA - n.count : 3- >,
 <LocalTree Of Life | Root: LUCA - n.count : 1062- >,
 <LocalTree Of Life | Root: LUCA - n.count : 151- >,
 <LocalTree Of Life | No record available: - n.count : 0- >]
```

The value `n.count` indicates the number of occurrences found for the present node. It is possible to have empty trees, when no occurrences were found. This is shown with the text `No record available`.

5.3. *Exploratory analysis on a single Tree*

We select a tree in this example and explore informative data.

```
tree = sample_trees[1]
```

The object `tree` wraps the entire tree structure. All `tree` objects have as their starting node the root of the Taxonomic Tree, representing all known life.

```
root = tree.node
```

`root` node is similar to `Family` node, `Genus` node, etc. They all belong to the class: `TreeNode`. We can access a specific child node with the prefix `to_[name of taxon]`. For example, accessing the node 'Animalia' can be done with:

```
animalia = root.to_Animalia
animalia
```

```
<LocalTree | Kingdom: Animalia - n.count : 742- | AF: 0.05>
```

5.3.1. Traverse by children nodes

We can concatenate this method until the children attribute is empty. If running Biospytial in an interactive session (like a Jupyter notebook or iPython) we can use the key [TAB] to autocomplete and show the available nodes. For example, the family of rodents *Muridae*.

```
root.to_Animalia.to_Chordata.to_Mammalia.to_Rodentia.to_Muridae
```

```
<LocalTree | Family: Muridae - n.count : 34- | AF: 0.05>
```

5.3.2. Tree traversal by taxonomic level

The taxonomic levels (e.g., families, orders, etc.) are stored as atributes of the `TreeNeo` class. For example, to see the available `phyla` in this tree do:

```
print(tree.phyla)
```

```
[<LocalTree | Phylum: Chordata - n.count : 740- | AF: 0.05 >,
 <LocalTree | Phylum: Arthropoda - n.count : 2- | AF: 0.05 >,
 <LocalTree | Phylum: Bryophyta - n.count : 99- | AF: 0.05 >,
 <LocalTree | Phylum: Magnoliophyta - n.count : 175- | AF: 0.05 >,
 <LocalTree | Phylum: Mycetozoa - n.count : 46- | AF: 0.05 >]
```

and for some families inside this tree:

```
print(tree.families[:5])
```

```
[<LocalTree | Family: Menispermaceae - n.count : 3- | AF: 0.05 >,
 <LocalTree | Family: Piperaceae - n.count : 7- | AF: 0.05 >,
 <LocalTree | Family: Lauraceae - n.count : 2- | AF: 0.05 >,
 <LocalTree | Family: Acanthaceae - n.count : 7- | AF: 0.05 >,
 <LocalTree | Family: Plantaginaceae - n.count : 1- | AF: 0.05 >]
```

5.4. *Tree operations*

Tree objects allow symbolic operations for adding (merging) and intersecting other tree objects. These operations are currently implemented as `sum (+)` and `intersection (&)`. These operations can be applied to arbitrary number of trees and it is useful in comparative studies that require the calculus of (α, β, γ)-diversity using a combination of these operations (Whittaker, 1972). Mathematically, these operations are equivalent theoretic *set* operations acting at the occurrence level. As an example consider the following: let `t1` and `t2` be two trees from the list of `sampled_trees`, i.e.

```
t1 = sample_trees[1]
t2 = sample_trees[2]
```

5.4.1. *Addition*

Adding trees is equivalent to merging them. That is, making the union of all the nodes (inter nodes and leaves). The tree objects (`TreeNode` and `TreeNeo` classes) allow the use of the + operation. For example, the merge tree of `t1` and `t2` is obtained with:

```
t3 = t1 + t2
```

We can see the effect of this by selecting the nodes of a certain taxonomic level, for example, the classes of `t1` and `t2` are:

```
print(t1.classes)
```

```
[<LocalTree | Class: Myxomycetes - n.count : 46- | AF: 0.05 >,
 <LocalTree | Class: Bryopsida - n.count : 99- | AF: 0.05 >,
 <LocalTree | Class: Amphibia - n.count : 1- | AF: 0.05 >,
 <LocalTree | Class: Aves - n.count : 667- | AF: 0.05 >,
 <LocalTree | Class: Reptilia - n.count : 2- | AF: 0.05 >,
 <LocalTree | Class: Mammalia - n.count : 70- | AF: 0.05 >,
 <LocalTree | Class: Liliopsida - n.count : 36- | AF: 0.05 >,
 <LocalTree | Class: Magnoliopsida - n.count : 139- | AF: 0.05 >,
 <LocalTree | Class: Insecta - n.count : 2- | AF: 0.05 >]
```

```
print(t2.classes)
```

```
[<LocalTree | Class: Protosteliomycetes - n.count : 2- | AF: 0.05 >,
 <LocalTree | Class: Myxomycetes - n.count : 112- | AF: 0.05 >,
 <LocalTree | Class: Agaricomycetes - n.count : 4- | AF: 0.05 >,
 <LocalTree | Class: Liliopsida - n.count : 8- | AF: 0.05 >,
 <LocalTree | Class: Magnoliopsida - n.count : 25- | AF: 0.05 >]
```

```
print(t3.classes)
```

```
[<LocalTree | Class: Protosteliomycetes - n.count : 2- | AF: 0.05 >,
 <LocalTree | Class: Myxomycetes - n.count : 158- | AF: 0.05 >,
 <LocalTree | Class: Agaricomycetes - n.count : 4- | AF: 0.05 >,
 <LocalTree | Class: Bryopsida - n.count : 99- | AF: 0.05 >,
 <LocalTree | Class: Amphibia - n.count : 1- | AF: 0.05 >,
 <LocalTree | Class: Aves - n.count : 667- | AF: 0.05 >,
 <LocalTree | Class: Reptilia - n.count : 2- | AF: 0.05 >,
```

```
<LocalTree | Class: Mammalia - n.count : 70- | AF: 0.05 >,
<LocalTree | Class: Liliopsida - n.count : 44- | AF: 0.05 >,
<LocalTree | Class: Magnoliopsida - n.count : 164- | AF: 0.05 >,
<LocalTree | Class: Insecta - n.count : 2- | AF: 0.05 >]
```

5.4.2. Intersection

Intersection is applied through the & operation and it is equivalent to the intersection of sets with the *difference* that it is only applied to the leaf nodes, that is, the **Occurrence** nodes. Once the leaf nodes are selected, the algorithm propagates through the parent nodes until it reaches the root node. To see the formalization of the data structure go to supplementary materials II. To obtain the intersection of two trees do:

```
t = t1 & t2
print(t)
```

```
<LocalTree Of Life | No record available: - n.count : 0- >
```

In this case, the intersection is empty because the Occurrences are overlaid in a regular lattice that partitions the space (i.e. the cells are disjoint). See supplementary materials II for a formal definition.

5.4.3. Efficient addition of trees from a list of cells

We can use the sum iteratively in a folding sum to obtain a Tree object representing all the areas defined in a list of Cells.

```
big_tree = reduce(lambda a , b : a+b , sample_trees)
```

However, this method is not efficient. In each step, a new tree is created and the internal logic to generate the union of all the intermediate nodes can result in redundant calculations. It is much faster to select first the occurrences for all the trees inside a list and then plug them into the Tree-Neo constructor, as in the example below.

```
# Faster version
ocs = map(lambda s : s.occurrences,sample_trees)
## ocs is a nested list.
## We need to flatten this into  a single list of occurrences
ocs = reduce(lambda a,b : a + b, ocs)
big_tree = TreeNeo(ocs)
print(big_tree)
```

```
<LocalTree Of Life | Root: LUCA - n.count : 374731- >
```

The resulting tree could be very large. In this case, the obtained tree (`big_tree`) comprises 374731 occurrences. Remember that this tree is the resulting union of all the local taxonomic trees obtained from the neighbourhood of degree 4 around the cells where jaguars occurred.

5.5. Selecting nodes from the Red List

We filter the *Species* nodes from the `big_tree` that are present in the Red List of threatened species. To do this we simply match the names using regular expressions. Using more sophisticated methods for data matching are out of the scope of the present example. We assume that the Red List data (a CSV file) have been loaded into a data frame with the name `redlist`.

```
## Filter critically endangered species
critical_sps = redlist[
                        (redlist.redlistCategory == 'Critically Endangered')
                       | (redlist.redlistCategory == 'Endangered')
                       | (redlist.redlistCategory == 'Vulnerable')
                     ].scientificName.apply(str.lower)


protected_by_jaguar = map(lambda critical_sp :
```

```
                                filter(lambda sp : critical_sp in sp.name.lower(),
                                big_tree.species),
                                critical_sps)

## Remove empty lists
protected_by_jaguar = filter(lambda l :
                                    l != [], protected_by_jaguar)
## flatten lists
threatened_species = reduce(lambda a,b : a + b ,protected_by_jaguar)
## remove species repetitions
threatened_species = list(set(threatened_species))
## Extract all corresponding occurrences and flatten list
t_ocs = reduce(lambda l1,l2 : l1 + l2 ,
                map(lambda l : l.occurrences, threatened_species))
## Instantiate new tree
threatened_tree = TreeNeo(t_ocs)
```

The `threatened_tree` is now a taxonomic tree that includes only the occurrences that match the species names of the Red List. To calculate the percentage of threatened species contained in the selected tree we can do:

```
## total number of critical endangered species
ncrit = len(critical_sps)
len(threatened_tree.species) / float(ncrit) * 100

13.49 %
```

That is, 13.49% of the threatened species are contained in the neighbouring regions where jaguars had been registered. To see if this result is relevant we calculate the percentage of the covered

area with respect to the whole country. Before doing so, it is convenient to transform the selected geometries in a projected coordinate system with metric units.

5.5.1. Reprojecting data

The default coordinate reference system (crs) in the data used is in geographic coordinates with WGS84 datum (EPSG:4326). The units of this crs is in degrees, therefore the calculated area is defined in squared degrees. In order to account for areas and distances in meters (or kilometers) we need to project the selected geometries into an appropriate projected coordinate system. To achieve this, we need to import some extra functions.

```
from shapely.ops import transform
from shapely import wkt,wkb
import pyproj
from functools import partial
```

Here we used the *Alberts Equal Area Conic projection* to account for an accurate area representation. This projection is specified in a string using the Proj4 syntax.

```
projection_string = """+proj=aea +lat_1=14.5 +lat_2=32.5 +lat_0=24
                    +lon_0=-105 +x_0=0 +y_0=0 +ellps=GRS80
                    +datum=NAD83 +units=m +no_defs;
                    """
mex_eq_area_proj = pyproj.Proj(projection_string)
## The WGS84 crs is defined as EPSG:4326
proj_in = pyproj.Proj(init='epsg:4326')
## function to project using the parameters of the
## original projection and the mexican equal area.
project = partial(
```

```
    pyproj.transform,
    proj_in,
    mex_eq_area_proj)
## Transform all cells to calculate area.
projected_neighbours_cells = map(lambda cell :
                                  transform(project,
                                  cell.polygon_shapely),
                                  neighbours)
```

For calculating the average cell size and the total area in square kilometers (1,000,000 m^2) we do:

```
tokm2 = 1000000 # to convert to sq. kilometers
areas = map(lambda cell : cell.area,
            projected_neighbours_cells)
total_cell_area = sum(areas)
## calculate the mean
np.mean(areas) / tokm2
## standard deviation
np.std(areas)/ tokm2
```

The calculated average area of all cells is 27 ± 3 km^2 and the total area is $8,509.81$ km^2.

5.6. *Trimming trees*

In certain situations we need to select a particular branch of a tree. We can cut (*trim*) this branch by simply selecting a node and converting it into a `TreeNeo` instance to produce a full feature tree. The method (function) for converting a TreeNode into a full feature tree is: `plantTreeNode`. We focus our attention on four branches of the `threatened` tree that co-occurs with the presence

of jaguars. These branches are: mammals (class *Mammalia*), parrots (order *Psittaciformes*) amphibians (class *Amphibia*) and plants (kingdom: Plantae).

5.6.1. *Select the branch of interest*

Trimming the tree is achieved by first selecting the nodes of interest and then converting all the descendant branches into fully featured trees. There is no restriction for selecting the taxonomic type of the node (`mammals` and `amphibians` are `Class` type while `parrots` are `Order` type).

```
mammals = threatened_tree.to_Animalia.to_Chordata.to_Mammalia
parrots = threatened_tree.to_Animalia.to_Chordata.to_Aves.to_Psittaciformes
amphibians = threatened_tree.to_Animalia.to_Chordata.to_Amphibia
plants = threatened_tree.to_Plantae
```

The method `plantTreeNode()` converts the `TreeNode` and resulting descendants into a full featured tree (TreeNeo object).

```
mammals = mammals.plantTreeNode()
birds = birds.plantTreeNode()
amphibians = amphibians.plantTreeNode()
plants = plants.plantTreeNode()
```

We can add all these trees together using the sum operation.

```
vertebrates = mammals + parrots + amphibians
```

However, as explained earlier, an optimized version for summing more than two trees is achieved by instantiating a `TreeNeo` with all the occurrences.

```
vertebrates = TreeNeo(mammals.occurrences +
                      parrots.occurrences +
                      amphibians.occurrences)
print(vertebrates)
```

The total number of occurrences contained in the vertebrates tree is:

```
<LocalTree Of Life | Root: LUCA - n.count : 2056- >
```

5.6.2. *Ranking the most frequent nodes in the selected list of cells*

We proceed now to rank some groups according to their frequency of occurrence within the cells of the study area (i.e. the jaguar's neighbouring cells). The ranking analysis calculates this frequency for each node in a tree given a referential list of trees. That is, assuming that we have *n* different trees (e.g. one per cell), and a tree of interest (in this case `threatened_tree`) how frequently each node appears in the global tree (e.g `threatened_trees`) with respect to the list of *n* trees? Figure 9 shows these frequencies visualised as the size of each node. In our implementation, this analysis is performed with the method: `countNodesFrequenciesOnList(list_of_trees)` That is:

```
vertebrates.countNodesFrequenciesOnList(list_of_trees=sample_trees)
mammals.countNodesFrequenciesOnList(list_of_trees=sample_trees)
parrots.countNodesFrequenciesOnList(list_of_trees=sample_trees)
amphibians.countNodesFrequenciesOnList(list_of_trees=sample_trees)
plants.countNodesFrequenciesOnList(list_of_trees=sample_trees)
```

We can therefore rank by taxonomic level. In this example we show the procedure for *family* and *species* level in the different branches. Here, we show the corresponding top five nodes.

```
mammals.rankLevels()
mammals.families[:5]
```

```
[<LocalTree | Family: Muridae - n.count : 8 | AF: 0.30>,
 <LocalTree | Family: Phyllostomidae - n.count : 8 | AF: 0.29>,
 <LocalTree | Family: Cervidae - n.count : 14 | AF: 0.16>,
```

```
 <LocalTree | Family: Heteromyidae - n.count : 3 | AF: 0.15>,
 <LocalTree | Family: Tayassuidae - n.count : 158
 | AF: 0.15>]
```

```
parrots.rankLevels()
parrots.species[:5]
```

```
[<LocalTree | Specie: Ara militaris (Linnaeus, 1766) - n.count : 27->,
 <LocalTree | Specie: Amazona finschi (P. L. Sclater, 1864) - n.count : 23- >,
 <LocalTree | Specie: Amazona auropalliata (Lesson, 1842) - n.count : 3- >,
 <LocalTree | Specie: Amazona oratrix Ridgway, 1887 - n.count : 2- >,
```

```
amphibians.rankLevels()
amphibians.families[:3]
```

```
[<LocalTree | Family: Hylidae - n.count : 128- | AF: 0.083>,
 <LocalTree | Family: Plethodontidae - n.count :
 160 | AF: 0.05>,
 <LocalTree | Family: Eleutherodactylidae -
 n.count : 1- | AF: 0.016>]
```

```
plants.rankLevels()
plants.genera[:3]
```

```
[<LocalTree | Genus: Tillandsia - n.count : 3- | AF: 0.2>,
 <LocalTree | Genus: Lonchocarpus - n.count : 5- | AF: 0.18>,
 <LocalTree | Genus: Eugenia - n.count : 1- | AF: 0.15>]
```

5.7. Associated raster (environmental) information

Here, we demonstrate how to access raster data associated with a taxonomic tree `TreeNeo`. The raster data used are related to environmental variables stored in the RGU. Currently there are two

forms for accessing this information: *i)* as a table with columns corresponding to environmental variables and rows defined by each occurrence (a point-based method); *ii)* as a raster object sampled from the associated geometry of each tree or, in general, any (multi) polygon object. The raster object features methods for visualisation, geoprocessing and data exchange.

5.7.1. Extracting raster information as table

To extract the data in this format use the method (function):

```
TreeNeo.associatedData.getEnvironmentalVariablesPoints()
```

The output is a *Pandas* dataframe with the associated values of climatic covariates. See the following example:

```
table = vertebrates.associatedData.getEnvironmentalVariablesPoints()
print(table[:1])
```

Here we only show the first record.

Table 2: Output for environmental variables. Here showing only mean values for some variables on a single record.

	MinTemperature	...	Precipitation	Vapor	SolarRadiation	WindSpeed
0	22.25	...	21.16	1.33	16466.25	2.33

The geometric object of each tree is determined by the `Occurrence` nodes of the tree. In the graph database, each `Occurrence` node is linked to the `Cell` node that geographically contains the occurrence's location. One of the attributes of the `Cell` object is the geographic polygon that defines its border. The union of all the corresponding `Cell` nodes is what determines the geometric feature of the tree `TreeNeo`. As such, the raster extraction process is performed on each of the tree's associated cells.

5.7.2. Extracting Raster objects from TreeNeo instances

To extract the associated raster object of a `TreeNeo` instance use the method (function):

```
TreeNeo.associatedData.getAssociatedRasterAreaData([name of variable])
```

To obtain several environmental variables use: `associatedData.getEnvironmentalVariablesCells()`

For example, information for a single variable can be obtained with:

```
meantemp_data = vertebrates.associatedData.
                                 getAssociatedRasterAreaData(
                                 'MeanTemperature')
```

The raster object is automatically added to the `TreeNeo` object after the method is called. The raster objects are appended to the attribute `associatedData`.

5.8. Extracting raster objects from arbitrary polygons

The extraction of raster objects is performed by the `raster_api` library, a Biospytial module for reading, writing and processing raster objects using the RGU as backend.

The `raster_api` can use natively any object stored in the knowledge engine that has at least a two dimensional geometric feature (attribute). This includes the basic operations for querying, reading and writing. For using external geometric objects like *Shapefiles, GeoPackages, GeoJSON, etc* the objects need to be transformed to their corresponding WKT or WKB (*Well Known Binary*) representation. Examples of these are described extensively in the Jupyter notebooks and in the documentation.

In this example we use the polygon defined by the border of Mexico to extract several raster objects (RasterData instances) using the *raster_api* module. We use these objects to compare the environmental ranges of: the threatened species, the Jaguars' habitat and the entire area of the country to conclude if the environmental niche of the threated species are covered by the habitat of the Jaguars' and how these ranges are different with respect to the whole country.

5.8.1. *Importing the polygon for Mexico*

The first step in this is to import the polygon for Mexico. The default installation of Biospytial includes the WorldBorders dataset (https://thematicmapping.org). Assuming that this dataset is installed, we can import the polygon of Mexico with the API provided by the class `Country` located in `sketches.models`. Country is a vector dataset stored in the RDBMS. The geometric feature is stored as the `geom` column.

```
from sketches.models import Country
## The syntax follows the Django Query Set API
mexico = Country.objects.filter(name='Mexico').first()
mex_area = mexico.geom.area

## For reprojecting the area of Mexico we similarly do:
mex_shapely = wkt.loads(mexico.geom.wkt)
mex_projected= transform(project,mex_shapely)
```

To calculate the percentage of area covered by all the cells with respect with the total area of Mexico we can do:

```
total_cell_area / mex_projected.area * 100

3.42%
```

For example, we can display simple visualisations invoking the method: `display_field()`. See figure 7.

```
vertebrates.associatedData.raster_MeanTemperature.display_field()
```

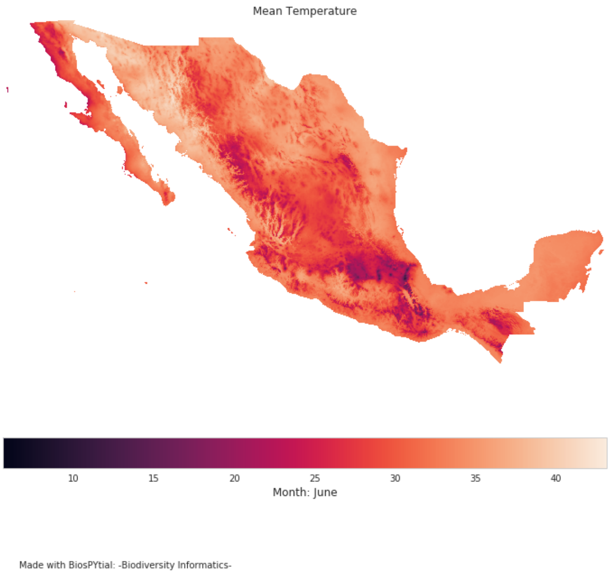

Figure 7: The output of the method: `display_field()`, an easy way to visualise RasterData objects.

5.8.2. *Interactive visualisation*

As an alternative, we can export the raster object as an *xarray* (http://xarray.pydata.org) instance for interactive visualisation using the *Geoviews* (http://geoviews.org) package. To export the associated raster data to an *xarray* object do:

```
meantemp = vertebrates.associatedData.raster_MeanTemperature.to_xarray()
```

The following code gives an example on how to generate an interactive visualisation using the vertebrates' associated mean temperature data and the locations of the observed threatened species associated with the presence of Jaguars. We used the elevation data for Mexico (extracted before) as basemap. Figure 8 shows this visualisation at two different scales.

```
import geoviews as gv
from cartopy import crs
import geoviews.feature as gf
from geoviews import opts
gv.extension('bokeh')


sample_pt = gv.Points((env_threated_occurrences.x,env_threated_occurrences.y),
```

```
            label='ocurrences').opts(
            fill_color = 'orange',
            line_color = 'black',
            line_width = 0.5,
            line_alpha = 0.4,
            fill_alpha = 1.0,
            size = 5,
            )

elevation = all_mex_datasets[0].to_xarray()
elevds = gv.Dataset(elevation,crs=crs.PlateCarree())
elevimg = gvds.to(gv.Image,['Longitude','Latitude']
                  ).opts(cmap=plt.cm.gist_earth)

temp = meantemp.where(((meantemp.Longitude > -95) &
                       (meantemp.Longitude < -89) &
                       (meantemp.Latitude > 15) &
                       (meantemp.Latitude < 19)),
                       drop=True)

temp.name = meantemp.name
tempds = gv.Dataset(temp,crs=crs.PlateCarree())
tempimg = tempds.to(gv.Image,['Longitude','Latitude']).opts(cmap=plt.cm.magma)
## Display the map
map_ = (elevimg * gf.ocean * gf.coastline * gf.borders * tempimg * sample_pt )
```

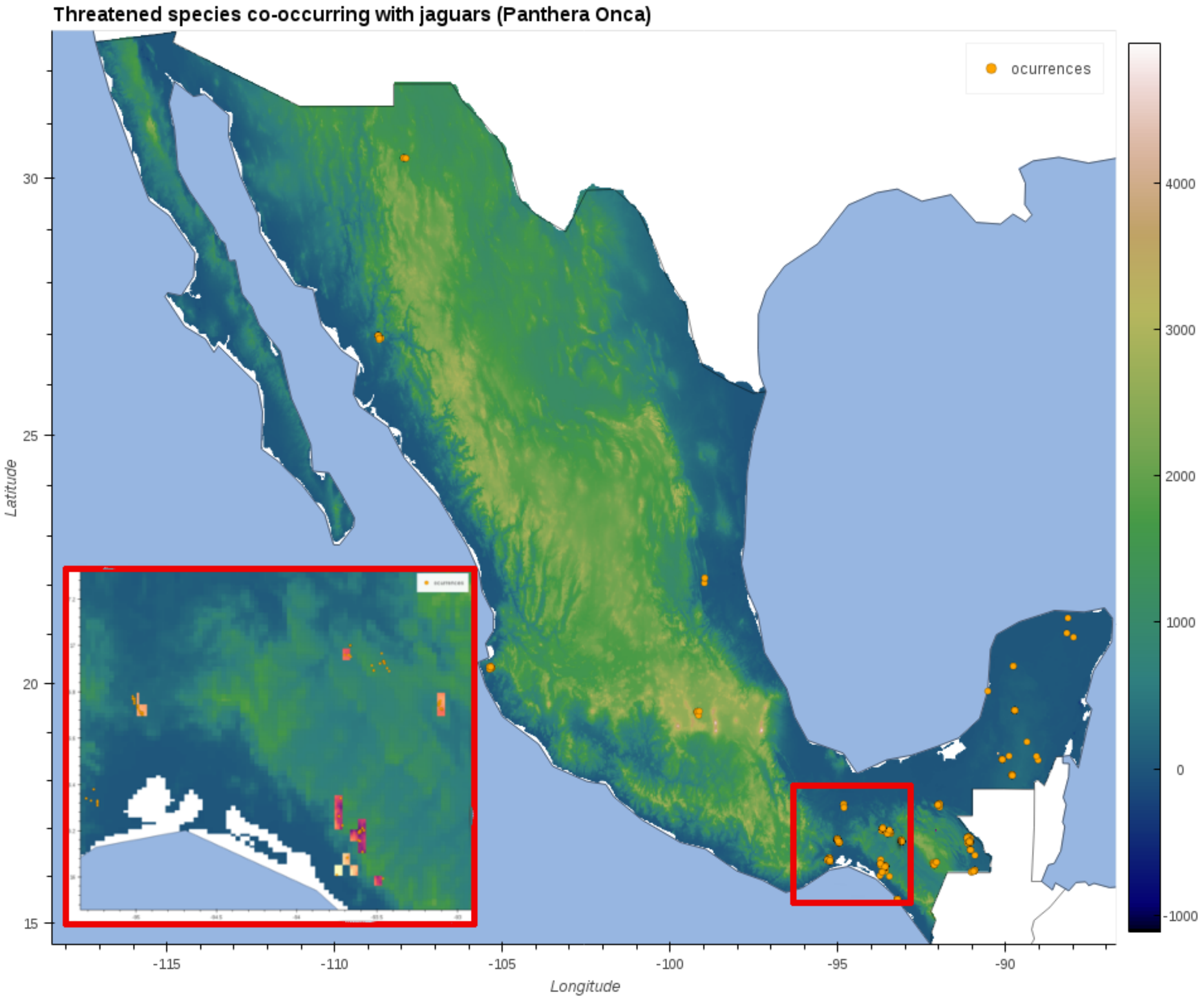

Figure 8: A composite figure showing two states of the interactive visualisation. Orange dots represent occurrences of threated species associated with the presence of jaguars (*P. Onca*). The inland red square shows the zoomed-in area depicted in the left side of the figure. The colored squares in the zoomed area shows the mean temperature associated with threatened vertebrates (phylum Chordata). The base map shows the elevation for all the country. See section 3.2 for information regarding the data used.

5.9. *Network visualisation and analysis*

Each *tree* instance induces an acyclic graph. We can convert the tree into a networkx object to visualise and analyse its network properties. To do this, we simply need to use the method: `tree.toNetworkx(depth_level=[k])` where k is the taxonomic level to reach in the tree, 0 for root 7 for species level.

5.9.1. *Visualisation*

A method for interactive visualisation has been developed using the *Holoviews* (`https://holoviews.org`) framework. To do this we need to invoke the method:

```
## Plot the Tree
from drivers.tools import to_interactivePlot
network = to_interactivePlot(threatened_tree,label_depth=8)
```

The output is a dictionary with two key-items: one for labels and the other for the actual graph (nodes and edges). To plot the whole graph we need to overlay both items.

```
network['labels'] * network['graph']
```

5.9.2. *Analysis with standard graph algorithms*

The `TreeNeo` structures are particular cases of graph traversals. As such, they can be analysed with graph theoretic methods. The library NetworkX (`https://networkx.github.io/`) is a Python package designed for analysing structure, dynamics and functions of complex networks. It includes standard graph algorithms and analysis measures as well as tools for import and export to other standard formats. We can convert a `TreeNeo` using the method: `toNetworkx(depth_level).` where depth_level is the depth of the graph to be generated. In the next example we convert the threatened_tree to a `NetworkX` object and use this to calculate its corresponding adjacency matrix.

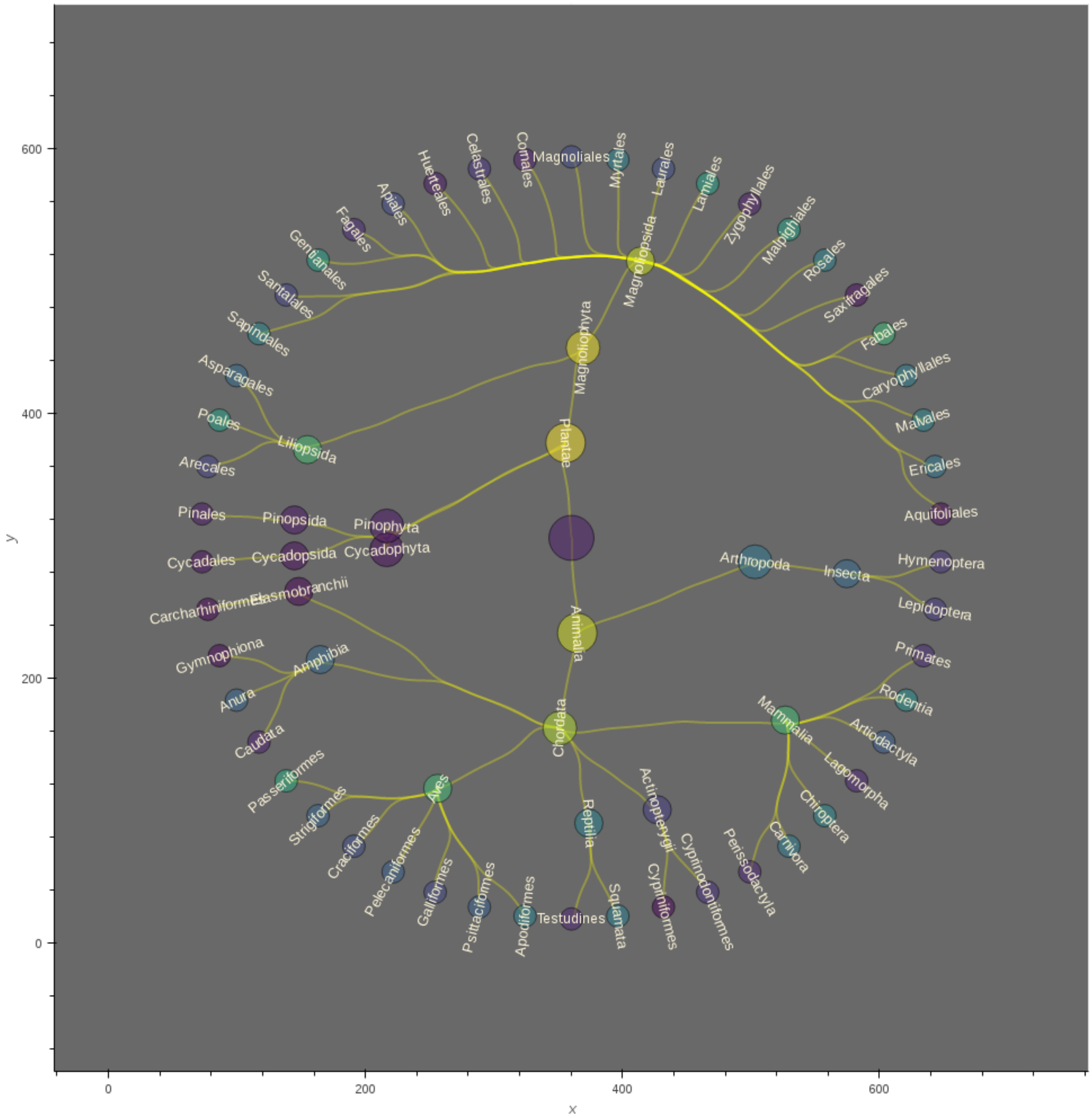

Figure 9: A tree visualization for the merged tree corresponding to threatened taxa, showing up to *Order* level. The size of the nodes is proportional to the taxonomic level (the largest is the root of the tree, the smallest are orders). The node colouring indicates the frequency of occurrence with respect to all the neighbouring cells (neighbours of jaguars) being the brightest the highest ranked and the darker the lowest ranked.

```
threatened_graph = threatened_tree.toNetworkx(depth_level=7)
from networkx import adjacency_matrix
M = adjacency_matrix(threatened_graph)
# uncomment this to plot the matrix
#plt.imshow(M.todense())
```

Representing `TreeNeo` objects into NetworkX graphs brings new possibilities for analysis and modelling. We hope this example will awake the spirit of the reader to explore the potential of representing data as complex graph structures.

6. Conclusions

Biospytial uses open source standards to integrate geospatial ecological big data as a tool for ecological niche modelling and the analysis of species distributions. This integration creates a complex network of data with enormous potential for data mining, information retrieval and visualisation. At the core, a web of semantic-wise relationships constitutes a corpus of taxonomic and environmental knowledge that opens up new ways to query and unveil complex ecological relations. To our knowledge, there is no other Open Source system with the design and capacity of achieving this including: i) storing information in a hybrid relational-graph system and ii) performing geospatial processes in vector and raster scalable databases.

A practical example provided a glimpse into how to query and manipulate taxonomic tree structures, as well as how to extract data, conduct frequency analysis and visualise results. The example demonstrated a new procedure to rank co-occurring taxonomic groups in an arbitrary size neighbourhood of pixels.

The GBIF occurrence data includes information only on location and taxonomy and in this sense the data are limited. However, the engine's design allows the capture, extension and exploration of semantic interpretation of the data by adding other types of relations. For example, linking information on trophic networks to the taxonomic backbone can help in analysing spatial patterns of trophic groups and dependant species, a key question in conservation biology.

The development of Biospytial has followed best practices in scientific programming (Wilson et al., 2014a). We recognise that spatial analyses are often not generalisable and therefore replicable. However replicability and reproducibility can be enhanced by increasing openness and documentation transparency and completeness (Barba, 2019; Teytelman, 2018; Shannon and Walker, 2018). In fact, Biospytial's source code is open and can be accessed at: `https://github.com/molgor/biospytial.git` while this manuscript is Open Access. In the future, Biospytial can be further developed into a system not only for integration and distribution of datasets, but also as a

tool for collaboration, experimentation, validation and reproduction of results in the era of Open Science, satisfying also the requisites of second generation SDI.

7. Availability of supporting source code and requirements

- Project name: Biospytial
- Project home page: `https://github.com/molgor/biospytial`
- Operating System(s): Platform independent (not tested in Windows)
- Other requirements: Docker 1.13 or higher
- License: GNU General Public License version 3.0 (GPLv3)
- Memory requirements: 40GB in HD for installing the database and at least 16GB in RAM for running the example.

The current example is located inside the folder `examples` with the name: `[Official Demo] Co-occurrences_Jaguar.ipynb`. The example has been modified only in the neighbourhood order, changing from 4 to 1. This modification reduces the data to process and the executing time.

8. Availability of supporting data

Snapshots of the databases need to be downloaded for running the software and example. The data are compressed in a single file (13GB) located in: `https://ecomorphs.ams3.digitaloceanspaces.com/biospytial/biospytial-data-1.0.tar.gz` . The container images are located in the public *Docker Hub* registry. The images can be downloaded automatically using the `installEngine.sh` Instructions for installing and running the engine are located in the project's homepage.

Table 3: Corresponding URLs for source code and container images for the Biospytial engine. The modules and the source code do not include data. These should be installed separately or loaded independently.

**Module name**	**URL**
Graph Storage and Processing Unit	`https://hub.docker.com/r/molgor/postgis_biospytial`
Biospytial Computing Engine	`https://hub.docker.com/r/molgor/biospytial`
Relational Geoprocessing Unit	`https://hub.docker.com/r/molgor/neo4j_biospytial`
Source code	`https://github.com/molgor/biospytial`
Data	biospytial-data-1.0.tar.gz

9. Funding

This project was jointly sponsored by the Doctoral Scholarships Program from the Mexican Science and Technology Council (CONACYT), the Faculty of Science and Technology from Lancaster University (FST-LU) and the GBIF Consortium through the GBIF Young Researchers Award (2016).

10. Authors' contributions

J.E. and P.A. conceived the original idea, which was further refined by all authors. The semantic structures and graph traversals were designed by J.E. with the mentorship of L.S. for integrating datasets. The software and system's design was developed by J.E. under the supervison of P.A. and L.S. The writing of the original draft was done by J.E with reviewing and editing from P.A. and L.S.

11. Competing interests

The authors declare that they have no competing interests.

12. Acknowledgments

We thank the effort of many researchers, students, public servants and citizen scientists that had contributed to sample, register and curate all the biodiversity occurrences data contained in the GBIF database. We want to thank specially Raúl Jiménez Rosenberg from Conabio for facilitating a complete snapshot of the GBIF database (2016) and the Free and Open Source Software community whose effort in developing software made possible the creation of this software.

13. References

Django [Computer Software], 2018. URL https://djangoproject.com.

Mehmet Altinel, Mehmet Altinel, Qiong Luo, Sailesh Krishnamurthy, C. Mohan, and Hamid Pirahesh. Dbcache: Database caching for web application servers. *SIGMOD*, 2002:612, 2002. URL http://citeseerx.ist.psu.edu/viewdoc/summary?doi=10.1.1.104.8991.

C. Amante and B.W. Eakins. ETOPO1 1 Arc-Minute Global Relief Model: Procedures, Data Sources and Analysis. Technical Report March, jan 2009. URL https://data.nodc.noaa.gov/cgi-bin/iso?id=gov.noaa.ngdc.mgg.dem:316http://www.ngdc.noaa.gov/mgg/global/global.html.

ANACONDA. vers. 2-2.4.0, Anaconda Software Distribution. Computer software, 2016. URL https://anaconda.com.

S. J. Andelman and W. F. Fagan. Umbrellas and flagships: Efficient conservation surrogates or expensive mistakes? *Proceedings of the National Academy of Sciences*, 97(11):5954–5959, 2000. ISSN 0027-8424. doi: 10.1073/pnas.100126797.

Lorena A. Barba. Praxis of Reproducible Computational Science. *Computing in Science and Engineering*, 21(1):73–78, 2019. ISSN 1558366X. doi: 10.1109/MCSE.2018.2881905.

Julian Besag. Spatial Interaction and the Statistical Analysis of Lattice Systems. *Journal of the Royal Statistical Society. Series B (Methodological)*, 36(2):192–236, 1974. ISSN 00359246. URL http://www.jstor.org/stable/2984812.

Julian Besag, Jeremy York, and Annie Mollié. Bayesian image restoration, with two applications in spatial statistics. *Annals of the Institute of Statistical Mathematics*, 43(1):1–20, mar 1991. ISSN 00203157. doi: 10.1007/BF00116466. URL http://link.springer.com/10.1007/BF00116466.

R E Blackwelder. *Taxonomy: a text and reference book*. Wiley, 1967.

Elizabeth T. Borer, W. Stanley Harpole, Peter B. Adler, Eric M. Lind, John L. Orrock, Eric W. Seabloom, and Melinda D. Smith. Finding generality in ecology: A model for globally distributed experiments. *Methods in Ecology and Evolution*, 5(1):65–73, 2014. ISSN 2041210X. doi: 10.1111/2041-210X.12125.

E. S. Brondizio, J. Settele, S. Díaz, H. T. Ngo, and (editors). IPBES. 2019 Global assessment report on biodiversity and ecosystem services of the Intergovernmental Science- Policy Platform on Biodiversity and Ecosystem Services. Technical report, Bonn, Germany, 2019. URL `https://www.ipbes.net/global-assessment-biodiversity-ecosystem-services`.

Joe Celko. *Graph Databases*. 2014. ISBN 9780124071926. doi: 10.1016/B978-0-12-407192-6.00003-0. URL `http://dx.doi.org/10.1016/B978-0-12-407192-6.00003-0`.

Min Chen, Shiwen Mao, and Yunhao Liu. Big data: A survey. In *Mobile Networks and Applications*, 2014. doi: 10.1007/s11036-013-0489-0.

Eliseo Clementini, Paolino Felice, and Peter Oosterom. A small set of formal topological relationships suitable for end-user interaction. pages 277–295. Springer, Berlin, Heidelberg, 1993. doi: 10.1007/3-540-56869-7_16. URL `http://link.springer.com/10.1007/3-540-56869-7{_}16`.

PROJ contributors. PROJ coordinate transformation software library, 2019. URL `https://proj4.org/`.

J. Antonio de la Torre, Juan Manuel Núñez, and Rodrigo A. Medellín. Spatial requirements of jaguars and pumas in Southern Mexico. *Mammalian Biology*, 84:52–60, 2017. ISSN 16181476. doi: 10.1016/j.mambio.2017.01.006.

P. J. Diggle, J. A. Tawn, and R. A. Moyeed. Model-based geostatistics. *Journal of the Royal Statistical Society: Series C (Applied Statistics)*, 47(3):299–350, jan 2002. ISSN 00359254. doi: 10.1111/1467-9876.00113. URL `http://doi.wiley.com/10.1111/1467-9876.00113`.

T Dobzhansky and T G Dobzhansky. *Genetics of the Evolutionary Process*. Columbia University Press, 1970. ISBN 9780231083065.

Docker Inc. Enterprise Application Container Platform | Docker, 2019. URL `https://www.docker.com/`.

C. Ronnie Drever, Chantal Hutchison, Mark C. Drever, Daniel Fortin, Cheryl Ann Johnson, and Yolanda F. Wiersma. Conservation through co-occurrence: Woodland caribou as a focal species for boreal biodiversity. *Biological Conservation*, 232(January):238–252, 2019. ISSN 00063207. doi: 10.1016/j.biocon.2019.01.026. URL `https://doi.org/10.1016/j.biocon.2019.01.026`.

Max J. Egenhofer and Robert D. Franzosa. Point-set topological spatial relations. *International Journal of Geographical Information Systems*, 5(2):161–174, jan 1991. ISSN 02693798. doi: 10.1080/02693799108927841. URL `http://www.tandfonline.com/doi/abs/10.1080/02693799108927841`.

Brian J Enquist, Richard Rick Condit, Robert K Peet, Mark Schildhauer, and Barbara M. Thiers. The Botanical Information and Ecology Network (BIEN): Cyberinfrastructure for an integrated botanical information network to investigate the ecological impacts of global climate change on plant biodiversity. *PeerJ*, 2016. ISSN 2167-9843. doi: 10.7287/peerj.preprints.2615v2.

European Space Agency. Copernicus, 2014. URL `https://www.esa.int/Our{_}Activities/Observing{_}the{_}Earth/Copernicus/Overview3http://www.esa.int/Our{_}Activities/Observing{_}the{_}Earth/Copernicus/Overview4`.

Antonio Fabregat, Florian Korninger, Guilherme Viteri, Konstantinos Sidiropoulos, Pablo Marin-

Garcia, Peipei Ping, Guanming Wu, Lincoln Stein, Peter D'Eustachio, and Henning Hermjakob. Reactome graph database: Efficient access to complex pathway data. *PLOS Computational Biology*, 14(1):e1005968, jan 2018. ISSN 1553-7358. doi: 10.1371/journal.pcbi.1005968. URL http://dx.plos.org/10.1371/journal.pcbi.1005968.

S.E Fick and R.J Hijmans. Worldclim 2: New 1-km spatial resolution climate surfaces for global land areas. *International Journal of Climatology*, may 2017. ISSN 08998418. doi: 10.1002/joc.5086. URL http://doi.wiley.com/10.1002/joc.5086.

John Gantz and David Reinsel. Extracting Value from Chaos. Technical report, 2011.

GBIF Secretariat. Global Biodiversity Infrastructure, 2015. URL http://www.gbif.org/participation/participant-list.

GBIF Secretariat. GBIF Backbone Taxonomy, 2017. URL ttps://doi.org/10.15468/39omeiaccessedviaGBIF.org.

GDAL/OGR Contributors. GDAL/OGR - Geospatial Data Abstraction software Library, 2018. URL https://www.gdal.org/.

Geometry Engine Open Source (Contributors). Geometry Engine Open Source, 2019. URL https://trac.osgeo.org/geos.

Michael F. Goodchild. Citizens as sensors: the world of volunteered geography. *GeoJournal*, 69 (4):211–221, nov 2007. ISSN 0343-2521. doi: 10.1007/s10708-007-9111-y. URL http://link.springer.com/10.1007/s10708-007-9111-y.

Martin Grund, Philippe Cudre-Mauroux, Jens Krueger, and Hasso Plattner. Hybrid graph and relational query processing in main memory. In *Proceedings - International Conference on Data Engineering*, pages 23–24, 2013. ISBN 9781467353021. doi: 10.1109/ICDEW.2013.6547419.

Aric A. Hagberg, Daniel A. Schult, and Pieter J. Swart. Exploring Network Structure, Dynamics, and Function using NetworkX. In G Varoquaux, T Vaught, and J Millman, editors, *Proceedings of the 7th Python in Science conference (SciPy 2008)*, pages 11–15, 2008. URL http://conference.scipy.org/proceedings/SciPy2008/paper{_}2/.

Jan L. Harrington. *Relational Database Design and Implementation.* 2009. ISBN 9780123747303. doi: 10.1016/B978-0-12-374730-3.X0001-0. URL https://www.sciencedirect.com/book/9780128043998/relational-database-design-and-implementation.

Florian Hartig, James Dyke, Thomas Hickler, Steven I. Higgins, Robert B. O'Hara, Simon Scheiter, and Andreas Huth. Connecting dynamic vegetation models to data - an inverse perspective. *Journal of Biogeography*, 39(12):2240–2252, 2012. ISSN 03050270. doi: 10.1111/j.1365-2699.2012.02745.x.

Christian Heipke. Crowdsourcing geospatial data. *ISPRS Journal of Photogrammetry and Remote Sensing*, 65(6):550–557, nov 2010. ISSN 09242716. doi: 10.1016/j.isprsjprs.2010.06.005. URL http://www.sciencedirect.com/science/article/pii/S0924271610000602.

Paul H.J. Hendriks, Ezra Dessers, and Geert van Hootegem. Reconsidering the definition of a spatial data infrastructure. *International Journal of Geographical Information Science*, 26(8):1479–1494, 2012. ISSN 13658816. doi: 10.1080/13658816.2011.639301.

John R. Herrig. Simple Feature Access - Part 1: Common Architecture | OGC. Technical report, Open Geospatial Consortium Inc., 2011. URL http://www.opengeospatial.org/standards/sfa.

Martin Hilbert and Priscila López. The world's technological capacity to store, communicate, and compute information. *Science (New York, N.Y.)*, 332(6025):60–5, apr 2011. ISSN 1095-9203. doi: 10.1126/science.1200970. URL http://www.ncbi.nlm.nih.gov/pubmed/21310967.

Kurt Hornik. The Comprehensive R Archive Network, 2012. ISSN 19395108.

Paul Hudak and Paul. Conception, evolution, and application of functional programming languages. *ACM Computing Surveys*, 21(3):359–411, sep 1989. ISSN 03600300. doi: 10.1145/72551.72554. URL `http://portal.acm.org/citation.cfm?doid=72551.72554`.

Lawrence N. Hudson, Tim Newbold, Sara Contu, Samantha L.L. Hill, Igor Lysenko, Adriana De Palma, Helen R.P. Phillips, Rebecca A. Senior, Dominic J. Bennett, Hollie Booth, Argyrios Choimes, David L.P. Correia, Julie Day, Susy Echeverría-Londoño, Morgan Garon, Michelle L.K. Harrison, Daniel J. Ingram, Martin Jung, Victoria Kemp, Lucinda Kirkpatrick, Callum D. Martin, Yuan Pan, Hannah J. White, Job Aben, Stefan Abrahamczyk, Gilbert B. Adum, Virginia Aguilar-Barquero, Marcelo A. Aizen, Marc Ancrenaz, Enrique Arbeláez-Cortés, Inge Armbrecht, Badrul Azhar, Adrián B. Azpiroz, Lander Baeten, András Báldi, John E. Banks, Jos Barlow, Péter Batáry, Adam J. Bates, Erin M. Bayne, Pedro Beja, Åke Berg, Nicholas J. Berry, Jake E. Bicknell, Jochen H. Bihn, Katrin Böhning-Gaese, Teun Boekhout, Céline Boutin, Jérémy Bouyer, Francis Q. Brearley, Isabel Brito, Jörg Brunet, Grzegorz Buczkowski, Erika Buscardo, Jimmy Cabra-García, María Calviño-Cancela, Sydney A. Cameron, Eliana M. Cancello, Tiago F. Carrijo, Anelena L. Carvalho, Helena Castro, Alejandro A. Castro-Luna, Rolando Cerda, Alexis Cerezo, Matthieu Chauvat, Frank M. Clarke, Daniel F.R. Cleary, Stuart P. Connop, Biagio D'Aniello, Pedro Giovâni da Silva, Ben Darvill, Jens Dauber, Alain Dejean, Tim Diekötter, Yamileth Dominguez-Haydar, Carsten F. Dormann, Bertrand Dumont, Simon G. Dures, Mats Dynesius, Lars Edenius, Zoltán Elek, Martin H. Entling, Nina Farwig, Tom M. Fayle, Antonio Felicioli, Annika M. Felton, Gentile F. Ficetola, Bruno K.C. Filgueiras, Steven J. Fonte, Lauchlan H. Fraser, Daisuke Fukuda, Dario Furlani, Jörg U. Ganzhorn, Jenni G. Garden, Carla Gheler-Costa, Paolo Giordani, Simonetta Giordano, Marco S. Gottschalk, Dave Goulson, Aaron D. Gove, James Grogan, Mick E. Hanley, Thor Hanson, Nor R. Hashim, Joseph E. Hawes, Christian Hébert, Alvin J. Helden, John André Henden, Lionel Hernández, Felix Herzog, Diego Higuera-Diaz, Branko Hilje, Finbarr G.

Horgan, Roland Horváth, Kristoffer Hylander, Paola Isaacs-Cubides, Masahiro Ishitani, Carmen T. Jacobs, Víctor J. Jaramillo, Birgit Jauker, Mats Jonsell, Thomas S. Jung, Vena Kapoor, Vassiliki Kati, Eric Katovai, Michael Kessler, Eva Knop, Annette Kolb, Ádám Korösi, Thibault Lachat, Victoria Lantschner, Violette Le Féon, Gretchen Lebuhn, Jean Philippe Légaré, Susan G. Letcher, Nick A. Littlewood, Carlos A. López-Quintero, Mounir Louhaichi, Gabor L. Lövei, Manuel Esteban Lucas-Borja, Victor H. Luja, Kaoru Maeto, Tibor Magura, Neil Aldrin Mallari, Erika Marin-Spiotta, E. J.P. Marshall, Eliana Martínez, Margaret M. Mayfield, Grzegorz Mikusinski, Jeffrey C. Milder, James R. Miller, Carolina L. Morales, Mary N. Muchane, Muchai Muchane, Robin Naidoo, Akihiro Nakamura, Shoji Naoe, Guiomar Nates-Parra, Dario A. Navarrete Gutierrez, Eike L. Neuschulz, Norbertas Noreika, Olivia Norfolk, Jorge Ari Noriega, Nicole M. Nöske, Niall O'Dea, William Oduro, Caleb Ofori-Boateng, Chris O. Oke, Lynne M. Osgathorpe, Juan Paritsis, Alejandro Parra-H, Nicolás Pelegrin, Carlos A. Peres, Anna S. Persson, Theodora Petanidou, Ben Phalan, T. Keith Philips, Katja Poveda, Eileen F. Power, Steven J. Presley, Vânia Proença, Marino Quaranta, Carolina Quintero, Nicola A. Redpath-Downing, J. Leighton Reid, Yana T. Reis, Danilo B. Ribeiro, Barbara A. Richardson, Michael J. Richardson, Carolina A. Robles, Jörg Römbke, Luz Piedad Romero-Duque, Loreta Rosselli, Stephen J. Rossiter, T'ai H. Roulston, Laurent Rousseau, Jonathan P. Sadler, Szabolcs Sáfián, Romeo A. Saldaña-Vázquez, Ulrika Samnegård, Christof Schüepp, Oliver Schweiger, Jodi L. Sedlock, Ghazala Shahabuddin, Douglas Sheil, Fernando A.B. Silva, Eleanor M. Slade, Allan H. Smith-Pardo, Navjot S. Sodhi, Eduardo J. Somarriba, Ramón A. Sosa, Jane C. Stout, Matthew J. Struebig, Yik Hei Sung, Caragh G. Threlfall, Rebecca Tonietto, Béla Tóthmérész, Teja Tscharntke, Edgar C. Turner, Jason M. Tylianakis, Adam J. Vanbergen, Kiril Vassilev, Hans A.F. Verboven, Carlos H. Vergara, Pablo M. Vergara, Jort Verhulst, Tony R. Walker, Yanping Wang, James I. Watling, Konstans Wells, Christopher D. Williams, Michael R. Willig, John C.Z. Woinarski, Jan H.D. Wolf, Ben A. Woodcock, Douglas W. Yu, Andrey S. Zaitsev, Ben Collen, Rob M. Ewers, Georgina M. Mace, Drew W. Purves, Jörn P.W. Scharlemann,

and Andy Purvis. The PREDICTS database: A global database of how local terrestrial biodiversity responds to human impacts. *Ecology and Evolution*, 4(24):4701–4735, 2014. ISSN 20457758. doi: 10.1002/ece3.1303.

IUCN. The IUCN Red List of Threatened Species. Version 2013.2. *International Union for Conservation of Nature*, page Available at http://www.iucnredlist.org, 2019. URL `http://www.iucnredlist.org`.

Josh Juneau. Object-Relational Mapping. In *Java EE 8 Recipes*, pages 395–439. Apress, Berkeley, CA, 2018. doi: 10.1007/978-1-4842-3594-2_8. URL `http://link.springer.com/10.1007/978-1-4842-3594-2{_}8`.

Maged N Kamel Boulos, Bernd Resch, David N Crowley, John G Breslin, Gunho Sohn, Russ Burtner, William A Pike, Eduardo Jezierski, and Kuo-Yu Chuang. Crowdsourcing, citizen sensing and sensor web technologies for public and environmental health surveillance and crisis management: trends, OGC standards and application examples. *International Journal of Health Geographics*, 10(1):67, 2011. ISSN 1476-072X. doi: 10.1186/1476-072X-10-67. URL `http://ij-healthgeographics.biomedcentral.com/articles/10.1186/1476-072X-10-67`.

Jens Kattge, Sandra Diaz, Sandra Lavorel, I C Prentice, Paul Leadley, Gerhard Bönisch, Eric Garnier, Mark Westoby, Peter B Reich, I J Wright, and Others. TRY–a global database of plant traits. *Global change biology*, 17(9):2905–2935, 2011.

Steve Kelling, Daniel Fink, Frank A. La Sorte, Alison Johnston, Nicholas E. Bruns, and Wesley M. Hochachka. Taking a Big Data' approach to data quality in a citizen science project. *Ambio*, 2015. ISSN 16547209. doi: 10.1007/s13280-015-0710-4.

Karen Kemp and Muki Haklay. Open Source Geospatial Foundation (OSGF). In *Encyclopedia of Geographic Information Science*. 2014. doi: 10.4135/9781412953962.n153.

W. Daniel Kissling, Jorge A. Ahumada, Anne Bowser, Miguel Fernandez, Néstor Fernández, Enrique Alonso García, Robert P. Guralnick, Nick J.B. Isaac, Steve Kelling, Wouter Los, Louise McRae, Jean Baptiste Mihoub, Matthias Obst, Monica Santamaria, Andrew K. Skidmore, Kristen J. Williams, Donat Agosti, Daniel Amariles, Christos Arvanitidis, Lucy Bastin, Francesca De Leo, Willi Egloff, Jane Elith, Donald Hobern, David Martin, Henrique M. Pereira, Graziano Pesole, Johannes Peterseil, Hannu Saarenmaa, Dmitry Schigel, Dirk S. Schmeller, Nicola Segata, Eren Turak, Paul F. Uhlir, Brian Wee, and Alex R. Hardisty. Building essential biodiversity variables (EBVs) of species distribution and abundance at a global scale. *Biological Reviews*, 93(1): 600–625, 2018. ISSN 1469185X. doi: 10.1111/brv.12359.

Thomas Kluyver, Benjamin Ragan-Kelley, Fernando Pérez, Brian Granger, Matthias Bussonnier, Jonathan Frederic, Kyle Kelley, Jessica Hamrick, Jason Grout, Sylvain Corlay, Paul Ivanov, Damián Avila, Safia Abdalla, Carol Willing, and Jupyter Development Team. Jupyter Notebooks a publishing format for reproducible computational workflows. In *Positioning and Power in Academic Publishing: Players, Agents and Agendas*, pages 87 – 90. 2016. ISBN 9781614996491. doi: 10.3233/978-1-61499-649-1-87. URL `http://ebooks.iospress.nl/publication/42900`.

Julia Koricheva, Jessica Gurevitch, and Kerrie L. Mengersen. *Handbook of meta-analysis in ecology and evolution.* Princeton University Press, 2013. ISBN 9781400846184.

Ray Kurzweil. The Law of Accelerating Returns. In *Alan Turing: Life and Legacy of a Great Thinker*, pages 381–416. Springer Berlin Heidelberg, Berlin, Heidelberg, 2004. doi: 10.1007/978-3-662-05642-4_16. URL `http://link.springer.com/10.1007/978-3-662-05642-4{_}16`.

John La Salle, Kristen J. Williams, and Craig Moritz. Biodiversity analysis in the digital era. *Philosophical Transactions of the Royal Society B: Biological Sciences*, 2016. ISSN 14712970. doi: 10.1098/rstb.2015.0337.

L.A. Skornyakov (originator). Partially ordered set. *Encyclopedia of Mathematics*, October, 2014. URL http://www.encyclopediaofmath.org/index.php?title=Partially{_}ordered{_}set{&}oldid=33633.

Redis Labs. Redis, an in-memory data structure store, 2012. URL http://redis.io/.

Songnian Li, Suzana Dragicevic, Francesc Antón Castro, Monika Sester, Stephan Winter, Arzu Coltekin, Christopher Pettit, Bin Jiang, James Haworth, Alfred Stein, and Tao Cheng. Geospatial big data handling theory and methods: A review and research challenges. *ISPRS Journal of Photogrammetry and Remote Sensing*, 115:119–133, may 2016. ISSN 09242716. doi: 10.1016/j.isprsjprs.2015.10.012. URL http://www.sciencedirect.com/science/article/pii/S0924271615002439.

Michel Loreau. Linking biodiversity and ecosystems: towards a unifying ecological theory. *Philosophical Transactions of the Royal Society of London B: Biological Sciences*, 365(1537):49–60, 2010.

E Mayr and P D Ashlock. *Principles of Systematic Zoology*. McGraw-Hill, 1991. ISBN 9780071127011.

Ernst Mayr. Speciation Phenomena in Birds. *American Naturalist*, 74(752), 1940. doi: 10.1086/280892.

Patrick Mikalef, Ilias O. Pappas, John Krogstie, and Michail Giannakos. Big data analytics capabilities: a systematic literature review and research agenda. *Information Systems and e-Business Management*, 2018. ISSN 16179854. doi: 10.1007/s10257-017-0362-y.

National Aeronautics and Space Administration, National Oceanic Administration, and Atmospheric. Joint Polar Satellite System, 2020. URL https://www.jpss.noaa.gov/.

Laetitia M. Navarro, Néstor Fernández, Carlos Guerra, Rob Guralnick, W. Daniel Kissling, Maria Cecilia Londoño, Frank Muller-Karger, Eren Turak, Patricia Balvanera, Mark J. Costello, Aurelie Delavaud, G. Y. El Serafy, Simon Ferrier, Ilse Geijzendorffer, Gary N. Geller, Walter Jetz, Eun Shik Kim, Hye Jin Kim, Corinne S. Martin, Melodie A. McGeoch, Tuyeni H. Mwampamba, Jeanne L. Nel, Emily Nicholson, Nathalie Pettorelli, Michael E. Schaepman, Andrew Skidmore, Isabel Sousa Pinto, Sheila Vergara, Petteri Vihervaara, Haigen Xu, Tetsukazu Yahara, Mike Gill, and Henrique M. Pereira. Monitoring biodiversity change through effective global coordination. *Current Opinion in Environmental Sustainability*, 29:158–169, 2017. ISSN 18773435. doi: 10.1016/j.cosust.2018.02.005.

OpenStreetMap Contributors. OpenStreetMap (OSM), 2019. URL `https://www.openstreetmap.org`.

Claus Pahl and Brian Lee. Containers and clusters for edge cloud architectures-A technology review. In *Proceedings - 2015 International Conference on Future Internet of Things and Cloud*, pages 379–386, 2015. ISBN 9781467381031. doi: 10.1109/FiCloud.2015.35.

S Pavoine and M B Bonsall. Measuring biodiversity to explain community assembly: a unified approach. *Biol Rev Camb Philos Soc*, 86(4):792–812, 2011. ISSN 1469-185X (Electronic) 0006-3231. URL `https://onlinelibrary.wiley.com/doi/full/10.1111/j.1469-185X.2010.00171.x`.

H. M. Pereira, S. Ferrier, M. Walters, G. N. Geller, R. H.G. Jongman, R. J. Scholes, M. W. Bruford, N. Brummitt, S. H.M. Butchart, A. C. Cardoso, N. C. Coops, E. Dulloo, D. P. Faith, J. Freyhof, R. D. Gregory, C. Heip, R. Höft, G. Hurtt, W. Jetz, D. S. Karp, M. A. McGeoch, D. Obura, Y. Onoda, N. Pettorelli, B. Reyers, R. Sayre, J. P.W. Scharlemann, S. N. Stuart, E. Turak, M. Walpole, and M. Wegmann. Essential biodiversity variables, 2013. ISSN 10959203.

Henrique M. Pereira, Paul W. Leadley, Vânia Proença, Rob Alkemade, Jörn P.W. Scharlemann,

Juan F. Fernandez-Manjarrés, Miguel B. Araújo, Patricia Balvanera, Reinette Biggs, William W.L. Cheung, Louise Chini, H. David Cooper, Eric L. Gilman, Sylvie Guénette, George C. Hurtt, Henry P. Huntington, Georgina M. Mace, Thierry Oberdorff, Carmen Revenga, Patrícia Rodrigues, Robert J. Scholes, Ussif Rashid Sumaila, and Matt Walpole. Scenarios for global biodiversity in the 21st century. *Science*, 330(6010):1496–1501, 2010. ISSN 10959203. doi: 10.1126/science.1196624.

Susana Perez, Robert Jandl, and Agustín Rubio. Modelización del secuestro de carbono en sistemas forestales: Efecto de la elección de especie. *Ecología*, 21:341–352, 2007. ISSN 02140896.

Jeffrey M. Perkel. A toolkit for data transparency takes shape. *Nature*, 560(7719):513–515, aug 2018. ISSN 0028-0836. doi: 10.1038/d41586-018-05990-5. URL `http://www.nature.com/articles/d41586-018-05990-5`.

Paul Ramsey, Sandro Santilli, Regina Obe, Mark Cave-Ayland, and Bborie Park. PostGIS, 2018. URL `http://www.postgis.org/`.

David Reinsel, John Gantz, and John Rydning. The Digitization of the World - From Edge to Core. *IDC White Paper*, (US44413318), 2018. URL `https://www.seagate.com/www-content/our-story/trends/files/idc-seagate-dataage-whitepaper.pdf`.

Marko a. Rodriguez. The Gremlin Graph Traversal Machine and Language. *Proc. 15th Symposium on Database Programming Languages*, pages 1–10, 2015. doi: 10.1145/2815072.2815073. URL `http://arxiv.org/abs/1508.03843{%}5Cnhttp://dx.doi.org/10.1145/2815072.2815073`.

Håvard Rue and Leonhard Held. *Gaussian markov random fields: Theory and applications.* Chapman & Hall/CRC, 2005. ISBN 9780203492024. doi: 10.1198/tech.2006.s352. URL `https://www.crcpress.com/Gaussian-Markov-Random-Fields-Theory-and-Applications/Rue-Held/p/book/9781584884323`.

J. Rzedowski. *The vegetation of Mexico.* Comisión Nacional para el Conocimiento y Uso de la Biodiversidad, Mexico, 1ra. edici edition, 2006. ISBN 9681800028. URL https://www.cabdirect.org/cabdirect/abstract/19810673948.

John Salvatier, Thomas V. Wiecki, and Christopher Fonnesbeck. Probabilistic programming in Python using PyMC3. *PeerJ Computer Science*, 2:e55, apr 2016. ISSN 2376-5992. doi: 10.7717/peerj-cs.55. URL https://peerj.com/articles/cs-55.

José Sarukhán, Patricia Koleff, Julia Carabias, Jorge Soberón, Rodolfo Dirzo, Jorge Llorente-Bousquets, Gonzalo Halffter, Renée González, Ignacio March, Alejandro Mohar, Salvador Anta, and Javier de la Maza. Capital Natural de Mexico. Síntesis: Conocimiento actual y perspectivas de sustentabilidad. *Comisión Nacional para el Conocimiento y Uso de la Biodiversidad, México*, 2009. ISSN 1098-6596. doi: 10.1017/CBO9781107415324.004.

Simon Scheiter, Liam Langan, and Steven I. Higgins. Next-generation dynamic global vegetation models: Learning from community ecology. *New Phytologist*, 198(3):957–969, 2013. ISSN 0028646X. doi: 10.1111/nph.12210.

Dirk S. Schmeller, Jean Baptiste Mihoub, Anne Bowser, Christos Arvanitidis, Mark J. Costello, Miguel Fernandez, Gary N. Geller, Donald Hobern, W. Daniel Kissling, Eugenie Regan, Hannu Saarenmaa, Eren Turak, and Nick J.B. Isaac. An operational definition of essential biodiversity variables. *Biodiversity and Conservation*, 26(12):2967–2972, 2017. ISSN 15729710. doi: 10.1007/s10531-017-1386-9.

Skipper Seabold and Josef Perktold. Statsmodels: Econometric and Statistical Modeling with Python. *PROC. OF THE 9th PYTHON IN SCIENCE CONF*, 2010. URL http://conference.scipy.org/proceedings/scipy2010/pdfs/seabold.pdf.

Jerry Shannon and Kyle Walker. Opening GIScience: A process-based approach. *International Journal of Geographical Information Science*, 32(10):1911–1926, 2018. ISSN 13623087. doi: 10.

1080/13658816.2018.1464167. URL https://doi.org/10.1080/13658816.2018.1464167.

Nigel (technige) Small. py2neo [Computer Software], 2017. URL https://py2neo.org/v3/index.html.

Thomas F Stocker, Dahe Qin, Gian-Kasper Plattner, M Tignor, Simon K Allen, Judith Boschung, Alexander Nauels, Yu Xia, Vincent Bex, and Pauline M Midgley. *(IPCC) Climate Change 2013: The Physical Science Basis*. 2013.

Brian L. Sullivan, Christopher L. Wood, Marshall J. Iliff, Rick E. Bonney, Daniel Fink, and Steve Kelling. eBird: A citizen-based bird observation network in the biological sciences. *Biological Conservation*, 2009. ISSN 00063207. doi: 10.1016/j.biocon.2009.05.006.

R Development Core Team and R R Development Core Team. R: A Language and Environment for Statistical Computing. *R Foundation for Statistical Computing*, 1(2.11.1):409, 2016. ISSN 3-900051-07-0. doi: 10.1007/978-3-540-74686-7. URL http://www.r-project.org.

Lenny Teytelman. No more excuses for non-reproducible methods. *Nature*, 560(7719):411, 2018. ISSN 14764687. doi: 10.1038/d41586-018-06008-w.

Daniel Thornton, Kathy Zeller, Carlo Rondinini, Luigi Boitani, Kevin Crooks, Christopher Burdett, Alan Rabinowitz, and Howard Quigley. Assessing the umbrella value of a range-wide conservation network for jaguars (Panthera onca). *Ecological Applications*, 26(4):1112–1124, jun 2016. ISSN 10510761. doi: 10.1890/15-0602. URL http://doi.wiley.com/10.1890/15-0602.

UNEP/CBD. Cancun declaration of like-minded megadiversity countries. In *United Nations Environmental Program-Convention on Biological Diversity (UNEP-CBD)*, page UNEP/CBD/COP/6/INF/33, The Hague, Netherlands, 2002.

UNEP/CBD. Like-minded mega-diverse countries carta to achieve Aichi biodiversity Target 11. In *United Nations Environmental Program-Convention on Biological Diversity (UNEP-CBD)*,

page UNEP/CBD/COP/13/INF/45, Cancún, México, 2016. URL https://www.cbd.int/doc/meetings/cop/cop-13/information/cop-13-inf-45-en.pdf.

Martijn P. van Iersel, Alexander R. Pico, Thomas Kelder, Jianjiong Gao, Isaac Ho, Kristina Hanspers, Bruce R. Conklin, and Chris T. Evelo. The BridgeDb framework: Standardized access to gene, protein and metabolite identifier mapping services. *BMC Bioinformatics*, 11, 2010. ISSN 14712105. doi: 10.1186/1471-2105-11-5.

Chad Vicknair, Michael Macias, Zhendong Zhao, Xiaofei Nan, Yixin Chen, and Dawn Wilkins. A comparison of a graph database and a relational database. In *Proceedings of the 48th Annual Southeast Regional Conference on - ACM SE '10*, page 1, New York, New York, USA, 2010. ACM Press. ISBN 9781450300643. doi: 10.1145/1900008.1900067. URL http://portal.acm.org/citation.cfm?doid=1900008.1900067.

Rosalia. Vidal Zepeda. *Las regiones climaticas de Mexico 1.2.2.* UNAM, Instituto de Geografia, 2005. ISBN 9789703223947. URL https://books.google.co.uk/books?hl=es{&}lr={&}id=6xvqM4XQRFUC{&}oi=fnd{&}pg=PA15{&}dq=mexico+regiones+climatica{&}ots=D1R3erwtVq{&}sig=A91KR1-SGT8AbAFqFpFqOtqZ0-k{&}redir{_}esc=y{#}v=onepage{&}q=mexicoregionesclimatica{&}f=false.

Jin Feng Wang, Tong Lin Zhang, and Bo Jie Fu. A measure of spatial stratified heterogeneity. *Ecological Indicators*, 67:250–256, 2016. ISSN 1470160X. doi: 10.1016/j.ecolind.2016.02.052. URL http://dx.doi.org/10.1016/j.ecolind.2016.02.052.

Alexandra Weigelt, Elisabeth Marquard, Vicky M. Temperton, Christiane Roscher, Christoph Scherber, Peter N. Mwangi, Stefanievon Felten, Nina Buchmann, Bernhard Schmid, Ernst-Detlef Schulze, and Wolfgang W. Weisser. The Jena Experiment: six years of data from a grassland biodiversity experiment. *Ecology*, 2010. ISSN 0012-9658. doi: 10.1890/09-0863.1.

R. H. Whittaker. Evolution and Measurement of Species Diversity. *Taxon*, 21(2/3):213, 1972. ISSN 00400262. doi: 10.2307/1218190. URL https://www.jstor.org/stable/1218190?origin=crossref.

Stefan Wiemann and Lars Bernard. Spatial data fusion in Spatial Data Infrastructures using Linked Data. *International Journal of Geographical Information Science*, 30(4):613–636, apr 2016. ISSN 13623087. doi: 10.1080/13658816.2015.1084420. URL http://www.tandfonline.com/doi/full/10.1080/13658816.2015.1084420.

G Wilson, D A Aruliah, C T Brown, N P C Hong, M Davis, R T Guy, S H D Haddock, K D Huff, I M Mitchell, M D Plumbley, B Waugh, E P White, and P Wilson. Best Practices for Scientific Computing. *Plos Biology*, 12(1), 2014a. ISSN 1545-7885.

G. Wilson, D. A. Aruliah, C. Titus Brown, Neil P. Chue Hong, Matt Davis, Richard T. Guy, Steven H. D. Haddock, Kathryn D. Huff, Ian M. Mitchell, Mark D. Plumbley, Ben Waugh, Ethan P. White, and Paul Wilson. Best practices for scientific computing. *PLoS Biology*, 12(1):e1001745, jan 2014b. ISSN 1545-7885. doi: 10.1371/journal.pbio.1001745. URL http://dx.plos.org/10.1371/journal.pbio.1001745http://www.pubmedcentral.nih.gov/articlerender.fcgi?artid=3886731{&}tool=pmcentrez{&}rendertype=abstract{%}5Cnhttp://www.pubmedcentral.nih.gov/articlerender.fcgi?artid=3886731{%}7B{&}{%}7Dtool=pmcentrez{%}7B{&}{%}7Drendertype.

Supplementary material I

14. [Tutorial] Add data in Biospytial

Biospytial is a Knowledge Engine that merges different data using graph theory in order to model ecological big datasets using geostatistical, graph and other frameworks. Biospytial has reached a snapshop stage for initial release and will undergo further development.

14.1. Aims of this tutorial

This tutorial provides a simple guide on how to install new data sources. As an example, two data sources are installed: a vector-based data source called: `global_ecoregions` and raster based data source: `World Population for Latin America`.

14.2. Assumptions

A fully installed and running Biospytial Suite. This mean the three modules are running.

- Geoprocessing-Backend (GBP)
- Graph-Computing-Engine (GCE)
- Biospytial-Client. (BPE)

In addition, the datasources are downloaded and allocated in an accessible path from the Biospytial Client.

14.3. Converting the data to a Django Model

For data handling, Biospytial uses the ORM model for accessing geospatial data stored in the Geoprocessing-Backend. To achieve this, a Class called Model is specified using a given data-source. That is, each datasource has a class specification for communicating with the Relational Database manager.

14.4. Vector data

We make use of the tool `ogrinspect` to generate the model definition for a shapefile file and follow these steps.

1. Login to Biospytial-Client session (the bash shell and not the iPython environment).
2. Locate the path where the data are stored. In this case we are interested in adding the data-source ’terr-ecoregions-TNC’ which has an ESRI-Shapefile format.

14.4.1. Ingest the shapefile into the GPB

We make use of the `LayerMapping` utility. Use the tool `ogrinspect` described in the manage.py module inside the folder apps where all the Biospytial sources are located. The general syntax of this command is:

```
python manage.py ogrinspect [options] [options]
```

For this example:

```
python manage.py ogrinspect path_to/tnc_terr_ecoregions.shp TerrEcoregions \
                            --srid=4326 --mapping --multi
```

where the:

- `-srid` option sets the SRID for the geographic field.
- `-mapping` option tells ogrinspect to also generate a mapping dictionary for use with LayerMapping.
- `-multi` option is specified so that the geographic field is a MultiPolygonField instead of just a PolygonField.

More information is provided in: (`https://docs.djangoproject.com/en/2.0/ref/contrib/gis/tutorial/`)

The command prints in the standard output format the class definition for this dataset. If we decided to use the -mapping option a dictionary is also included with a standarized format for the column names.

14.5. *Export Shapefile into the Database (Geoprocessing Container)*

We use the LayerMapping utility to make this process faster. The first action is to edit or create the file load_shapefiles.py inside the ecoregions app.

We define here the mapping names dictionary (see above) and the necessary code to insert the shapefile into the database.

This is the content of the file load_shapefile.py

```
!/usr/bin/env python
-- coding: utf-8 --

from future import absolute_import, division, print_function, unicode_literals
import os from django.contrib.gis.utils
import LayerMapping from .models
import TerrEcoregions from biospytial
import settings

""" Functions for exporting shapefiles into the Postgis Database. """

author = "Juan Escamilla Molgora"
copyright = "Copyright 2018, JEM"
license = "GPL"
mantainer = "Juan"
email ="molgor@gmail.com"
```

```
#Generated by ogrinspect

terrecoregions_mapping = { 'eco_id_u' : 'ECO_ID_U',
                           'eco_code' : 'ECO_CODE',
                           'eco_name' : 'ECO_NAME',
                           'eco_num' : 'ECO_NUM',
                           'ecode_name' : 'ECODE_NAME',
                           'cls_code' : 'CLS_CODE',
                           'eco_notes' : 'ECO_NOTES',
                           'wwf_realm' : 'WWF_REALM',
                           'wwf_realm2' : 'WWF_REALM2',
                           'wwf_mhtnum' : 'WWF_MHTNUM',
                           'wwf_mhtnam' : 'WWF_MHTNAM',
                           'realmmht' : 'RealmMHT',
                           'er_update' : 'ER_UPDATE',
                           'er_date_u' : 'ER_DATE_U',
                           'er_ration' : 'ER_RATION',
                           'sourcedata' : 'SOURCEDATA',
                           'geom' : 'MULTIPOLYGON', }

file_shp = os.path.abspath( os.path.join(settings.PATH_RAWDATASOURCES,
                                         'terr-ecoregions-TNC',
                                         'tnc_terr_ecoregions.shp'), )
```

```
def run(verbose=True):
    lm = LayerMapping( TerrEcoregions, file_shp,
                       terrecoregions_mapping, transform=False, )
    lm.save(strict=True, verbose=verbose)
```

To load the layer, one must log into the Biospytial iPython environment with:

```
python manage.py shell
```

Inside the BCE module (e.g. ssh) and using the iPython console, run the following:

```
from ecoregions import load_shapefiles
load_shapefiles.run()
```

14.6. Example 2: Adding vector data

Download the roads shapefile from: `http://www.conabio.gob.mx/informacion/gis/maps/geo/carre1mgw.zip`

Using the `ogronspect` tool we have the following:

```
This is an auto-generated Django model module created by ogrinspect.


from django.contrib.gis.db import models


class MexRoads(models.Model):
    fnode_field = models.BigIntegerField()
    tnode_field = models.BigIntegerField()
    lpoly_field = models.BigIntegerField()
    rpoly_field = models.BigIntegerField()
    length = models.FloatField()
    cov_field = models.BigIntegerField()
    cov_id = models.BigIntegerField()
```

```
    geom = models.MultiLineStringField(srid=4326)

#Auto-generated LayerMapping dictionary for MexRoads model

mexroads_mapping = { 'fnode_field' : 'FNODE_',
 'tnode_field' : 'TNODE_',
 'lpoly_field' : 'LPOLY_',
 'rpoly_field' : 'RPOLY_',
 'length' : 'LENGTH',
 'cov_field' : 'COV_',
 'cov_id' : 'COV_ID',
 'geom' : 'MULTILINESTRING'
 }
```

14.7. Add raster data

As before, this process involves two steps: *i)* loading the datasource into the database and *ii)* creating a Class definition for the datasource, intepreted by the engine.

14.7.1. Add the data to the database

We use the raster support from Postgis. We use the script: `migrateToPostgis.bash` located in: `/apps/raster_api/bash_raster_tools/bash_scripts`

However, the tools for ingesting data into the database are stored in the Geospatial Processing Container. We need to log into this container and run the above file. You can copy the `bash_raster_tools` inside this container and run the command `migrateToPostgis.bash`.

Example. Running the following line will load the dataset into the database.

```
migrateToPostgis.bash [RasterData.tif]
```

14.7.2. *Create a class definition for Raster Data*

We need to add the Model Class definition inside the file: `raster_api/models.py`

The base class is GenericRaster. We need to extend this class into a new definition according to the type of data we are loading.

The following code describes a generic template for creating a class definition.

```
class myNewModel(GenericRaster):
    """
    ..
    Description of the model in plain words.
    Attributes
    ==========
    Default attributes given by the raster2pgsql
    id : int Unique primary key
        This is the id number of each element in the mesh.

    """
    number_bands = 1
    neo_label_name = 'name of node class'(optional)
    link_type_name = 'name of associated edges'(optional)
    units = 'The measurment units name'

    class Meta:
        managed = False
        db_table = 'name of table in DB'
```

```
    def __str__(self):
        c = "< String representation: %s >"
        return c
```

The last step is to add this new model into the `raster_models_dic` in the `settings.py` file.

```
raster_models_dic = {
’WindSpeed’ : raster_models[7],
’Elevation’ : raster_models[0],
’Vapor’ : raster_models[6],
’MaxTemperature’ : raster_models[5] ,
’MinTemperature’ : raster_models[4] ,
’MeanTemperature’ : raster_models[3] ,
’SolarRadiation’ : raster_models[2],
’Precipitation’ : raster_models[1],
’WorldPopLatam2010’ : raster_models[8] ,
’myNewModel’ : raster_models[9],
}
```

Suplementary materials II

This section gives a brief description of the mathematical and biological terms used in the paper. It also includes formalization of the data specification and some conceptual and theoretical consequences.

15. Mathematical definitions

Definition 1 (Equivalent class). *Let Ω be a set. An equivalent relation on Ω is a subset $R \subseteq \Omega \times \Omega$ that satisfies the following three properties:*

- Reflexivity*: For all $x \in \Omega, (x, x) \in R$*
- Symmetry*: For all $x \in \Omega$ and $y \in \Omega$, if $(x, y) \in R$ then $(y, x) \in R$*
- Transitivity*: For all $x, y, z \in \Omega$ if $(x, y) \in R$ and $(y, z) \in R$ then $(x, z) \in R$*

The equivalent class of an element $x \in \Omega$ is denoted as the set:

$$[x]_R = \{x \in \Omega | (x, y) \in R, y \in \Omega\} \tag{1}$$

Given that x and y are elements of Ω it follows that if $(x, y) \in R$ then $[x]_R \subseteq \Omega$.

Definition 2 (Partition). *Let Ω be a set and $\mathscr{A} = \{A_1, A_2, ..., A_n\}$. $\mathscr{A}$ is called a partition of Ω if and only if:*

- $\cup_{i=1}^{n} A_i = \Omega$
- $A_i \neq \emptyset$
- $A_i \cup A_j = \emptyset$ *for all* $i \neq j$

Definition 3 (Modulus). *Let $\mathscr{F} = \{[x]_R | x \in \Omega\}$ that is, the family of all equivalent classes in Ω defined by the relationship R. This set ($\mathscr{F}$) is denoted as $\Omega \backslash R$ and is called the quotient set of Ω by R or* Ω modulo R.

$\Omega \backslash R$ is a partition of Ω if and only if R is an equivalence relation. Therefore, any pair of elements A_i, A_j in $\Omega \backslash R$ (subsets of Ω) are mutually exclusive. A feature that, with the right caveats, eases the computation of probabilities using the rule of total probability. For example conditional autoregressive models use spatial lattices that partitions space in mutually exclusive areas, the aggregated measurements on each area simplifies the computing of spatial correlations in large areas (Besag, 1974).

Definition 4 (Graph or Network). *Let $V(G)$ be a set and $E(G) \subseteq V(G) \times V(G)$. A graph G is a duple given by $(V(G), E(G))$. $V(G)$ is the set of vertices of the graph and $E(G)$ is the set of edges. An example of a graph is drawn in figure: 2.1.*

Definition 5 (Subgraph). *Let G be a graph. G' is a subgraph of G ($G' \subseteq G$) if and only if $V(G') \subseteq V(G)$ and $E(G') \subseteq E(G)$.*

Definition 6 (Connected and acyclic graph). *If for every $u, v \in V(G)$ there exists a path that connects them, then G is said to be connected. If that path is unique for every u, v then G is acyclic (without cycles).*

Definition 7 (Tree). *A graph T which is connected and non-cyclic is called a* Tree. *An example is given in figure 2.2.*

Definition 8 (Subtree). *Let T be a tree. A subtree T' is a subgraph of T such that is also a tree (i.e. contains no cycles).*

15.1. Biological definitions

Definition 9 (Biological Species). *The following definitions are equivalent:*

- *Groups of actually or potentially interbreeding natural populations which are reproductively isolated from other such groups ((Mayr, 1940)).*
- *An inclusive Mendelian population; it is integrated by the bonds of sexual reproduction and parentage ((Dobzhansky and Dobzhansky, 1970): 354).*
- *A species is a group of interbreeding natural populations that is reproductively isolated from other such groups ((Mayr and Ashlock, 1991))*

Definition 10 (Taxonomic concept of species). '... a species consists of all the specimens which are, or would be, considered by a particular taxonomist to be members of a single kind as shown by the evidence or the assumption that they are as alike as their offspring or their hereditary relatives within a few generations. When there is no evidence of the hereditary relationship, the taxonomist will rely on distinctions that have been found to be effective in segregating species among other groups'. ((Blackwelder, 1967) : 164)

The concept of species is mostly biased by the data used. In the practical case is based in natural museum records around the world (See section on Data used and GBIF page: 19). Therefore, a more restrictive definition should be used in order to support further argumentations on evolution and ecology.

16. Theoretical consequences

Lemma 1. *There is a unique Taxonomic Tree of all life on Earth. This tree is called The Tree of Life.*

Proof 1. *All organisms have Common Ancestor. Because of this is possible to build taxonomic relationships based on this comparison. The Uniqueness of this common ancestor and the existence of LUA implies that: i) there is just one path that connects any pair of species (vertices) and ii) the graph is connected.*

Lemma 2 (Local Tree). *For any area in Earth it is possible to derive a unique Taxonomic Tree.*

Proof 2. *Because Life is Conspicuous it is possible to find organisms in any place. By the axioms of Common Ancestor and Taxonomic Relationship it is possible to build a taxonomic hierarchy between the group of organisms within that place. Because Axiom of LUA there is only one tree that represents these taxonomic /ancestry relationships.*

Proposition 1. *For a given area*[9] *in Earth, the taxonomic tree derived from it is a subtree of the Tree of Life.*

Proof 3. *Let T be the Tree of Life and $T(A)$ the local tree in the area A. $A \subseteq Earth$. $T(A)$ is a tree because of lemma 1.14. $T(A)$ is based on the same taxonomy given by the species in A (which are leaves in the tree) therefore all the edges of $T(A)$ are in T. The species in A is a subset of all the species in the $Earth$ otherwise the $Earth$ would not be the $Earth$ and there exist another greater set that could be called $Earth$.*

Corollary 1. *If $A = Earth$ then $T(A)$ = Tree of Life.*

Proof 4. *Let $A = Earth$. This implies that all species in A are in $Earth$ and vice versa. $V(T(Earth)) = V(TreeofLife)$ and the taxonomic chain (path) of $V(T(Earth))$ is the same as in $V(TreeofLife)$ because it is unique. Therefore, $TreeofLife = T(Earth)$*

17. Formal data specification

This section explains the mathematical formalities of the model. For the purposes of this treatment we will call Ω the total sample. In the current implementation the GBIF dataset is the only source of information for occurrences, therefore Ω = GBIF for an arbitrary chosen snapshot (version). In general, $\Omega \subset \mathscr{B}$ where $\mathscr{B}$ is the totality of living beings in Earth (the biosphere) for a given time t [10].

Raw Occurrence Data Let $o \in \Omega$ be called an Occurrence. o has attached a set of properties $\mathscr{P}(o)$.

In the case of the GBIF database, $\mathscr{P}(o)$ consists (but not exclusively) of:

- Species

[9] Any open set contained in the surface Earth. Earth can be considered as a compact surface embedded in $\mathbb{R}^3$

[10] If it would be necessary to clarify further we will write this as Ω_t

- Genus
- Family
- Order
- Class
- Phylum (or Division)
- Kingdom
- Location (lat/long) (point)
- time-stamp of collection
- Unique Id

The first eight properties are called **taxonomic properties**.

17.0.1. Towards integrated modelling

The concept of *equivalence class* is foundational because the set of properties $\mathscr{P}$ give a direct classification for living beings. In any ecological study, the sample (e.g. GBIF) will always be a subset of the universal set of *Life in Earth*. Each element in the sample has certain properties like acquisition time, location and, of course, the ontological properties of each particular study (e.g. individuals within a population; plant traits within an ecosystem; pollinators and plants, vectors and diseases, etc.)

A general modelling of properties derived by *equivalence relations* can model different representations of the same phenomenon in a generic way. For example, all occurrences have the attribute *Species Name*. If the relation (x, y) is: *x is the same species as y*; we have that the relation is indeed an **equivalence relation**. Continuing through this line of thought we have that the following relations are **equivalent relations** and each one defines as well a quotient set.

Relation	Quotient Set (notation)
x:has_the_same_id_as:y	$[Id]$
x:is_the_same_species_as:y	$[Sp]$
x:is_the_same_genus_as:y	$[Gns]$
x:is_the_same_family_as:y	$[Fam]$
x:is_the_same_order_as:y	$[Ord]$
x:is_the_same_class_as:y	$[Cls]$
x:is_the_same_phylum_as:y	$[Phy]$
x:is_the_same_kingdom_as:y	$[Kng]$
x:is_a_living_being_as:y	$[Root]$

By recursion, if Ω is a partition of a larger set say, Γ, any partition (equivalence relation) within Ω is also a partition of Γ. The models for Ω will be valid for Γ also.

For example: suppose that every occurrence is an organism. Every organism is constituted by cells. If Γ is the set of all cells then clearly Ω will be a partition under the equivalence relation: *x is a cell of the same organism as y*.

The above formalization of *taxonomic objects* can continue indefinitely. An unbounded object like this will always be in a state of definition but not fully defined. A theory or methodological framework needs to be able to add-up new possible properties in which the objects could be partitioned.

17.0.2. Adding more properties

Suppose that a new property P is added to each element of Ω. The new property P could be any type, e.g. binary, categorical or continuous, and determines a new equivalence relation such that a new quotient set $\Omega \backslash P$ can be derived. Any new property that splits Ω in a partition is an equivalence relation.

17.0.3. Partial orders and semi-lattice systems

The hierarchical ordering of: *kingdom, phylum, class, order, family, genus* and *species* is based on the *natural system.* If this order acts on the entire set of species on Earth (the biosphere $\mathscr{B}$), with the inclusion of LUA (Axiom 1.5) it defines a partial order set [11].

A consequence of being a **partial order set** is that, for every species s there exists a unique chain of ordered elements that join s with a genus gn, a family f, ..., a kingdom k.e.g., The species *Homo sapiens (L. 1758)* has an ordered chain of: *H. sapiens* $\leqslant$ Homo $\leqslant$ Hominidae $\leqslant$ Primates $\leqslant$ Mammalia $\leqslant$ Chordata $\leqslant$ Animalia. A partial order set induces a semi-lattice data structure compatible with ontology specifications and the spatial lattices framework. Using both types of relations is a first approach to define graph traversals based on spatial and evolutionary relationships. This can help to analyse species distributions, co-occurrence relationships and statistical modelling of ecological properties.

[11] Ergo, the $biosphere$ is a partial ordered set. For formal definition see: L.A. Skornyakov (originator) (2014)

Responses to reviewers

Click here to access/download;Personal Cover;Molgora_coverLetter+ReplytoReviewers.pdf

Lancaster Environment Centre | Lancaster University | Lancaster University Medical School

Lancaster, 28/02/2020

Dear Dr Nicole Nogoy,

Editor of GigaScience

and reviewers

Object: **Manuscript GIGA-D-19-00265 "Biospytial: spatial graph-based computing engine for ecological big data".**

We are grateful to the reviewers for their comments which helped in improving the quality of the manuscript. We also thank the Editor for giving us the opportunity to revise the manuscript. We apologize for the delayed response and we thank you and the reviewers for your patience during this long process also.

We took on board all the comments as shown in the point to point reply to reviewers starting in the next page. The software is publicly available via github, and the data will be upload in SciCrunch shortly.

We hope these corrections satisfactorily address the various comments made by the Reviewers. Please let us know if any further corrections are required.

Yours sincerely,

Juan Escamilla Molgora on behalf of all the authors.

Point to point reply to reviewers

Reviewer 1

I very much enjoyed reading this paper and as a geospatial data scientist/GIScientist am very happy to see this considered for GigaScience. I found the paper to be extremely comprehensive, very well-written, sound it computational approach, and reflecting a good knowledge of ecoinformatics that supports the global ecological community. The system architecture described is quite exciting. I admit to a lower level of expertise where knowledge engines are concerned, but know of many colleagues in the geospatial community who will be excited to see this new engine for **ecological data** that incorporates semantic relations and integrates into the geospatial semantic web. Unfortunately these colleagues do not read GigaScience, but I will guide them appropriately. :-)

> We thank the reviewer for the very positive general comment on our paper. We do hope to attract new readers to GigaScience.

Elaborate more discussions around the SDI

I found it interesting that the authors chose “spatial data infrastructure” as their first key word. I think I know where they are heading with this, and this is an important connection to make, but might they consider adding a big more to the introduction or concluding sections of the paper to make a stronger connection to the traditional “SDI” community? For instance, I am wondering how their engine qualifies as an SDI in the more traditional sense of the term. A traditional SDI implements a broader framework of geographic data, metadata, standards, institutional arrangements, policies, and tools that are interactively connected in order to make the use of spatial data more efficient and flexible. European INSPIRE is a shining example of this, as well as the United Nation’s SDI, and the Convention on Biological Diversity. Could the authors briefly add how Biospytial is aiding in SDI beyond just employing GBIF or IUCN Red List data as a use case?

> We fully agree with this vision of SDI and we thank the reviewer for raising this valid point. We have contextualised our engine in the light of current SDI definition. See lines 151-156.

By the way, how in the world does one properly pronounce “biospytial” (long y or short y). Could the authors provide a hint, if nothing else, just in their response to me as a reviewer. :-) In my mind I kept wanting to say “biospatial” with an “a!”

> This comment is very useful, we have explained why we called the engine biospytial and how to pronounce it. See lines: 148 and 149.

Another very minor observation: the authors identify their GPU (Geospatial Processing Unit). So many of us are used to understanding GPU in the already established Graphics Processing Unit parlance of computer hardware terminology.

> We have changed to RGU (relational geoprocessing unit) instead of using GPU.

More importantly, a major strength of this research is the interweaving of so many open source/open science technologies. I love Table 1 (a veritable “who’s who”). And I applaud the use of containerization. I couldn’t agree more with the statement on Line 172 that the idea here is to move the processes around, NOT the big data. I would add that the idea behind geospatial cloud computing, writ large, is to move the spatial analyses TO the data, rather than downloading or moving big data sets around. I mention this for discussion sake, not necessarily as a required change of wording in the paper.

> We fully agree with this comment, and we extend our sentence in line 191-193 in order to stress the importance of performing the analyses where the data is located. See new line aaa.

What I *do* further suggest for minor changes have to do with the authors’ important mention of reproducibility and replicability. Reproducibility is first mentioned on Line 17, and if I am understanding the authors’ intent, I would like to suggest a few more references that they might consider consulting and adding:

- Barba LA. 2018. Praxis of reproducible computational science. Authorea: doi: 10.22541/au.153922477.77361922. doi:10.22541/au.153922477.77361922.
- Jasny BR, Wigginton N, McNutt M, Bubela T, Buck S, et al. 2017. Fostering reproducibility in industry-academia research. Science 357(6353): 759.
- Teytelman L. 2018. No more excuses for non-reproducible methods. Nature 560: 411. doi: 10.1038/d41586-018-06008-w.
- Shannon J, Walker K. 2018. Opening GIScience: A process-based approach. International Journal of Geographical Information Science 32(10): 1911-1926, doi: 10.1080/13658816.2018.1464167.

On Lines 170 and 224 they talk about *replicating* their applications and/or analysis. Do they mean reproduce instead? In the literature there is now an important distinction between reproducibility (a condition where results or products can be continually reproduced using the same data and methods) and replicability (a higher level of scientific rigor where results or products can be reproduced using different samples of data and different software). In addition to the references above a good primer is now at https://sgsup.asu.edu/sites/default/files/rr_workshop_sparc_summary.pdf, as part of a recent workshop at https://sgsup.asu.edu/sparc/RRWorkshop. This part is a digression, just for discussion only: I think we can all acknowledge that a “reproducibility crisis” has received widespread attention across the sciences,

but perhaps nowhere as much as in psychology, where numerous attempts to reproduce previous findings have failed. It can be argued that scientists generally lack the relevant skills and tools to ensure that their findings are reproducible and replicable, and that much academic literature amounts to little more than advertising of findings, rather than detailed reporting that would allow results to be reproduced and replicated. “Show me” should be more important than “trust me” in the culture of science. Efforts to build a culture of open science, in which data, tools, methods, and software are all made accessible to everyone, are welcome. But openness in and of itself is not sufficient to ensure that results can be reproduced, let alone replicated.

> We fully agree with these comments and we thank the reviewer for sending us the references and workshop links. They were very useful and we have added a paragraph in the conclusions recognising the limitations in reproducibility and replicability in spatial analyses. We also added the Barba 2019, Teytelman and Shannon references. See lines 733 - 740.

Detected typos

Lines 85-86 - the full definition of the GEO BON acronym is Group on Earth Observations Biodiversity Observation Network (they are missing the BON part)

> Done.

Line 92 - I may have missed it, but can the authors please expand the PREDICTS acronym?

> Done.

Congratulations again to the authors for this fine work and best wishes to them for continued success.

> Thanks for your words we are really pleased with your comments and advices.

Reviewer 2

1.What classification algorithms are used to construct the tree?

> The taxonomic classification mentioned in various points in the manuscript, is based on the classical natural systematic classification of the species in the tree of life. Therefore this classification already exists and it is used here to organize the taxa in a hierarchical structure. To remove any confusion we added a reference of the updated taxonomic classification used here.

2.Spatial stratified heterogeneity (SSH) becomes a serious problem when data is big and diverse. A sample is biased to SSH population when the sample don't cover all strata; and statistics become confounded when they are applied globally to SSH population. Therefore, SSH should be tested at early stage of big spatial data analysis. IF SSH is insignificant, a global model is safe; otherwise, a simple solution is to apply a model in strata, separately.

> We thank the reviewer for this very good point. We have added a reference (Wang et al 2016) in line 87 recognising the important role of stratification to reduce bias in spatial analyses.

3.To illustrate the robust of the tool, the authors may provide several different examples for readers to practice. For example, cities evolution tree, besides the tree in the paper.

> We understand the importance of robustness of our proposed engine. We have shown that any spatial data with an existing structure can be employed in our engine. We believe that adding other examples is out of the scope. The algorithm is open and we hope that others will build up the portfolio of applications. However, we have added additional components to the jaguar example to fully show its aplicability.

4.To increase tool's users who are unfamiliar with computer language, draw a flowchart so a user can follow as he/she is doing in the real world.

> A full updated working example is provided towards the end of the paper.

Review 3

Overall This manuscript presents a knowledge engine designed to manage large spatial ecological data in a variety of formats and in an efficient way, using graph theory to maximise this efficiency, and enabling a series of operations. The manuscript is essentially composed of two sections, one in which the engine is described, and another one where its potential and applicability are shown in the form of meaningful examples.The paper is well written and structured, and presents a tool that can be of great use to ecologists and natural scientists, as well as to conservation managers with a natural sciences background. I suggest only some minor revisions, several of them asking for clarifications.

The jaguar example ends somewhat abruptly. In order to make a stronger point in showing the potential of the presented knowledge engine, it would be good if the authors would add a paragraph rounding up the results obtained in the exercise e.g. the reader ends up not being presented with the taxa most associated with the jaguar, or if these taxa were expected or else they are surprising. Linked to this, it would be interesting to follow up with the potential of the environmental layers to describe if the areas with jaguar are exceptional or not climatically, or topographically, or both, within Mexico. In other words, your example ends too quickly and more could be shown of it towards the end that would increase the perception of the reader regarding the potential and usefulness of the engine you present.

> We have added a new section in the manuscript (section 4.3) describing the taxa and environmental results for jaguars.

Linked to the above, you could be more creative with figure 6, and figure 7 is nice but too messy. Consider showing a subset, and discuss it more in the text.

> Both figures have been modified. Figure 8 (formerly 6) shows the elevation map (DEM) as base map displaying occurrences as points and environmental raster data objects as small overlapping regions. In figure 9 (formerly 7) we reduced the tree to only include orders, classes, phyla and kingdoms which reduces the number of nodes drastically. We improved the readability by assigning same size for all nodes' labels. We changed the color of the nodes representing the frequency (abundance) of taxa.

In page 24 and after, the codes include the term 'lambda' in many lines, and no explanation is given as to what that means. Could you specify what it means? Is it an anonymous function? In any case, this paper will be read by biologists with no background on computing science and the terms should be specified clearly.

> Full explanation of the lambda functions and the joint effect of the map-lambda expression was added. See lines

> 506 - 513 and lines: 519 - 522.

In page 32, the total area of the cells is computed. It is not clear what is the original area of each cell.

> We did the following amendments (lines: 599 - 608): * Added subsection for reprojecting to conic equal area for measuring areas in meters. * Added subsection for importing polygon from Mexico with reprojection. * Added total area calculation and average size for each cell.

The GBIF database is composed of points (coordinates). How is this translated to a cell of a given area? Are you using the 1' DEM or the 1km environmental layers? In any case, GBIF coordinates can be of varying reliability, and a buffer is normally advised. It would be good to know where the cell area comes from (it was not too clear in the manuscript as it stands), and whether the coordinates in GBIF are taken as precise points. See this as a reference: https://onlinelibrary.wiley.com/doi/full/10.1111/ele.12624

> We use the precise location (lat, lon wgs84 coordinates) of the GBIF occurrences given by their GBIF API / data (line 387). The occurrences are aggregated according to their taxonomy given that each occurrence belongs to a certain species. Although this was explained in lines: 403 - 407, we acknowledge that it was not clear enough. We added a more comprehensive explanation of the process for generating the local taxonomic trees on lines: 408 to 418.
>
> We thank the reviewer for pointing out the lack of clarity in the used grid. We included a brief description on how the grid system is created with a reference to the functions that generate customized grids (Lines: 397-399). In addition, we included a more explicit description of the grid used in 'worked example' under a new section named: 'Additional data used' (lines: 436-440).
>
> We agree on the importance of estimating multidimensional biases, gaps and uncertainties in opportunistic samplings and citizen science records such as GBIF. For this reason we decided to use the complete information of location (point coordinates) of every record. However, accounting for these problems in the current worked example is out of the scope of the engine at this moment. We are, however, optimistic that the engine will help identify better this limitations of the data with the use of automatic or semi automatic procedures applied to large volumes of occurrences. Nevertheless, we thank the reviewer for the

suggested reference as this is an issue that hopefully could be tackled in further applications of the engine.

The paragraph starting in line 511 contains a conclusion that is very difficult to sustain, since it is based on the assumption that threatened species are evenly distributed across the country. We know that this is not the case. I would be less categorical with it (i.e. it would seem that jaguars occur in places where other threatened species tend to cluster). The 'five times more likely' is not believable given the assumption.

Agreed, the paragraph is misleading or meaningless with the presented assumption. It has been removed.

In general, the use of numbers for references is fine, but in some instances, it is strange: e.g. in cases where you refer to a citation in the form "... [20] proposed that... ", it would help the readability to add "Smith et al. [20] proposed that... "

The numbering system for references have been changed to author names plus year.

Detected typos

All suggestions were covered. We thank the reviewer for her/his time and positive feedback.
