# Supplementary material for: Biospytial: spatial graph-based computing for ecological Big Data
Source: Gigascience. 2020 May 11;9(5):giaa039. doi: 10.1093/gigascience/giaa039 (PMC7213554; doi:10.1093/gigascience/giaa039)
Supplement: giaa039_Supplemental_Files [file giaa039_supplemental_files.zip › Adding_data_in_Biospytial.pdf]

Supplementary material I for *Biospytial: spatial graph-based computing for ecological big data*

1. Adding data in Biospytial

Biospytial is a Knowledge Engine that merges different data using graph theory in order to model ecological big datasets using geostatistical, graph and other frameworks. Biospytial has reached a snapshop stage for initial release and will undergo further development.

1.1. Aims of this tutorial

This tutorial provides a simple guide on how to install new data sources. As an example, two data sources are installed: a vector-based data source called: `global_ecoregions` and raster based data source: `World Population for Latin America`.

1.2. Assumptions

A fully installed and running Biospytial Suite. This mean the three modules are running.

- Geoprocessing-Backend (GBP)
- Graph-Computing-Engine (GCE)
- Biospytial-Client. (BPE)

In addition, the datasources are downloaded and allocated in an accessible path from the Biospytial Client.

1.3. Converting the data to a Django Model

For data handling, Biospytial uses the ORM model for accessing geospatial data stored in the Geoprocessing-Backend. To achieve this, a Class called Model is specified using a given datasource. That is, each datasource has a class specification for communicating with the Relational Database manager.

1.4. Vector data

We make use of the tool `ogrinspect` to generate the model definition for a shapefile file and follow these steps.

1. Login to Biospytial-Client session (the bash shell and not the iPython environment).
2. Locate the path where the data are stored. In this case we are interested in adding the datasource 'terr-ecoregions-TNC' which has an ESRI-Shapefile format.

1.4.1. Ingest the shapefile into the GPB

We make use of the `LayerMapping` utility. Use the tool `ogrinspect` described in the manage.py module inside the folder apps where all the Biospytial sources are located. The general syntax of this command is:

| python manage.py ogrinspect [options] [options]|
For this example:

```
python manage.py ogrinspect path_to/tnc_terr_ecoregions.shp TerrEcoregions \
                              --srid=4326 --mapping --multi
```

where the:

- `-srid` option sets the SRID for the geographic field.
- `-mapping` option tells ogrinspect to also generate a mapping dictionary for use with LayerMapping.
- `-multi` option is specified so that the geographic field is a MultiPolygonField instead of just a PolygonField.

More information is provided in: (`https://docs.djangoproject.com/en/2.0/ref/contrib/gis/tutorial/`)

The command prints in the standard output format the class definition for this dataset. If we decided to use the `-mapping` option a dictionary is also included with a standarized format for the column names.

1.5. Export Shapefile into the Database (Geoprocessing Container)

We use the `LayerMapping` utility to make this process faster. The first action is to edit or create the file `load_shapefiles.py` inside the `ecoregions` app.

We define here the mapping names dictionary (see above) and the necessary code to insert the shapefile into the database.

This is the content of the file `load_shapefile.py`

```
!/usr/bin/env python
-- coding: utf-8 --

from future import absolute_import, division, print_function, unicode_literals
import os from django.contrib.gis.utils
import LayerMapping from .models
import TerrEcoregions from biospytial
import settings

""" Functions for exporting shapefiles into the Postgis Database. """

author = "Juan Escamilla Molgora"
copyright = "Copyright 2018, JEM"
license = "GPL"
mantainer = "Juan"
email ="molgor@gmail.com"

#Generated by ogrinspect

terrecoregions_mapping = { 'eco_id_u' : 'ECO_ID_U',
                            'eco_code' : 'ECO_CODE',
                            'eco_name' : 'ECO_NAME',
                            'eco_num' : 'ECO_NUM',
                            'ecode_name' : 'ECODE_NAME',
                            'cls_code' : 'CLS_CODE',
                            'eco_notes' : 'ECO_NOTES',
                            'wwf_realm' : 'WWF_REALM',
                            'wwf_realm2' : 'WWF_REALM2',
                            'wwf_mhtnum' : 'WWF_MHTNUM',
                            'wwf_mhtnam' : 'WWF_MHTNAM',
```

```
                                'realmmht' : 'RealmMHT',
                                'er_update' : 'ER_UPDATE',
                                'er_date_u' : 'ER_DATE_U',
                                'er_ration' : 'ER_RATION',
                                'sourcedata' : 'SOURCEDATA',
                                'geom' : 'MULTIPOLYGON', }

file_shp = os.path.abspath( os.path.join(settings.PATH_RAWDATASOURCES,
                                         'terr-ecoregions-TNC',
                                         'tnc_terr_ecoregions.shp'), )

def run(verbose=True):
    lm = LayerMapping( TerrEcoregions, file_shp,
                      terrecoregions_mapping, transform=False, )
    lm.save(strict=True, verbose=verbose)
```

To load the layer, one must log into the Biospytial iPython environment with:

| python manage.py shell |
Inside the BCE module (e.g. ssh) and using the iPython console, run the following:

```
from ecoregions import load_shapefiles
load_shapefiles.run()
```

1.6. Example 2: Adding vector data

Download the roads shapefile from: `http://www.conabio.gob.mx/informacion/gis/maps/geo/carre1mgw.zip`
Using the `ogronspect` tool we have the following:

```
#This is an auto-generated Django model module created by ogrinspect.

from django.contrib.gis.db import models

class MexRoads(models.Model):
    fnode_field = models.BigIntegerField()
    tnode_field = models.BigIntegerField()
    lpoly_field = models.BigIntegerField()
    rpoly_field = models.BigIntegerField()
    length = models.FloatField()
    cov_field = models.BigIntegerField()
    cov_id = models.BigIntegerField()
    geom = models.MultiLineStringField(srid=4326)

#Auto-generated LayerMapping dictionary for MexRoads model

mexroads_mapping = { 'fnode_field' : 'FNODE_',
 'tnode_field' : 'TNODE_',
 'lpoly_field' : 'LPOLY_',
 'rpoly_field' : 'RPOLY_',
```

```
'length' : 'LENGTH',
'cov_field' : 'COV_',
'cov_id' : 'COV_ID',
'geom' : 'MULTILINESTRING'
}
```

1.7. Add raster data

As before, this process involves two steps: *i)* loading the datasource into the database and *ii)* creating a Class definition for the datasource, intepreted by the engine.

1.7.1. Add the data to the database

We use the raster support from Postgis. We use the script: `migrateToPostgis.bash` located in: `/apps/raster_api/bash_raster_tools/bash_scripts`

However, the tools for ingesting data into the database are stored in the Geospatial Processing Container. We need to log into this container and run the above file. You can copy the `bash_raster_tools` inside this container and run the command `migrateToPostgis.bash`.

Example. Running the following line will load the dataset into the database.

| migrateToPostgis.bash [RasterData.tif] |

1.7.2. Create a class definition for Raster Data

We need to add the Model Class definition inside the file: `raster_api/models.py`

The base class is GenericRaster. We need to extend this class into a new definition according to the type of data we are loading.

The following code describes a generic template for creating a class definition.

```
class myNewModel(GenericRaster):
    """
    ..
    Description of the model in plain words.
    Attributes
    ==========
    Default attributes given by the raster2pgsql
    id : int Unique primary key
        This is the id number of each element in the mesh.

    """
    number_bands = 1
    neo_label_name = 'name of node class'(optional)
    link_type_name = 'name of associated edges'(optional)
    units = 'The measurment units name'

    class Meta:
        managed = False
        db_table = 'name of table in DB'

    def __str__(self):
        c = "< String representation: %s >"
        return c
```

The last step is to add this new model into the `raster_models_dic` in the `settings.py` file.

```
raster_models_dic = {
'WindSpeed' : raster_models[7],
'Elevation' : raster_models[0],
'Vapor' : raster_models[6],
'MaxTemperature' : raster_models[5] ,
'MinTemperature' : raster_models[4] ,
'MeanTemperature' : raster_models[3] ,
'SolarRadiation' : raster_models[2],
'Precipitation' : raster_models[1],
'WorldPopLatam2010' : raster_models[8] ,
'myNewModel' : raster_models[9],
}
```
