# Supplementary material for: Biospytial: spatial graph-based computing for ecological Big Data
Source: Gigascience. 2020 May 11;9(5):giaa039. doi: 10.1093/gigascience/giaa039 (PMC7213554; doi:10.1093/gigascience/giaa039)
Supplement: giaa039_Supplemental_Files [file giaa039_supplemental_files.zip › Mathematical_formalisms.pdf]

Suplementary materials II for the paper: ***Biospytial: spatial graph-based computing for ecological big data***

1. Mathematical formalisms

This section gives a brief description of the mathematical and biological terms used in the paper. It also includes formalization of the data specification and some conceptual and theoretical consequences.

1.1. Mathematical definitions

Definition 1 (Equivalent class). *Let Ω be a set. An equivalent relation on Ω is a subset $R \subseteq \Omega \times \Omega$ that satisfies the following three properties:*

- Reflexivity*: For all $x \in \Omega, (x,x) \in R$*
- Symmetry*: For all $x \in \Omega$ and $y \in \Omega$, if $(x,y) \in R$ then $(y,x) \in R$*
- Transitivity*: For all $x,y,z \in \Omega$ if $(x,y) \in R$ and $(y,z) \in R$ then $(x,z) \in R$*

The equivalent class of an element $x \in \Omega$ is denoted as the set:

$$[x]_R = \{x \in \Omega | (x,y) \in R, y \in \Omega\} \tag{1}$$

Given that x and y are elements of Ω it follows that if $(x,y) \in R$ then $[x]_R \subseteq \Omega$.

Definition 2 (Partition). *Let Ω be a set and $\mathscr{A} = \{A_1, A_2, ..., A_n\}$. $\mathscr{A}$ is called a partition of Ω if and only if:*

- $\cup_{i=1}^{n} A_i = \Omega$
- $A_i \neq \emptyset$
- $A_i \cup A_j = \emptyset$ *for all $i \neq j$*

Definition 3 (Modulus). *Let $\mathscr{F} = \{[x]_R | x \in \Omega\}$ that is, the family of all equivalent classes in Ω defined by the relationship R. This set ($\mathscr{F}$) is denoted as $\Omega \backslash R$ and is called the quotient set of Ω by R or* Ω modulo R.

$\Omega \backslash R$ is a partition of Ω if and only if R is an equivalence relation. Therefore, any pair of elements A_i, A_j in $\Omega \backslash R$ (subsets of Ω) are mutually exclusive. A feature that, with the right caveats, eases the computation of probabilities using the rule of total probability. For example conditional autoregressive models use spatial lattices that partitions space in mutually exclusive areas, the aggregated measurements on each area simplifies the computing of spatial correlations in large areas [1].

Definition 4 (Graph or Network). *Let $V(G)$ be a set and $E(G) \subseteq V(G) \times V(G)$. A graph G is a duple given by $(V(G), E(G))$. $V(G)$ is the set of vertices of the graph and $E(G)$ is the set of edges. An example of a graph is drawn in figure: 2.1.*

Definition 5 (Subgraph). *Let G be a graph. G' is a subgraph of G ($G' \subseteq G$) if and only if $V(G') \subseteq V(G)$ and $E(G') \subseteq E(G)$.*

Definition 6 (Connected and acyclic graph). *If for every $u, v \in V(G)$ there exists a path that connects them, then G is said to be connected. If that path is unique for every u, v then G is acyclic (without cycles).*

Definition 7 (Tree). *A graph T which is connected and non-cyclic is called a* Tree. *An example is given in figure 2.2.*

Definition 8 (Subtree). *Let T be a tree. A subtree T' is a subgraph of T such that is also a tree (i.e. contains no cycles).*

1.2. Biological definitions

Definition 9 (Biological Species). *The following definitions are equivalent:*

- *Groups of actually or potentially interbreeding natural populations which are reproductively isolated from other such groups ([2]).*
- *An inclusive Mendelian population; it is integrated by the bonds of sexual reproduction and parentage ([3]: 354).*
- *A species is a group of interbreeding natural populations that is reproductively isolated from other such groups ([4])*

Definition 10 (Taxonomic concept of species). '... a species consists of all the specimens which are, or would be, considered by a particular taxonomist to be members of a single kind as shown by the evidence or the assumption that they are as alike as their offspring or their hereditary relatives within a few generations. When there is no evidence of the hereditary relationship, the taxonomist will rely on distinctions that have been found to be effective in segregating species among other groups'. ([5] : 164)

The concept of species is mostly biased by the data used. In the practical case is based in natural museum records around the world (See section on Data used and GBIF page: **??**). Therefore, a more restrictive definition should be used in order to support further argumentations on evolution and ecology.

2. Theoretical consequences

Lemma 1. *There is a unique Taxonomic Tree of all life on Earth. This tree is called The Tree of Life.*

Proof 1. *All organisms have Common Ancestor. Because of this is possible to build taxonomic relationships based on this comparison. The Uniqueness of this common ancestor and the existence of LUA implies that: i) there is just one path that connects any pair of species (vertices) and ii) the graph is connected.*

Lemma 2 (Local Tree). *For any area in Earth it is possible to derive a unique Taxonomic Tree.*

Proof 2. *Because Life is Conspicuous it is possible to find organisms in any place. By the axioms of Common Ancestor and Taxonomic Relationship it is possible to build a taxonomic hierarchy between the group of organisms within that place. Because Axiom of LUA there is only one tree that represents these taxonomic /ancestry relationships.*

Proposition 1. *For a given area*[1] *in Earth, the taxonomic tree derived from it is a subtree of the Tree of Life.*

Proof 3. *Let T be the Tree of Life and $T(A)$ the local tree in the area A. $A \subseteq Earth$. $T(A)$ is a tree because of lemma 1.14. $T(A)$ is based on the same taxonomy given by the species in A (which are leaves in the tree) therefore all the edges of $T(A)$ are in T. The species in A is a subset of all the species in the $Earth$ otherwise the $Earth$ would not be the $Earth$ and there exist another greater set that could be called $Earth$.*

Corollary 1. *If $A = Earth$ then $T(A) =$ Tree of Life.*

Proof 4. *Let $A = Earth$. This implies that all species in A are in $Earth$ and vice versa. $V(T(Earth)) = V(TreeofLife)$ and the taxonomic chain (path) of $V(T(Earth))$ is the same as in $V(TreeofLife)$ because it is unique. Therefore, $TreeofLife = T(Earth)$*

[1] Any open set contained in the surface Earth. Earth can be considered as a compact surface embedded in $\mathbb{R}^3$

3. Formal data specification

This section explains the mathematical formalities of the model. For the purposes of this treatment we will call Ω the total sample. In the current implementation the GBIF dataset is the only source of information for occurrences, therefore Ω = GBIF for an arbitrary chosen snapshot (version). In general, $\Omega \subset \mathscr{B}$ where $\mathscr{B}$ is the totality of living beings in Earth (the biosphere) for a given time t [2].

Raw Occurrence Data Let $o \in \Omega$ be called an Occurrence. o has attached a set of properties $\mathscr{P}(o)$. In the case of the GBIF database, $\mathscr{P}(o)$ consists (but not exclusively) of:

- Species
- Genus
- Family
- Order
- Class
- Phylum (or Division)
- Kingdom
- Location (lat/long) (point)
- time-stamp of collection
- Unique Id

The first eight properties are called **taxonomic properties**.

3.0.1. Towards integrated modelling

The concept of *equivalence class* is foundational because the set of properties $\mathscr{P}$ give a direct classification for living beings. In any ecological study, the sample (e.g. GBIF) will always be a subset of the universal set of *Life in Earth.* Each element in the sample has certain properties like acquisition time, location and, of course, the ontological properties of each particular study (e.g. individuals within a population; plant traits within an ecosystem; pollinators and plants, vectors and diseases, etc.)

A general modelling of properties derived by *equivalence relations* can model different representations of the same phenomenon in a generic way. For example, all occurrences have the attribute *Species Name.* If the relation (x, y) is: *x is the same species as y*; we have that the relation is indeed an **equivalence relation**. Continuing through this line of thought we have that the following relations are **equivalent relations** and each one defines as well a quotient set.

Relation	Quotient Set (notation)
x:`has_the_same_id_as`:y	$[Id]$
x:`is_the_same_species_as`:y	$[Sp]$
x:`is_the_same_genus_as`:y	$[Gns]$
x:`is_the_same_family_as`:y	$[Fam]$
x:`is_the_same_order_as`:y	$[Ord]$
x:`is_the_same_class_as`:y	$[Cls]$
x:`is_the_same_phylum_as`:y	$[Phy]$
x:`is_the_same_kingdom_as`:y	$[Kng]$
x:`is_a_living_being_as`:y	$[Root]$

[2] If it would be necessary to clarify further we will write this as Ω_t

By recursion, if Ω is a partition of a larger set say, Γ, any partition (equivalence relation) within Ω is also a partition of Γ. The models for Ω will be valid for Γ also.

For example: suppose that every occurrence is an organism. Every organism is constituted by cells. If Γ is the set of all cells then clearly Ω will be a partition under the equivalence relation: *x is a cell of the same organism as y.*

The above formalization of *taxonomic objects* can continue indefinitely. An unbounded object like this will always be in a state of definition but not fully defined. A theory or methodological framework needs to be able to add-up new possible properties in which the objects could be partitioned.

3.0.2. Adding more properties

Suppose that a new property P is added to each element of Ω. The new property P could be any type, e.g. binary, categorical or continuous, and determines a new equivalence relation such that a new quotient set $\Omega \backslash P$ can be derived. Any new property that splits Ω in a partition is an equivalence relation.

3.0.3. Partial orders and semi-lattice systems

The hierarchical ordering of: *kingdom, phylum, class, order, family, genus* and *species* is based on the *natural system.* If this order acts on the entire set of species on Earth (the biosphere $\mathscr{B}$), with the inclusion of LUA (Axiom 1.5) it defines a partial order set [3].

A consequence of being a **partial order set** is that, for every species s there exists a unique chain of ordered elements that join s with a genus gn, a family f, ..., a kingdom k.e.g., The species *Homo sapiens (L. 1758)* has an ordered chain of: *H. sapiens* ⩽ Homo ⩽ Hominidae ⩽ Primates ⩽ Mammalia ⩽ Chordata ⩽ Animalia. A partial order set induces a semi-lattice data structure compatible with ontology specifications and the spatial lattices framework. Using both types of relations is a first approach to define graph traversals based on spatial and evolutionary relationships. This can help to analyse species distributions, co-occurrence relationships and statistical modelling of ecological properties.

[3]Ergo, the $biosphere$ is a partial ordered set. For formal definition see: [6]

[1] Besag J. Spatial Interaction and the Statistical Analysis of Lattice Systems. Journal of the Royal Statistical Society Series B (Methodological). 1974;36(2):192–236. Available from: http://www.jstor.org/stable/2984812.

[2] Mayr E. Speciation Phenomena in Birds. American Naturalist. 1940;74(752).

[3] Dobzhansky T, Dobzhansky TG. Genetics of the Evolutionary Process. Columbia University Press; 1970.

[4] Mayr E, Ashlock PD. Principles of Systematic Zoology. McGraw-Hill; 1991.

[5] Blackwelder RE. Taxonomy: a text and reference book. Wiley; 1967.

[6] Skornyakov LAo. Partially ordered set. Encyclopedia of Mathematics. 2014;October. Available from: http://www.encyclopediaofmath.org/index.php?title=Partially{_}ordered{_}set{&}oldid=33633.
