# Supplementary material for: Biospytial: spatial graph-based computing for ecological Big Data
Source: Gigascience. 2020 May 11;9(5):giaa039. doi: 10.1093/gigascience/giaa039 (PMC7213554; doi:10.1093/gigascience/giaa039)
Supplement: giaa039_Supplemental_Files [file giaa039_supplemental_files.zip › [Official Demo]Co-ocurrences_Jaguar.html]

[Official Demo]Co-ocurrences\_Jaguar


# Working example¶

This section is a case study for analysing the frequency of coexistent taxonomic groups in all
the available dataset restricted to arbitrarily chosen branches of the Tree of Life and included in
a list of threatened species. These type of analyses are important in conservation studies, where
the characterisation of umbrella (or other surrogate) species constitute the basis for protecting a
significant number of associated species [5], [19]. To account for this effect, we chose the jaguar
(Panthera onca) as the species of interest. This due to its preference for undisturbed ecosystems
[82] and its wide geographic requiremental range; $181±4km^2$ for females and $431±152km^2$ males
[15]. We use the IUCN Red List of Threatened Species (Red List) [41] to account for the propor
tion of species (critically endangered, endangered or vulnerable) associated with the presence of
jaguars in a 4 t h degree neighbourhood using the 4km resolution grid described above. For do
ing so, we first calculate the local taxonomic tree for each cell-type node. The resulting trees are
aggregated into a single tree that contains the union of all the nodes found in the local trees. The
aggregated tree contains all the known co-occurrences of jaguar in a neighbourhood of degree 4 th .
We filter this tree to select only the nodes that match the Red List of threatened species and create
a new tree object using the selected nodes, an operation know as trimming. We rank all the node
of the resulting tree using the frequency of occurring at each neighboring cell, in order to provide
an estimate of which nodes co-occur more often with jaguars. Finally, we provide methods for
interactive visualisations of the spatial data and the network structure.
To show the capabilities of the engine we decided to process a reasonable number of cells and
trees. The time for executing the following example varies considerably depending on the group of
interest, the size of the neighbourhood and the computer platform. A quick workaround to speed
up the processes is to reduce the number of neighbouring cells (order of the neighbourhood), e.g.
use degree = 1.

## Obtaining and runing the engine¶

The engine is composed of three modules that can be downloaded and run as docker containers.

- Graph Storage and Processing Unit
- Biospytial Computing Engine
- Relational Geoprocessing Unit

The source code can be downloaded from here

**Note:**
To show the capabilities of the engine we decided to process a fair amount of Cells and Trees. The processing of this will take around 35 minutes in a 4 core computer.
It is possible to reduce the number of data to fasten the computation (for demonstration purposes). For doing this, change the order of the neighbourhood to 1. (See below)
For a fast computation reduce the order level to 1

In [1]:

```
## Import modules and libraries
%matplotlib inline
import sys
sys.path.append('/apps')
import django
django.setup()
import numpy as np
import matplotlib.pyplot as plt
## Jupyter only, use for displaying utf8 names. Deprecated in Biospytial 3. 
reload(sys)
sys.setdefaultencoding("utf-8")
```

```
INFO Merging Trees
INFO Sorting nodes in taxonomic levels by counts on frequencies
INFO Sorting nodes in taxonomic levels by counts on frequencies
INFO Sorting nodes in taxonomic levels by counts on frequencies
INFO Sorting nodes in taxonomic levels by counts on frequencies
```

## Selecting the *Jaguar* node¶

We begin by selecting the node in the Tree of Life corresponding to the genus Panthera. This
node is linked to some Species and Family type nodes and also has links to Occurrence nodes,
where the information of location and time is stored. To start the traversal we need to first select
this node. To do so we use the function pickNode using the following syntax:

```
pickNode(<Type of node>,'name of the node')
```

In the example below we see how to load the pickNode function and the appropriate node class
(in this case Genus ).

In [2]:

```
from drivers.graph_models import Genus, graph, pickNode
jaguars = pickNode(Genus,"Panthera")
```

The variable *jaguars* is now an instance of the class **Genus**. As such, it has associated attributes and methods.
string representation is the following:

In [3]:

```
jaguars
```

Out[3]:

```
<TreeNode type: Genus id = 2435194 name: Panthera>
```

We proceed to traverse through all the cells where any occurrence of the Panthera genus was
registered.
To do so we call the attribute *cells*. This attribute is abstracted with lazy evaluation.
Therefore, to fetch all the associated data we need to convert the object into a list (or a partial list
using an iterator). The time features shows how much time it takes to extract all the information.

In [6]:

```
cells = list(jaguars.cells)

print("cells has %s elements"%len(cells))
```

The resulting list has cell instances, each one connected to other cells by the relation: ’IS
NEIGHBOUR OF’. Accessing their related cells is achieved by the method:

```
cell.getNeighbours(with_center=[Boolean],order=[Int])
```

where the parameter *with\_center* returns the center of the neighborhood, and the parameter
*order* the size (in number of cells) of the neighborhood.
In our case, we apply this method for each cell using a map function.

**Note:**
For a fast computation reduce the order level to 1

In [8]:

```
neighbours = map(lambda cell : cell.getNeighbours(with_center=True,order=1), cells)
```

Lambda expressions are part of the Python syntax and are used to create anonymous functions.
The *map-lambda* technique allows the definition of statements that are applied to all the elements
of a list, returning a new list of objects obtained by evaluating the lambda expression on every
element of the given list.

Along this tutorial, the use of the map-lambda technique is frequently
used. Whenever this expression comes it is recommended to read the form:

```
map(lambda x : <something involving x> , some_list)
```

As, *for all x in some\_list , do something involving x*. In the example above, the object neighbours
is a list of neighbouring cells obtained from the method getNeighbours available on each cell instance (i.e. each element of the cells list).

The neighbours object is a list of a list of cells. We need to reduce this nested list into a single one
(flatten) with all the resulting cell instances.

In [9]:

```
# remmeber that the + operator merges two lists
neighbours = reduce(lambda list_a , list_b : list_a + list_b, neighbours)
```

The resulting neighbours list now has 2497 Cell nodes. In the current implementation the
name of the Grid (where all the Cells are contained) is called *mex4km*. We can display the first
three elements as this:

In [10]:

```
neighbours[:3]
```

Out[10]:

```
[< Cell-mex4km id = 234457 >,
 < Cell-mex4km id = 234686 >,
 < Cell-mex4km id = 234684 >]
```

## Converting cells to local taxonomic trees¶

We obtain the Tree of Life inside each Cell node by extracting the occurrences inside each cell
(using the method 'occurrencesHere' ) and plugging them into the *TreeNeo* constructor. The name
*TreeNeo* is used because the storage backend is the Neo4j graph database.

In [11]:

```
from drivers.tree_builder import TreeNeo
cell_1 = neighbours[1]
tree_1 = TreeNeo(cell_1.occurrencesHere())
```

In [12]:

```
tree_1
```

Out[12]:

```
<LocalTree Of Life | Root: LUCA - n.count : 3- >
```

The `n.count` value indicates the number of total occurrences.
We can generate all the trees iteratively using a maping the `TreeNeo(cell.occurrencesHere())` through all neighbouring cells. This may take some time depending on the number of cells and number of occurrences on each cell. For reducing this time go to Selecting order of neighbourhood

In [14]:

```
sample_trees = map(lambda cell : TreeNeo(cell.occurrencesHere()),neighbours)
```

As in the last example, we can see basic information as object description. Here showing the
first four elements.
`sample_trees` is a list of Trees. We can display some first elements as before.

In [15]:

```
sample_trees[:4]
```

Out[15]:

```
[<LocalTree Of Life | No record available: - n.count : 0- >,
 <LocalTree Of Life | Root: LUCA - n.count : 3- >,
 <LocalTree Of Life | Root: LUCA - n.count : 151- >,
 <LocalTree Of Life | Root: LUCA - n.count : 5- >]
```

It is possi-
ble to have empty trees, when no occurrences were found. This is showed with the text: `No record available`

## Exploratory analysis on a single Tree¶

We select a tree in this example and explore informative data.

In [16]:

```
tree = sample_trees[1]
```

The object `tree` wraps the entire tree structure.
All `Tree` objects have as starting node the root of the Taxonomic Tree which represents all known life.

In [17]:

```
root = tree.node
```

`Root` node is similar to `Family` node, `Genus` node, etc. They all belong to the class: `TreeNode`.

We can access a specific child node with the prefix `to_[name of taxon]`.  
For example, accessing the node 'Animalia' can be done with:

In [18]:

```
root.to_Animalia
```

Out[18]:

```
<LocalTree | Kingdom: Animalia - n.count : 2- | AF: 0.05>
```

The resulting node (Animalia) is again of a `TreeNode` type. We can explore this node until we reach no children.

**Note:** There could be errors if the selected tree is different from this example. This could happen, for example, if a reduction in the size of the neighbourhood was done.

### Traverse by children nodes¶

We can concatenate this method until the children attribute is empty. If running Biospytial in
an interactive session (like a Jupyter notebook or iPython) we can use the key [TAB] to autocom-
plete and show the available nodes. For example, the family of rodents *Muridae*.

**Hint:**
If we are running Biospytial in an interactive session (like Jupyter notebook or iPython)we can use the key [TAB] to autocomplete and show the available nodes.

In [19]:

```
root.to_Animalia.to_Chordata.to_Mammalia.to_Rodentia.to_Muridae
```

Out[19]:

```
<LocalTree | Family: Muridae - n.count : 2- | AF: 0.05>
```

### Tree traversal by taxonomic level¶

The taxonomic levels (e.g. families, orders, etc) are stored as atributes
of the *TreeNeo* class.
For example, to see available `phyla` in this tree do:

In [20]:

```
tree.phyla
```

Out[20]:

```
[<LocalTree | Phylum: Chordata - n.count : 2- | AF: 0.05>,
 <LocalTree | Phylum: Magnoliophyta - n.count : 1- | AF: 0.05>]
```

and for some families inside this tree.

In [21]:

```
tree.families[:5]
```

Out[21]:

```
[<LocalTree | Family: Annonaceae - n.count : 1- | AF: 0.05>,
 <LocalTree | Family: Muridae - n.count : 2- | AF: 0.05>]
```

## Tree operations¶

Tree objects allow symbolic operations for adding (merging) and intersecting other tree ob-
jects. These operations are currently implemented as `sum (+)` and `intersection (&)` . These
operations can be applied to arbitrary number of trees and it is useful in comparative studies that
require the calculus of (α, β, γ)-diversity using a combination of these operation [89]. Mathemati-
cally, these operations are equivalent theoretic set operations acting at the occurrence level. As an
example consider the following:

Let `t1` and `t2` two trees from the list of `sampled trees`

In [22]:

```
t1 = sample_trees[1]
t2 = sample_trees[2]
```

### Addition¶

Adding trees is equivalent to merging them. That is, making the union of all the nodes (inter
nodes and leaves). The tree objects ( `TreeNode` and `TreeNeo` classes) allow the use of the + opera-
tion. For example, obtaining the merge tree of t1 and t2 is achieved with:
Suming two trees is equivalent to merge them

In [23]:

```
t3 = t1 + t2
```

For example the classes of t1 and t2 are:

In [24]:

```
t1.classes
```

Out[24]:

```
[<LocalTree | Class: Mammalia - n.count : 2- | AF: 0.05>,
 <LocalTree | Class: Magnoliopsida - n.count : 1- | AF: 0.05>]
```

In [25]:

```
t2.classes
```

Out[25]:

```
[<LocalTree | Class: Protosteliomycetes - n.count : 2- | AF: 0.05>,
 <LocalTree | Class: Myxomycetes - n.count : 112- | AF: 0.05>,
 <LocalTree | Class: Agaricomycetes - n.count : 4- | AF: 0.05>,
 <LocalTree | Class: Liliopsida - n.count : 8- | AF: 0.05>,
 <LocalTree | Class: Magnoliopsida - n.count : 25- | AF: 0.05>]
```

In [26]:

```
t3.classes
```

Out[26]:

```
[<LocalTree | Class: Protosteliomycetes - n.count : 2- | AF: 0.05>,
 <LocalTree | Class: Myxomycetes - n.count : 112- | AF: 0.05>,
 <LocalTree | Class: Agaricomycetes - n.count : 4- | AF: 0.05>,
 <LocalTree | Class: Mammalia - n.count : 2- | AF: 0.05>,
 <LocalTree | Class: Liliopsida - n.count : 8- | AF: 0.05>,
 <LocalTree | Class: Magnoliopsida - n.count : 26- | AF: 0.05>]
```

### Intersection¶

Intersection is applied through the & operation and it is equivalent to the intersection of sets
with the difference that it is only applied to the leaf nodes, that is, the Occurrence nodes. Once
the leaf nodes are selected, the algorithm propagates through the parent nodes until it reaches
the root node. To see the formalization of the data structure go to supplementary materials II. For
obtaining the intersection of two trees do:

In [27]:

```
t = t1 & t2
t
```

Out[27]:

```
<LocalTree Of Life | No record available: - n.count : 0- >
```

In this case, the intersection is empty because the Occurrences are overlaid in a regular lattice
that partition the space, i.e. the cells are disjoint. See supplementary materials II for a formal
definition.

### Efficient addition of trees from a list of cells¶

We can use the sum iteratively as in a folding sum to obtain a Tree object representing all the areas definied in a list of Cells.

```
big_tree = reduce(lambda a , b : a+b , sample_trees)
```

However this method is not efficient. In each step, a new tree is created and the internal logic
to generate the union of all the intermediate nodes can result in redundant calculations. It is much
faster to select first the occurrences for all the trees inside a list and plugin them into the TreeNeo
constructor. As in the example below.

In [28]:

```
# Fast version
ocs = map(lambda s : s.occurrences,sample_trees)
## ocs is a nested list
## ocs is a list of list. We need to flat this into  a single list of occurrences
ocs = reduce(lambda a,b : a + b, ocs)
big_tree = TreeNeo(ocs,cell_objects=neighbours)
```

In [29]:

```
big_tree
```

Out[29]:

```
<LocalTree Of Life | Root: LUCA - n.count : 110159- >
```

The resulting tree could be very large. In this case, the obtained tree ( `big_tree` ) comprises
374731 occurrences. Remember that this tree is the resulting union of all the local taxonomic trees
obtained from the neighbourhood of degree 4 around the cells where jaguars occurred.

## Selecting nodes from the Red List¶

We filter the Species nodes from the big\_tree that are present in the Red List of threatened
species. For doing this we simply match the names using regular expressions. Using more sophis-
ticated methods for data matching are out of the scope of the present example. We assume that
the Red List data (a CSV file) has been loaded into a data frame with the name redlist .

## RedList data¶

The Red List data can be downloaded from the official website of The IUCN Red List of Threatened species.

Due to its Terms of Use policy I am unauthorised to distribute this data.

## Note for the reviewers of the manuscript¶

To reproduce the results in the Jaguar example download all the threatened species for Mexico or contact me for private communication.

- Juan Escamilla Molgora (j.escamillamolgora[at]lancaster.ac.uk) (molgor[at]gmail.com)

In [30]:

```
import pandas as pd
rdlistfile_ = "/RawDataCSV/assessments.csv"
rdlisttaxfile_ = "/RawDataCSV/taxonomy.csv"
redlist = pd.read_csv(rdlistfile_)
#redlisttax = pd.read_csv(rdlisttaxfile_)
#redlistdat = pd.merge(redlist,redlisttax,on='internalTaxonId')
```

Then we extract the species name corresponding to all the red list categories.

In [31]:

```
categories = list(redlist.groupby(by=redlist.redlistCategory).redlistCategory.first())
print(categories)
```

In [32]:

```
categories
```

Out[32]:

```
['Critically Endangered', 'Endangered', 'Vulnerable']
```

In [33]:

```
## Obtain threatening species from the Red List
critical_sps = redlist[ (redlist.redlistCategory == categories[0]) 
                       | (redlist.redlistCategory == categories[1])
                       | (redlist.redlistCategory == categories[2])
                      ].scientificName.apply(str.lower)

## Filter Species nodes from the big_tree.
## we convert the species names to lower case to avaoid ambiguity.
protected_by_jaguar = map(lambda critical_sp : 
                          filter(lambda sp : critical_sp in sp.name.lower() , big_tree.species),
                        critical_sps)
## Remove empty lists
protected_by_jaguar = filter(lambda l : l != [], protected_by_jaguar)
## Flatten lists
threatened_species = reduce(lambda a,b : a + b ,protected_by_jaguar)
# Remove Species repetitions
threatened_species = list(set(threatened_species))
## Extract all corresponding occurrences and flatten list 
t_ocs = reduce(lambda l1,l2 : l1 + l2 , map(lambda l : l.occurrences, threatened_species))
## Instantiate new tree
threatened_tree = TreeNeo(t_ocs)
```

In [34]:

```
threatened_tree.species[:4]
```

Out[34]:

```
[<LocalTree | Specie: Bombus haueri Handlirsch, 1888 - n.count : 1- | AF: 0.05>,
 <LocalTree | Specie: Bombus medius Cresson, 1863 - n.count : 18- | AF: 0.05>,
 <LocalTree | Specie: Ara militaris (Linnaeus, 1766) - n.count : 27- | AF: 0.05>,
 <LocalTree | Specie: Baronia brevicornis rufodiscalis de la Maza & White, 1987 - n.count : 2- | AF: 0.05>]
```

The threatened\_tree is now a taxonomic tree that only includes the occurrences that match
the species names of the Red List. To calculate the percentage of threatened species contained in
the selected tree we can do:

In [61]:

```
## total number of critical endangered species
ncrit = len(critical_sps)
len(threatened_tree.species) / float(ncrit) * 100
```

Out[61]:

```
7.649723200805234
```

That is, $13.48\%$ of the threatened species are contained in the neighbouring regions where jaguars
had been registered. To see if this result is relevant we calculate the percentage of the covered area
with respect to the whole country. First we need to calculate the total area of the cells.

In [62]:

```
## Area of each cell
cell = cells[0]
```

## Reprojecting datasets¶

The default coordinate reference system (crs) in the dataset is in geographic coordinates with WGS84 datum (EPSG:4326).
The units of this crs is in degrees, therefore the calculated area is defined in squared degrees.
In order to account for areas and distances based in meters (or kilometers) we need to project the selected geometries
into an appropriate projected coordinate system.
We use here the Alberts Equal Area Conic projection. To use this projection we need to specify its parameters in a string using the Proj4 syntax.

In [116]:

```
projection_string = "+proj=aea +lat_1=14.5 +lat_2=32.5 +lat_0=24 +lon_0=-105 +x_0=0 +y_0=0 +ellps=GRS80 +datum=NAD83 +units=m +no_defs;"
```

In order to interpret and transform the coordinates we need to import some functions and run the following code:

In [115]:

```
from shapely.ops import transform
from shapely import wkt,wkb
import pyproj
from functools import partial
```

In [139]:

```
mex_eq_area_proj = pyproj.Proj(projection_string)
## The WGS84 crs is defined as EPSG:4326
proj_in = pyproj.Proj(init='epsg:4326')
## function to project using the parameters of original_projection and the mexican equal area. 
project = partial(
    pyproj.transform,
    proj_in,
    mex_eq_area_proj)


## Transform all cells to calculate area.
projected_neighbours_cells = map(lambda cell : transform(project,cell.polygon_shapely),neighbours)
```

In [135]:

```
projected_cell.area / 1000000
```

Out[135]:

```
27.598191540266217
```

In [150]:

```
areas = map(lambda cell : cell.area, projected_neighbours_cells)
```

In [156]:

```
np.std(areas)/1000000
```

Out[156]:

```
2.7425646193084576
```

In [153]:

```
np.mean(areas) / 1000000
```

Out[153]:

```
27.62925657424348
```

In [157]:

```
## neighbours is the list of cells.
## Each cell has a polygon attribute with an area method, therefore:
tokm2 = 1000000
areas = map(lambda cell : cell.area, projected_neighbours_cells)
total_cell_area = sum(areas)
np.mean(areas) / tokm2
np.std(areas)/ tokm2
```

Out[157]:

```
2.7425646193084576
```

In [163]:

```
total_cell_area / 1000000
```

Out[163]:

```
8509.811024866996
```

Assuming that the world borders dataset is installed, we can import the polygon of Mexico
with the API provided by the class Country located in sketches.models. Country is a vector
dataset stored in the RDBMS. The geometric feature (column) is stored as geom.

In [145]:

```
from sketches.models import Country
## The syntax follows the Django Query Set API 
mexico = Country.objects.filter(name='Mexico').first()
mex_area = mexico.geom.area

## For reprojecting the area of Mexico we similarly do:
mex_shapely = wkt.loads(mexico.geom.wkt)
mex_projected= transform(project,mex_shapely)
```

To calculate the percentage of area covered by all the cells with respect with the total area of Mexico

In [164]:

```
total_cell_area / mex_projected.area * 100
```

Out[164]:

```
0.4347230971071755
```

In [304]:

```
from raster_api.tools import RasterData
from raster_api.models import raster_models
```

In [306]:

```
raster_models
```

Out[306]:

```
[raster_api.models.ETOPO1,
 raster_api.models.Precipitation,
 raster_api.models.SolarRadiation,
 raster_api.models.MeanTemperature,
 raster_api.models.MinTemperature,
 raster_api.models.MaxTemperature,
 raster_api.models.VaporPressure,
 raster_api.models.WindSpeed,
 raster_api.models.WorldPopLatam2010,
 raster_api.models.DistanceToRoadMex]
```

In [333]:

```
## Instantiate the raster model

whole_environmental_data = map(lambda raster_model : RasterData(rastermodelinstance=raster_model,border=mexico.geom),raster_models)
## Get the data from the  lazy instance
#MexPrec.getRaster()
```

In [ ]:

```
whole_datasets = []
for raster_model in raster_models:
    rd = RasterData(rastermodelinstance=raster_model,border=mexico.geom)
    rd.getRaster()
    rd.display_field()
    whole_datasets.append(rd)
```

In [733]:

```
## Visualise One
elevation = RasterData(rastermodelinstance=raster_models[0],border=mexico.geom)
```

In [736]:

```
elevation.getRaster()
elevation.plotField?
```

We can conclude that the regions with known presence of jaguars are approximately five times
more likely to include other threatened species than places without the presence of jaguars,
assuming that the rest of the threatened species are distributed evenly in rest of the country.

## Trimming trees¶

In certain situations we need to select a particular branch of a tree. We can cut (trim) this
branch by simply selecting a node and convert it into a TreeNeo instance for featuring a full feature
tree. The method (function) for converting a TreeNode into a full feature tree is: `plantTreeNode` .
We focus our attention in three branches of the threatened tree that co-occurrs with the presence
of jaguars. These branches are: mammals (class Mammalia), parrots (order Psittaciformes) and
amphibians (class Amphibia)

### Select the branch of interest¶

Trimming the tree is achieved by first selecting the nodes of interest and then converting all the
descendant branches into fully featured trees. There is no restriction for selecting the taxonomic
type of the node (mammals and amphibians are Class type while parrots are Order type).

In [39]:

```
mammals = threatened_tree.to_Animalia.to_Chordata.to_Mammalia
parrots = threatened_tree.to_Animalia.to_Chordata.to_Aves.to_Psittaciformes
amphibians = threatened_tree.to_Animalia.to_Chordata.to_Amphibia
plants = threatened_tree.to_Plantae.plantTreeNode()
```

The method `plantTreeNode()` converts the TreeNode and resulting descendants into a full fea-
tured tree (TreeNeo object).

In [40]:

```
mammals = mammals.plantTreeNode()
parrots = parrots.plantTreeNode()
amphibians = amphibians.plantTreeNode()
```

We can add all these trees together using the sum operation.

```
vertebrates = mammals + parrots + amphibians
```

However, as explained earlier, an optimized version for summing more than two trees is achieved
by instantiating a TreeNeo with all the occurrences.

In [41]:

```
vertebrates = TreeNeo(mammals.occurrences + parrots.occurrences + amphibians.occurrences)
```

The total number of occurrences contained in the *vertebrates* tree is:

In [42]:

```
vertebrates
```

Out[42]:

```
<LocalTree Of Life | Root: LUCA - n.count : 842- >
```

### Ranking the most frequent families in the selected list of cells¶

We proceed now to rank some groups according to their frequency of occurrence within the
cells of the study area (i.e. the jaguar’s neighbouring cells). The ranking analysis calculates this
frequency for each node in a tree given a referential list of trees. That is, assuming that we have
$n$ different trees (e.g. one per cell), and a tree of interest (in this case `threatened_tree` ) how
frequent does each node in this tree (e.g threatened\_trees ) appear in the list of $n$ trees?
In our implementation, this analysis is performed with the method: `countNodesFrequenciesOnList(list_of_trees)`.
That is:

In [43]:

```
vertebrates.countNodesFrequenciesOnList(list_of_trees=sample_trees)
mammals.countNodesFrequenciesOnList(list_of_trees=sample_trees)
parrots.countNodesFrequenciesOnList(list_of_trees=sample_trees)
amphibians.countNodesFrequenciesOnList(list_of_trees=sample_trees)
```

Out[43]:

```
0.05
```

In [682]:

```
plants.countNodesFrequenciesOnList(list_of_trees=sample_trees)
```

Out[682]:

```
0.05
```

In [695]:

```
plants.rankLevels()
plants.genera[:10]
```

Out[695]:

```
[<LocalTree | Genus: Tillandsia - n.count : 3- | AF: 0.198051948052>,
 <LocalTree | Genus: Lonchocarpus - n.count : 5- | AF: 0.181818181818>,
 <LocalTree | Genus: Eugenia - n.count : 1- | AF: 0.146103896104>,
 <LocalTree | Genus: Trichilia - n.count : 2- | AF: 0.116883116883>,
 <LocalTree | Genus: Coccoloba - n.count : 1- | AF: 0.103896103896>,
 <LocalTree | Genus: Chamaedorea - n.count : 8- | AF: 0.0974025974026>,
 <LocalTree | Genus: Oreopanax - n.count : 6- | AF: 0.0974025974026>,
 <LocalTree | Genus: Pouteria - n.count : 3- | AF: 0.0909090909091>,
 <LocalTree | Genus: Quercus - n.count : 3- | AF: 0.0681818181818>,
 <LocalTree | Genus: Ouratea - n.count : 2- | AF: 0.0681818181818>]
```

We can therefore rank by taxonomic level. In this example we show the procedure for family
and species level in the different branches. Here, we show the corresponding top five nodes.

In [693]:

```
vertebrates.rankLevels()
vertebrates.orders[:5]
```

Out[693]:

```
[<LocalTree | Order: Rodentia - n.count : 48- | AF: 0.37012987013>,
 <LocalTree | Order: Chiroptera - n.count : 35- | AF: 0.347402597403>,
 <LocalTree | Order: Carnivora - n.count : 16- | AF: 0.311688311688>,
 <LocalTree | Order: Anura - n.count : 129- | AF: 0.24025974026>,
 <LocalTree | Order: Psittaciformes - n.count : 55- | AF: 0.230519480519>]
```

In [45]:

```
mammals.rankLevels()
mammals.families[:5]
```

Out[45]:

```
[<LocalTree | Family: Muridae - n.count : 8- | AF: 0.308441558442>,
 <LocalTree | Family: Phyllostomidae - n.count : 8- | AF: 0.292207792208>,
 <LocalTree | Family: Cervidae - n.count : 14- | AF: 0.159090909091>,
 <LocalTree | Family: Heteromyidae - n.count : 3- | AF: 0.155844155844>,
 <LocalTree | Family: Tayassuidae - n.count : 158- | AF: 0.152597402597>]
```

In [692]:

```
parrots.rankLevels()
parrots.genera[:5]
```

Out[692]:

```
[<LocalTree | Genus: Amazona - n.count : 28- | AF: 0.168831168831>,
 <LocalTree | Genus: Ara - n.count : 27- | AF: 0.0422077922078>]
```

In [46]:

```
parrots.rankLevels()
parrots.to_Animalia.to_Chordata.to_Aves.to_Psittaciformes.to_Psittacidae.to_Amazona
```

Out[46]:

```
[<LocalTree | Specie: Ara militaris (Linnaeus, 1766) - n.count : 27- | AF: 0.0194805194805>,
 <LocalTree | Specie: Amazona finschi (P. L. Sclater, 1864) - n.count : 23- | AF: 0.0162337662338>,
 <LocalTree | Specie: Amazona auropalliata (Lesson, 1842) - n.count : 3- | AF: 0.00649350649351>,
 <LocalTree | Specie: Amazona oratrix Ridgway, 1887 - n.count : 2- | AF: 0.00324675324675>]
```

In [47]:

```
amphibians.rankLevels()
amphibians.families[:5]
```

Out[47]:

```
[<LocalTree | Family: Hylidae - n.count : 128- | AF: 0.152597402597>,
 <LocalTree | Family: Plethodontidae - n.count : 160- | AF: 0.0584415584416>,
 <LocalTree | Family: Eleutherodactylidae - n.count : 1- | AF: 0.0162337662338>,
 <LocalTree | Family: Caeciliidae - n.count : 7- | AF: 0.00974025974026>,
 <LocalTree | Family: Ambystomatidae - n.count : 2- | AF: 0.00324675324675>]
```

We can do the same analysis over the whole tree to see which groups, are more frequent

In [165]:

```
threatened_tree.countNodesFrequenciesOnList(list_of_trees=sample_trees)
```

Out[165]:

```
0.05
```

In [177]:

```
threatened_tree.classes
```

Out[177]:

```
[<LocalTree | Class: Elasmobranchii - n.count : 15- | AF: 0.00324675324675>,
 <LocalTree | Class: Amphibia - n.count : 298- | AF: 0.25>,
 <LocalTree | Class: Actinopterygii - n.count : 6- | AF: 0.133116883117>,
 <LocalTree | Class: Aves - n.count : 238- | AF: 0.522727272727>,
 <LocalTree | Class: Reptilia - n.count : 78- | AF: 0.324675324675>,
 <LocalTree | Class: Mammalia - n.count : 489- | AF: 0.522727272727>,
 <LocalTree | Class: Liliopsida - n.count : 27- | AF: 0.529220779221>,
 <LocalTree | Class: Magnoliopsida - n.count : 218- | AF: 0.694805194805>,
 <LocalTree | Class: Insecta - n.count : 21- | AF: 0.253246753247>,
 <LocalTree | Class: Pinopsida - n.count : 3- | AF: 0.038961038961>,
 <LocalTree | Class: Cycadopsida - n.count : 3- | AF: 0.0194805194805>]
```

## Associated environmental information¶

Here, we demonstrate how to access raster data associated with a taxonomic tree TreeNeo . The
raster data used are related to environmental variables stored in the RGPU. Currently there are two
ways to access this information:

1. as a table with columns corresponding to environmental variables and rows defined by each occurrence (a point-based method)
2. as a raster object sampled
   from the associated geometry of each tree. In this case, the output is a proper raster object that
   features methods for visualisation, geoprocessing data exchange, among other.
   To extract the data in table format we use the method (function):

In [400]:

```
env_threated_occurrences = threatened_tree.associatedData.getEnvironmentalVariablesPoints()
## Null data is assigned as -99999 see Biospytial documentation.
env_threated_occurrences = env_threated_occurrences.replace(-9999,np.nan)
env_threated_occurrences.dropna(inplace=True)
```

In [196]:

```
import seaborn as sns
sns.set(style="whitegrid")
```

In [295]:

```
## get environmental data from neighbour cells
environment_neighbours = map(lambda cell : cell.getEnvironmentalData(),neighbours)
env_neighbors = environment_neighbours.replace('N.A.',np.nan)
env_neighbours = env_neighbors.dropna()
```

In [433]:

```
## Aggregate and convert to Pandas  i.e. yearly average
whole_env_dataframes = map(lambda data : data.toPandasDataFrame(with_coordinates=True),whole_datasets)
whole_data = pd.concat(whole_env_dataframes,axis=1)
#whole_data = whole_data.dropna()
#whole_data.reset_index(drop=True,inplace=True)
```

In [434]:

```
precs = pd.concat([whole_data.Precipitation_m,
                   env_threated_occurrences.Precipitation_mean,
                   env_neighbours.Precipitation_m,
                  ],
                  axis=1)
precs.columns = ['All Mexico', 'Threatened occurrences','Jaguar\'s habitat']

sns.violinplot(data=precs)
```

Out[434]:

```
<matplotlib.axes._subplots.AxesSubplot at 0x7fac0a34fdd0>
```

In [435]:

```
meantemp = pd.concat([whole_data.MeanTemp_m,
                   env_threated_occurrences.MeanTemperature_mean,
                   env_neighbours.MeanTemp_m,
                  ],
                  axis=1)
meantemp.columns = ['All Mexico', 'Threatened occurrences','Jaguar\'s habitat']

sns.violinplot(data=meantemp)
```

Out[435]:

```
<matplotlib.axes._subplots.AxesSubplot at 0x7fac0a3d5610>
```

In [436]:

```
data_bundle = pd.concat([whole_data.WindSp_m,
                   env_threated_occurrences.WindSpeed_mean,
                   env_neighbours.WindSp_m,
                  ],
                  axis=1)
data_bundle.columns = ['All Mexico', 'Threatened occurrences','Jaguar\'s habitat']

sns.violinplot(data=data_bundle)
```

Out[436]:

```
<matplotlib.axes._subplots.AxesSubplot at 0x7fac0a43bcd0>
```

In [437]:

```
data_bundle = pd.concat([whole_data.VaporPres_m,
                   env_threated_occurrences.Vapor_mean,
                   env_neighbours.VaporPres_m,
                  ],
                  axis=1)
data_bundle.columns = ['All Mexico', 'Threatened occurrences','Jaguar\'s habitat']

sns.violinplot(data=data_bundle)
```

Out[437]:

```
<matplotlib.axes._subplots.AxesSubplot at 0x7fac0a4a9790>
```

The geometric object of each tree is determined by the Occurrence nodes of the tree. In the
graph database, each Occurrence node is linked to the Cell node that geographically contains
the occurrence’s location. One of the attributes of the Cell object is the geographic polygon that
defines its border. The union of all the corresponding Cell nodes is what determines the geomet-
ric feature of the tree TreeNeo . As such, the raster extraction process is performed on each of the
tree’s associated cells.
To extract the associated raster object of a TreeNeo use the method (function):

```
associatedData.getAssociatedRasterAreaData([name of variable])
```

To obtain several environmental variables use:

```
associatedData.getEnvironmentalVariablesCells()
```

For example, information for a single variable can be obtained with:

In [50]:

```
meantemp_data = vertebrates.associatedData.getAssociatedRasterAreaData('MeanTemperature')
```

The raster object is automatically added to the TreeNeo object after the method is called. The
raster objects are appended to the feature associatedData . For example, we can display simple
visualisations invoquing the method: `display_field()` .

### Interactive visualisation¶

As an alternative, we can export the raster object as an xarray instance for an interactive visu-
alisation using the Geoviews (http:geoviews.org) package.
To export the associated raster data to an xarray object do:

In [656]:

```
meantemp = vertebrates.associatedData.raster_MeanTemperature.to_xarray()
```

In [441]:

```
elevation = whole_datasets[0]
```

In [442]:

```
elevation = elevation.to_xarray()
```

For an interactive visualisation import the necessary packages and use:

In [717]:

```
import geoviews as gv
from cartopy import crs
import geoviews.feature as gf
from geoviews import opts
gv.extension('bokeh')
```

In [718]:

```
opts.defaults(
    opts.Image(width=1000, height=800, colorbar=True))

sample_pt = gv.Points((env_threated_occurrences.x,env_threated_occurrences.y),label='ocurrences').opts(
            fill_color = 'orange',
            line_color = 'black',
            line_width = 0.5,
            line_alpha = 0.4,
            fill_alpha = 1.0,
            size = 5,
            )


subset = elevation.where(((elevation.Longitude > -100) & (elevation.Longitude < -89) & 
                     (elevation.Latitude > 15) & (elevation.Latitude < 21)),drop=True)
#subset.name = elevation.name
#gvds = gv.Dataset(subset,crs=crs.PlateCarree())
elevds = gv.Dataset(elevation,crs=crs.PlateCarree())
#image1 = gvds.to(gv.Image,['Longitude','Latitude']).opts(cmap=plt.cm.terrain)
elevimg = gvds.to(gv.Image,['Longitude','Latitude']).opts(cmap=plt.cm.gist_earth)


temp = meantemp.where(((meantemp.Longitude > -95) & (meantemp.Longitude < -89) & 
                     (meantemp.Latitude > 15) & (meantemp.Latitude < 19)),drop=True)
temp.name = meantemp.name
tempds = gv.Dataset(temp,crs=crs.PlateCarree())
#gv1 = gv.Dataset(meantemp,crs=crs.PlateCarree())
#image1 = gvds.to(gv.Image,['Longitude','Latitude']).opts(cmap=plt.cm.terrain)
tempimg = tempds.to(gv.Image,['Longitude','Latitude']).opts(cmap=plt.cm.magma)
```

In [719]:

```
map_ = (elevimg * gf.ocean * gf.coastline * gf.borders * tempimg * sample_pt )
```

In [ ]:

```
map_.opts(title="Threatened species co-occurring with jaguars (Panthera Onca)")
```

# Network visualisation and analysis¶

Each tree instance induces an acyclic graph. We can convert the tree into a networkx object
to visualise and analyse its network properties. To do this, we simply need to use the method:
tree.toNetworkx(depth\_level=[k]) where k is the taxonomic level to reach in the tree, 0 for
root 7 for species level.

## Visualisation¶

A method for interactive visualisation has been developed using the Holoviews ( h ttps://holoviews.org)
framework. To do we need to invoke the method:

In [721]:

```
from drivers import tools
```

In [722]:

```
## Plot the Tree

#network = tools.to_interactivePlot(threatened_tree,label_depth=8,variable_nodes=False,node_size=20,
#                                   variable_labels=True,label_size=1)

network = tools.to_interactivePlot(threatened_tree,label_depth=8,variable_nodes=True,node_size=5,
                                  variable_labels=False,label_size=10,depth=4,cmap=plt.cm.viridis)
network['graph'] * network['labels']
```

Out[722]:

### Visualising the Parrots tree¶

In [ ]:

```
network = tools.to_interactivePlot(parrots,label_depth=8,variable_labels=False,label_size=10)
network['labels'] * network['graph']
```

### Visualising the mammals tree¶

In [724]:

```
network = to_interactivePlot(mammals,label_depth=8)
network['labels'] * network['graph']
```

Out[724]:

In [ ]:

```
## Plot the Tree

network = tools.to_interactivePlot(threatened_tree,label_depth=7)
network['labels'] * network['graph']
```

### Graph algorithms¶

The *TreeNeo* structures are particular cases of graph traversals. As such, they can be analysed with graph theoretic methods. The library `NetworkX` (https://networkx.github.io/) is a Python package designed for analysing structure, dynamics and functions of complex networks. It includes standard graph algorithms and analysis measures as well as tools for import and export to other standard formats.
We can convert a *TreeNeo* using the method: `toNetworkx(depth_level )`.
where depth\_level is the depth of the graph to be generated.

In the next example we convert the `threatened_tree` to a NetworkX object and use this to calculate its corresponding adjancecy matrix.

In [58]:

```
threatened_graph = threatened_tree.toNetworkx(depth_level=7)
```

In [59]:

```
from networkx import adjacency_matrix
M = adjacency_matrix(threatened_graph)
# uncomment this to plot the matrix
plt.imshow(M.todense())
```

Out[59]:

```
<matplotlib.image.AxesImage at 0x7fab526e2890>
```

Representing *TreeNeo* objects into NetworkX graphs brings new possibilities for analyses and modelling.
We hope this example will awake the spirit of the reader to explore the potential of representing data as complex graph structures.
