# Supplementary figures and images for: Biospytial: spatial graph-based computing for ecological Big Data
Source: Gigascience. 2020 May 11;9(5):giaa039. doi: 10.1093/gigascience/giaa039 (PMC7213554; doi:10.1093/gigascience/giaa039)

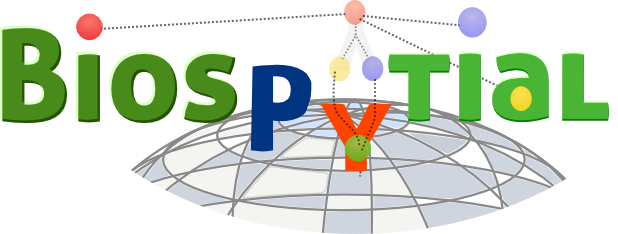

Supplement: giaa039_Supplemental_Files [file giaa039_supplemental_files.zip › biospytial.png]
