# Supplementary material for: Biospytial: spatial graph-based computing for ecological Big Data
Source: Gigascience. 2020 May 11;9(5):giaa039. doi: 10.1093/gigascience/giaa039 (PMC7213554; doi:10.1093/gigascience/giaa039)
Supplement: giaa039_Response_to_Reviewer_Comments_Original_Submission [file giaa039_response_to_reviewer_comments_original_submission.pdf]

Author's Response To Reviewer Comments

Close

Dear Dr Nicole Nogoy,
Editor of GigaScience and reviewers

Object: Manuscript GIGA-D-19-00265 “Biospytial: spatial graph-based computing engine for ecological big data”.

We are grateful to the reviewers for their comments which helped in improving the quality of the manuscript. We also thank the Editor for giving us the opportunity to revise the manuscript. We apologize for the delayed response and we thank you and the reviewers for your patience during this long process also.
We took on board all the comments as shown in the point to point reply to reviewers starting in the next page. The software is publicly available via github, and the data will be upload in SciCrunch shortly.

We hope these corrections satisfactorily address the various comments made by the Reviewers. Please let us know if any further corrections are required.

Yours sincerely,

Juan Escamilla Molgora on behalf of all the authors.

Responses to the reviewers

Notes:
Paragraphs that start with >> are our responses.
The format of the text is in Markdown and a PDF version of this text is included in this submission.

The new additions have been highlighted in the updated manuscript.

Reviewer 1

I very much enjoyed reading this paper and as a geospatial data scientist/GIScientist am very happy to see this considered for GigaScience.
I found the paper to be extremely comprehensive, very well-written, sound it computational approach, and reflecting a good knowledge of ecoinformatics that supports the global ecological community. The system architecture described is quite exciting. I admit to a lower level of expertise where knowledge engines are concerned, but know of many colleagues in the geospatial community who will be excited to see this new engine for **ecological data** that incorporates semantic relations and integrates into the geospatial semantic web.
Unfortunately these colleagues do not read GigaScience, but I will guide them appropriately. :-)

>> We thank the reviewer for the very positive general comment on our paper. We do hope to attract new readers to GigaScience.

Elaborate more discussions around the SDI I found it interesting that the authors chose "spatial data infrastructure" as their first key word. I think I know where they are heading with this, and this is an important connection to make, but might they consider adding a big more to the introduction or

concluding sections of the paper to make a stronger connection to the traditional "SDI" community? For instance, I am wondering how their engine qualifies as an SDI in the more traditional sense of the term. A traditional SDI implements a broader framework of geographic data, metadata, standards, institutional arrangements, policies, and tools that are interactively connected in order to make the use of spatial data more efficient and flexible. European INSPIRE is a shining example of this, as well as the United Nation's SDI, and the Convention on Biological Diversity. Could the authors briefly add how Biospytial is aiding in SDI beyond just employing GBIF or IUCN Red List data as a use case?

>>We fully agree with this vision of SDI and we thank the reviewer for raising this valid point. We have contextualised our engine in the light of current SDI definition. See lines 151-156.

By the way, how in the world does one properly pronounce "biospytial" (long y or short y). Could the authors provide a hint, if nothing else, just in their response to me as a reviewer. :-) In my mind I kept wanting to say "biospatial" with an "a!"

>>This comment is very useful, we have explained why we called the engine biospytial and how to pronunce it. See lines: 148 and 149.

Another very minor observation: the authors identify their GPU (Geospatial Processing Unit). So many of us are used to understanding GPU in the already established Graphics Processing Unit parlance of computer hardware terminology.

>>We have changed to RGU (relational geoprocessing unit) instead of using GPU.

More importantly, a major strength of this research is the interweaving of so many open source/open science technologies. I love Table 1 (a veritable "who's who"). And I applaud the use of containerization. I couldn't agree more with the statement on Line 172 that the idea here is to move the processes around, NOT the big data. I would add that the idea behind geospatial cloud computing, writ large, is to move the spatial analyses TO the data, rather than downloading or moving big data sets around. I mention this for discussion sake, not necessarily as a required change of wording in the paper.

>> We fully agree with this comment, and we extend our sentence in line 191-193 in order to stress the importance of performing the analyses where the data is located. See new line aaa.

What I *do* further suggest for minor changes have to do with the authors' important mention of reproducibility and replicability. Reproducibility is first mentioned on Line 17, and if I am understanding the authors' intent, I would like to suggest a few more references that they might consider consulting and adding:

* Barba LA. 2018. Praxis of reproducible computational science. Authorea: doi: 10.22541/au.153922477.77361922. doi:10.22541/au.153922477.77361922.
* Jasny BR, Wigginton N, McNutt M, Bubela T, Buck S, et al. 2017. Fostering reproducibility in industry-academia research. Science 357(6353): 759.
* Teytelman L. 2018. No more excuses for non-reproducible methods. Nature 560: 411. doi: 10.1038/d41586-018-06008-w.
* Shannon J, Walker K. 2018. Opening GIScience: A process-based approach. International Journal of Geographical Information Science 32(10): 1911-1926, doi: 10.1080/13658816.2018.1464167.

On Lines 170 and 224 they talk about *replicating* their applications and/or analysis. Do they mean reproduce instead? In the literature there is now an important distinction between reproducibility (a condition where results or products can be continually reproduced using the same data and methods) and

replicability (a higher level of scientific rigor where results or products can be reproduced using different samples of data and different software). In addition to the references above a good primer is now at https://sgsup.asu.edu/sites/default/files/rr_workshop_sparc_summary.pdf, as part of a recent workshop at https://sgsup.asu.edu/sparc/RRWorkshop. This part is a digression, just for discussion only: I think we can all acknowledge that a "reproducibility crisis" has received widespread attention across the sciences, but perhaps nowhere as much as in psychology, where numerous attempts to reproduce previous findings have failed. It can be argued that scientists generally lack the relevant skills and tools to ensure that their findings are reproducible and replicable, and that much academic literature amounts to little more than advertising of findings, rather than detailed reporting that would allow results to be reproduced and replicated. "Show me" should be more important than "trust me" in the culture of science. Efforts to build a culture of open science, in which data, tools, methods, and software are all made accessible to everyone, are welcome. But openness in and of itself is not sufficient to ensure that results can be reproduced, let alone replicated.

>>We fully agree with these comments and we thank the reviewer for sending us the references and workshop links. They were very useful and we have added a paragraph in the conclusions recognising the limitations in reproducibility and replicability in spatial analyses. We also added the Barba 2019, Teytelman and Shannon references. See lines 733 - 740.

Detected typos Lines 85-86 - the full definition of the GEO BON acronym is Group on Earth Observations Biodiversity Observation Network (they are missing the BON part)

>>Done.

Line 92 - I may have missed it, but can the authors please expand the PREDICTS acronym?

>>Done.

Congratulations again to the authors for this fine work and best wishes to them for continued success.

>> Thanks for your words we are really pleased with your comments and advices.

Reviewer 2

1.What classification algorithms are used to construct the tree?

>>The taxonomic classification mentioned in various points in the manuscript, is based on the classical natural systematic classification of the species in the tree of life. Therefore this classification already exists and it is used here to organize the taxa in a hierarchical structure. To remove any confusion we added a reference of the updated taxonomic classification used here.

2.Spatial stratified heterogeneity (SSH) becomes a serious problem when data is big and diverse. A sample is biased to SSH population when the sample don't cover all strata; and statistics become confounded when they are applied globally to SSH population. Therefore, SSH should be tested at early stage of big spatial data analysis. IF SSH is insignificant, a global model is safe; otherwise, a simple solution is to apply a model in strata, separately.

>>We thank the reviewer for this very good point. We have added a reference (Wang et al 2016) in line 87 recognising the important role of stratification to reduce bias in spatial analyses.

3.To illustrate the robust of the tool, the authors may provide several different examples for readers to practice. For example, cities evolution tree, besides the tree in the paper.

>>We understand the importance of robustness of our proposed engine. We have shown that any spatial data with an existing structure can be employed in our engine. We believe that adding other examples is out of the scope. The algorithm is open and we hope that others will build up the portfolio of applications. However, we have added additional components to the jaguar example to fully show its aplicability.

4.To increase tool's users who are unfamiliar with computer language, draw a flowchart so a user can follow as he/she is doing in the real world.

>>A full updated working example is provided towards the end of the paper.

Review 3

Overall This manuscript presents a knowledge engine designed to manage large spatial ecological data in a variety of formats and in an efficient way, using graph theory to maximise this efficiency, and enabling a series of operations. The manuscript is essentially composed of two sections, one in which the engine is described, and another one where its potential and applicability are shown in the form of meaningful examples.The paper is well written and structured, and presents a tool that can be of great use to ecologists and natural scientists, as well as to conservation managers with a natural sciences background. I suggest only some minor revisions, several of them asking for clarifications.

The jaguar example ends somewhat abruptly. In order to make a stronger point in showing the potential of the presented knowledge engine, it would be good if the authors would add a paragraph rounding up the results obtained in the exercise e.g. the reader ends up not being presented with the taxa most associated with the jaguar, or if these taxa were expected or else they are surprising. Linked to this, it would be interesting to follow up with the potential of the environmental layers to describe if the areas with jaguar are exceptional or not climatically, or topographically, or both, within Mexico. In other words, your example ends too quickly and more could be shown of it towards the end that would increase the perception of the reader regarding the potential and usefulness of the engine you present.

>> We have added a new section in the manuscript (section 4.3) describing the taxa and environmental results for jaguars.

Linked to the above, you could be more creative with figure 6, and figure 7 is nice but too messy. Consider showing a subset, and discuss it more in the text.

>>Both figures have been modified. Figure 8 (formerly 6) shows the elevation map (DEM) as base map displaying occurrences as points and environmental raster data objects as small overlapping regions. In figure 9 (formerly 7) we reduced the tree to only include orders, classes, phyla and kingdoms which reduces the number of nodes drastically. We improved the readability by assigning same size for all nodes' labels. We changed the color of the nodes representing the frequency (abundance) of taxa.

In page 24 and after, the codes include the term 'lambda' in many lines, and no explanation is given as to what that means. Could you specify what it means? Is it an anonymous function? In any case, this paper will be read by biologists with no background on computing science and the terms should be specified clearly.

>>Full explanation of the lambda functions and the joint effect of the map-lambda expression was added. See lines 506 - 513 and lines: 519 - 522.

In page 32, the total area of the cells is computed. It is not clear what is the original area of each cell.

>> We did the following amendments (lines: 599 - 608):
* Added subsection for reprojecting to conic equal area for measuring areas in meters.
* Added subsection for importing polygon from Mexico with reprojection.
* Added total area calculation and average size for each cell.

The GBIF database is composed of points (coordinates). How is this translated to a cell of a given area? Are you using the 1' DEM or the 1km environmental layers? In any case, GBIF coordinates can be of varying reliability, and a buffer is normally advised. It would be good to know where the cell area comes from (it was not too clear in the manuscript as it stands), and whether the coordinates in GBIF are taken as precise points. See this as a reference: https://onlinelibrary.wiley.com/doi/full/10.1111/ele.12624

>> We use the precise location (lat, lon wgs84 coordinates) of the GBIF occurrences given by their GBIF API / data (line 387). The occurrences are aggregated according to their taxonomy given that each occurrence belongs to a certain species. Although this was explained in lines: 403 - 407, we acknowledge that it was not clear enough. We added a more comprehensive explanation of the process for generating the local taxonomic trees on lines: 408 to 418. In addition, the procedure for aggregating occurrences through all the different taxonomic levels is described in supplementary materials II.

>> We thank the reviewer for pointing out the lack of clarity in the used grid. We included a brief description on how the grid system is created with a reference to the functions that generate customized grids (Lines: 397-399). In addition, we included a more explicit description of the grid used in 'worked example' under a new section named: 'Additional data used' (lines: 436-440).

>> We agree on the importance of estimating multidimensional biases, gaps and uncertainties in opportunistic samplings and citizen science records such as GBIF. For this reason we decided to use the complete information of location (point coordinates) of every record. However, accounting for these problems in the current worked example is out of the scope of the engine at this moment. We are, however, optimistic that the engine will help identify better this limitations of the data with the use of automatic or semi automatic procedures applied to large volumes of occurrences. Nevertheless, we thank the reviewer for the suggested reference as this is an issue that hopefully could be tackled in further applications of the engine.

The paragraph starting in line 511 contains a conclusion that is very difficult to sustain, since it is based on the assumption that threatened species are evenly distributed across the country. We know that this is not the case. I would be less categorical with it (i.e. it would seem that jaguars occur in places where other threatened species tend to cluster). The 'five times more

likely' is not believable given the assumption.

>> Agreed, the paragraph is misleading or meaningless with the presented assumption. It has been removed.

In general, the use of numbers for references is fine, but in some instances, it is strange: e.g. in cases where you refer to a citation in the form "...[20] proposed that...", it would help the readability to add "Smith et al. [20] proposed that..."

>> The numbering system for references have been changed to author names plus year.

Detected typos
>> All suggestions were covered. We thank the reviewer for her/his time and positive feedback.

Close
