# Supplementary material for: Biospytial: spatial graph-based computing for ecological Big Data
Source: Gigascience. 2020 May 11;9(5):giaa039. doi: 10.1093/gigascience/giaa039 (PMC7213554; doi:10.1093/gigascience/giaa039)
Supplement: giaa039_Response_to_Reviewer_Comments_Revision_1 [file giaa039_response_to_reviewer_comments_revision_1.pdf]

Author's Response To Reviewer Comments

Close

GIGA-D-19-00265R1
Biospytial: spatial graph-based computing for ecological big data

Dear Editor,

These are the point-by-point responses to the second revision.

1. All references have been changed to numbers (in order of appearance) according to the journal style (Vancouver).

2. The software has been registered in Scicrunch.org and bio.tools. The corresponding Resource id (RRID) and unique Biotools.id are now included in section 7: "Availability of supporting source code and requirements".

3. The supporting data has been registered in the GigaDB.org repository (http://gigadb.org/dataset/100723). The corresponding DOI link has been added in section 8: "Availability of supporting data".

4. The GigaDB repository has also been included in the list of references as:
Escamilla Molgora JM; Sedda L; Atkinson P (2020): Supporting data for "Biospytial: spatial graph-based computing engine for ecological big data" GigaScience Database. http://dx.doi.org/10.5524/100723

5. The list of abbreviations, in alphabetical order, has been included as section 9.

We want to thank the editor Nicole Nogoy and the GigaDB curator Chris Armit for their support, patience and positive feedback along this process.

with best wishes,

Juan Escamilla Molgora, on behalf of the co-authors.

Close
