# Supplementary material for: Biospytial: spatial graph-based computing for ecological Big Data
Source: Gigascience. 2020 May 11;9(5):giaa039. doi: 10.1093/gigascience/giaa039 (PMC7213554; doi:10.1093/gigascience/giaa039)
Supplement: giaa039_Reviewer_1_Report_Original_Submission — Dawn Wright -- 8/4/2019 Reviewed [file giaa039_reviewer_1_report_original_submission.pdf]

Reviewer Report

Title: Biospytial: spatial graph-based computing for ecological big data

Version: Original Submission **Date:** 8/4/2019

Reviewer name: Dawn Wright

Reviewer Comments to Author:

I very much enjoyed reading this paper and as a geospatial data scientist/GIScientist am very happy to see this considered for GigaScience. I found the paper to be extremely comprehensive, very well-written, sound it computational approach, and reflecting a good knowledge of ecoinformatics that supports the global ecological community. The system architecture described is quite exciting. I admit to a lower level of expertise where knowledge engines are concerned, but know of many colleagues in the geospatial community who will be excited to see this new engine for **ecological data** that incorporates semantic relations and integrates into the geospatial semantic web. Unfortunately these colleagues do not read GigaScience, but I will guide them appropriately. :-)
I found it interesting that the authors chose "spatial data infrastructure" as their first key word. I think I know where they are heading with this, and this is an important connection to make, but might they consider adding a big more to the introduction or concluding sections of the paper to make a stronger connection to the traditional "SDI" community? For instance, I am wondering how their engine qualifies as an SDI in the more traditional sense of the term. A traditional SDI implements a broader framework of geographic data, metadata, standards, institutional arrangements, policies, and tools that are interactively connected in order to make the use of spatial data more efficient and flexible. European INSPIRE is a shining example of this, as well as the United Nation's SDI, and the Convention on Biological Diversity. Could the authors briefly add how Biospytial is aiding in SDI beyond just employing GBIF or IUCN Red List data as a use case?
By the way, how in the world does one properly pronounce "biospytial" (long y or short y). Could the authors provide a hint, if nothing else, just in their response to me as a reviewer. :-) In my mind I kept wanting to say "biospatial" with an "a!"
Another very minor observation: the authors identify their GPU (Geospatial Processing Unit). So many of us are used to understanding GPU in the already established Graphics Processing Unit parlance of computer hardware terminology.
More importantly, a major strength of this research is the interweaving of so many open source/open science technologies. I love Table 1 (a veritable "who's who"). And I applaud the use of containerization. I couldn't agree more with the statement on Line 172 that the idea here is to move the processes around, NOT the big data. I would add that the idea behind geospatial cloud computing, writ large, is to move the spatial analyses TO the data, rather than downloading or moving big data sets around. I mention this for discussion sake, not necessarily as a required change of wording in the paper.
What I *do* further suggest for minor changes have to do with the authors' important mention of reproducibility and replicability. Reproducibility is first mentioned on Line 17, and if I am understanding the authors' intent, I would like to suggest a few more references that they might consider consulting

and adding:
Barba LA. 2018. Praxis of reproducible computational science. Authorea: doi: 10.22541/au.153922477.77361922. doi:10.22541/au.153922477.77361922.
Jasny BR, Wigginton N, McNutt M, Bubela T, Buck S, et al. 2017. Fostering reproducibility in industry-academia research. Science 357(6353): 759.
Teytelman L. 2018. No more excuses for non-reproducible methods. Nature 560: 411. doi: 10.1038/d41586-018-06008-w.
Shannon J, Walker K. 2018. Opening GIScience: A process-based approach. International Journal of Geographical Information Science 32(10): 1911-1926, doi: 10.1080/13658816.2018.1464167.
On Lines 170 and 224 they talk about *replicating* their applications and/or analysis. Do they mean reproduce instead? In the literature there is now an important distinction between reproducibility (a condition where results or products can be continually reproduced using the same data and methods) and replicability (a higher level of scientific rigor where results or products can be reproduced using different samples of data and different software). In addition to the references above a good primer is now at https://sgsup.asu.edu/sites/default/files/rr_workshop_sparc_summary.pdf, as part of a recent workshop at https://sgsup.asu.edu/sparc/RRWorkshop.
This part is a digression, just for discussion only: I think we can all acknowledge that a "reproducibility crisis" has received widespread attention across the sciences, but perhaps nowhere as much as in psychology, where numerous attempts to reproduce previous findings have failed. It can be argued that scientists generally lack the relevant skills and tools to ensure that their findings are reproducible and replicable, and that much academic literature amounts to little more than advertising of findings, rather than detailed reporting that would allow results to be reproduced and replicated. "Show me" should be more important than "trust me" in the culture of science. Efforts to build a culture of open science, in which data, tools, methods, and software are all made accessible to everyone, are welcome. But openness in and of itself is not sufficient to ensure that results can be reproduced, let alone replicated.
Final minor suggestions:
Lines 85-86 - the full definition of the GEO BON acronym is Group on Earth Observations Biodiversity Observation Network (they are missing the BON part)
Line 92 - I may have missed it, but can the authors please expand the PREDICTS acronym?
Congratulations again to the authors for this fine work and best wishes to them for continued success.

Level of Interest

Please indicate how interesting you found the manuscript: Choose an item.

Quality of Written English

Please indicate the quality of language in the manuscript: Choose an item.

Declaration of Competing Interests

Please complete a declaration of competing interests, considering the following questions:

- Have you in the past five years received reimbursements, fees, funding, or salary from an organisation that may in any way gain or lose financially from the publication of this manuscript, either now or in the future?
- Do you hold any stocks or shares in an organisation that may in any way gain or lose financially from the publication of this manuscript, either now or in the future?
- Do you hold or are you currently applying for any patents relating to the content of the manuscript?
- Have you received reimbursements, fees, funding, or salary from an organization that holds or has applied for patents relating to the content of the manuscript?
- Do you have any other financial competing interests?
- Do you have any non-financial competing interests in relation to this paper?

If you can answer no to all of the above, write 'I declare that I have no competing interests' below. If your reply is yes to any, please give details below.

I declare that I have no competing interests.

I agree to the open peer review policy of the journal. I understand that my name will be included on my report to the authors and, if the manuscript is accepted for publication, my named report including any attachments I upload will be posted on the website along with the authors' responses. I agree for my report to be made available under an Open Access Creative Commons CC-BY license (http://creativecommons.org/licenses/by/4.0/). I understand that any comments which I do not wish to be included in my named report can be included as confidential comments to the editors, which will not be published.

Choose an item.

To further support our reviewers, we have joined with Publons, where you can gain additional credit to further highlight your hard work (see: https://publons.com/journal/530/gigascience). On publication of this paper, your review will be automatically added to Publons, you can then choose whether or not to claim your Publons credit. I understand this statement.

Yes Choose an item.
