# Supplementary material for: Biospytial: spatial graph-based computing for ecological Big Data
Source: Gigascience. 2020 May 11;9(5):giaa039. doi: 10.1093/gigascience/giaa039 (PMC7213554; doi:10.1093/gigascience/giaa039)
Supplement: giaa039_Reviewer_2_Report_Original_Submission — Jinfeng Wang -- 9/18/2019 Reviewed [file giaa039_reviewer_2_report_original_submission.pdf]

Reviewer Report

Title: Biospytial: spatial graph-based computing for ecological big data

Version: Original Submission **Date:** 9/18/2019

Reviewer name: Jinfeng Wang

Reviewer Comments to Author:

1. What classification algorithms are used to construct the tree?
2. Spatial stratified heterogeneity (SSH) becomes a serious problem when data is big and diverse. A sample is biased to SSH population when the sample don't cover all strata; and statistics become confounded when they are applied globally to SSH population. Therefore, SSH should be tested at early stage of big spatial data analysis. IF SSH is insignificant, a global model is safe; otherwise, a simple solution is to apply a model in strata, separately.
3. To illustrate the robust of the tool, the authors may provide several different examples for readers to practice. For example, cities evolution tree, besides the tree in the paper.
4. To increase tool's users who are unfamiliar with computer language, draw a flowchart so a user can follow as he/she is doing in the real world.

Level of Interest

Please indicate how interesting you found the manuscript: Choose an item.

Quality of Written English

Please indicate the quality of language in the manuscript: Choose an item.

Declaration of Competing Interests

Please complete a declaration of competing interests, considering the following questions:

- Have you in the past five years received reimbursements, fees, funding, or salary from an organisation that may in any way gain or lose financially from the publication of this manuscript, either now or in the future?
- Do you hold any stocks or shares in an organisation that may in any way gain or lose financially from the publication of this manuscript, either now or in the future?
- Do you hold or are you currently applying for any patents relating to the content of the manuscript?
- Have you received reimbursements, fees, funding, or salary from an organization that holds or has applied for patents relating to the content of the manuscript?
- Do you have any other financial competing interests?

- Do you have any non-financial competing interests in relation to this paper?

If you can answer no to all of the above, write 'I declare that I have no competing interests' below. If your reply is yes to any, please give details below.

I declare that I have no competing interests

I agree to the open peer review policy of the journal. I understand that my name will be included on my report to the authors and, if the manuscript is accepted for publication, my named report including any attachments I upload will be posted on the website along with the authors' responses. I agree for my report to be made available under an Open Access Creative Commons CC-BY license (http://creativecommons.org/licenses/by/4.0/). I understand that any comments which I do not wish to be included in my named report can be included as confidential comments to the editors, which will not be published.

Choose an item.

To further support our reviewers, we have joined with Publons, where you can gain additional credit to further highlight your hard work (see: https://publons.com/journal/530/gigascience). On publication of this paper, your review will be automatically added to Publons, you can then choose whether or not to claim your Publons credit. I understand this statement.

Yes Choose an item.
