# Supplementary material for: Biospytial: spatial graph-based computing for ecological Big Data
Source: Gigascience. 2020 May 11;9(5):giaa039. doi: 10.1093/gigascience/giaa039 (PMC7213554; doi:10.1093/gigascience/giaa039)
Supplement: giaa039_Reviewer_3_Report_Original_Submission — Marc Macias-Fauria -- 10/6/2019 Reviewed [file giaa039_reviewer_3_report_original_submission.pdf]

**Reviewer Report**

**Title: Biospytial: spatial graph-based computing for ecological big data**

**Version: Original Submission** **Date:** 10/6/2019

**Reviewer name: Marc Macias-Fauria**

**Reviewer Comments to Author:**

Overall
This manuscript presents a knowledge engine designed to manage large spatial ecological data in a variety of formats and in an efficient way, using graph theory to maximise this efficiency, and enabling a series of operations. The manuscript is essentially composed of two sections, one in which the engine is described, and another one where its potential and applicability are shown in the form of meaningful examples. The paper is well written and structured, and presents a tool that can be of great use to ecologists and natural scientists, as well as to conservation managers with a natural sciences background.
I suggest only some minor revisions, several of them asking for clarifications.
Concrete Points
The jaguar example ends somewhat abruptly. In order to make a stronger point in showing the potential of the presented knowledge engine, it would be good if the authors would add a paragraph rounding up the results obtained in the exercise: e.g. the reader ends up not being presented with the taxa most associated with the jaguar, or if these taxa were expected or else they are surprising. Linked to this, it would be interesting to follow up with the potential of the environmental layers to describe if the areas with jaguar are exceptional or not climatically, or topographically, or both, within Mexico. In other words, your example ends too quickly and more could be shown of it towards the end that would increase the perception of the reader regarding the potential and usefulness of the engine you present.
Linked to the above, you could be more creative with figure 6, and figure 7 is nice but too messy. Consider showing a subset, and discuss it more in the text.
In page 24 and after, the codes include the term 'lambda' in many lines, and no explanation is given as to what that means. Could you specify what it means? Is it an anonymous function? In any case, this paper will be read by biologists with no background on computing science and the terms should be specified clearly.
In page 32, the total area of the cells is computed. It is not clear what is the original area of each cell. The GBIF database is composed of points (coordinates). How is this translated to a cell of a given area? Are you using the 1' DEM or the 1km environmental layers? In any case, GBIF coordinates can be of varying reliability, and a buffer is normally advised. It would be good to know where the cell area comes from (it was not too clear in the manuscript as it stands), and whether the coordinates in GBIF are taken as precise points. See this as a reference: https://onlinelibrary.wiley.com/doi/full/10.1111/ele.12624
The paragraph starting in line 511 contains a conclusion that is very difficult to sustain, since it is based on the assumption that threatened species are evenly distributed across the country. We know that this is not the case. I would be less categorical with it (i.e. it would seem that jaguars occur in places where

other threatened species tend to cluster). The 'five times more likely' is not believable given the assumption.

Typos

In general, the use of numbers for references is fine, but in some instances, it is strange: e.g. in cases where you refer to a citation in the form "…[20] proposed that…", it would help the readability to add "Smith et al. [20] proposed that…"

Line 72: "Answering this question…" and not "Answer this question…"

Line 147: "Section 4 explores…" and not "Section 4, explores…"

Line 156: "…class instances ([48] and [42])" and not "…class instances [48] and [42]"

Line 165: "(GSPU)" and not "(GSU)"

Line 174: "(RGU)" and not "(RGP)"

Figure 1: in (b), the figure reads "Biospytial Computing Unit" and it should read "Biospytial Computing Engine"

Line 230: "In our implementation, the…" and not "In our implementation the…"

Line 234: "…we call knowledge graph" and not "…we call: knowledge graph"

Line 238: "figure 2" and not "figure: 2" (and others like this throughout the text)

Line 270: "In the example given in figure 2, the…" and not "In the example given in figure 2 the…"

Line 271: "…is name…" and not "…is: name…"; "value P.mocinno" and not "value: P.mocinno"

Line 280: "…Random Fields ([62] [63] and [64])" and not "…Random Fields [62] [63] and [64]"

Line 299: "…Culicidae…" and not "…Cullicidae…"

Line 302: "…supports and processes…" and not "…supports and process…"

Line 350: "…(ii) the territory…" and not "…(ii) The territory…"

Line 351: "…(ii) the country…" and not "…(ii) The country …"

Lines 358, 359, 493: in general, it increases the readability of large numbers (10,000 and above), to add a comma in the thousands and millions (e.g. 3,242,746, or 10,781, etc.)

Line 361: "…location (coordinates in…" instead of "…location, (coordinates in…"

Line 362: "…acquisition date, and represents…" instead of "…acquisition date and represents…"

Line 377: "…standardised by [79]" and not "…standardised by: [79]"

Line 378: "Each instance comprised of…" instead of "Each instance composed of…"

Line 467: "…of these operations" and not "…of these operation"

Expression after line 526: 'birds' and not 'birs'. Also, why 'frogs' in here? It is the only place, they are amphibians later. And then you shift from birds to parrots. Should it be 'parrots' in here as well?

Line 543: "GRU" and not "GRPU"

Figure 6: "Month: April" and not "Months: April"

Line 611: "Biospytial" and not "Biopytial"

Line 994: "Biospytial" and not "Biosptial"

Line 1080: "…Species" and not "…Specie"

**Level of Interest**

Please indicate how interesting you found the manuscript: Choose an item.

**Quality of Written English**

Please indicate the quality of language in the manuscript: Choose an item.

**Declaration of Competing Interests**

Please complete a declaration of competing interests, considering the following questions:

- Have you in the past five years received reimbursements, fees, funding, or salary from an organisation that may in any way gain or lose financially from the publication of this manuscript, either now or in the future?
- Do you hold any stocks or shares in an organisation that may in any way gain or lose financially from the publication of this manuscript, either now or in the future?
- Do you hold or are you currently applying for any patents relating to the content of the manuscript?
- Have you received reimbursements, fees, funding, or salary from an organization that holds or has applied for patents relating to the content of the manuscript?
- Do you have any other financial competing interests?
- Do you have any non-financial competing interests in relation to this paper?

If you can answer no to all of the above, write 'I declare that I have no competing interests' below. If your reply is yes to any, please give details below.

No competing interests

I agree to the open peer review policy of the journal. I understand that my name will be included on my report to the authors and, if the manuscript is accepted for publication, my named report including any attachments I upload will be posted on the website along with the authors' responses. I agree for my report to be made available under an Open Access Creative Commons CC-BY license (http://creativecommons.org/licenses/by/4.0/). I understand that any comments which I do not wish to be included in my named report can be included as confidential comments to the editors, which will not be published.

Choose an item.

To further support our reviewers, we have joined with Publons, where you can gain additional credit to further highlight your hard work (see: https://publons.com/journal/530/gigascience). On publication of this paper, your review will be automatically added to Publons, you can then choose whether or not to claim your Publons credit. I understand this statement.

Yes Choose an item.
